# A permutation approach to the assignment of the configuration to diastereomeric tetrads by comparison of experimental and ab initio calculated differences in NMR data

**DOI:** 10.3762/bjoc.13.245

**Published:** 2017-11-22

**Authors:** Przemysław J Boratyński

**Affiliations:** 1 Department of Organic Chemistry, Wrocław University of Technology, Wyspiańskiego 27, 50-370 Wrocław, Poland

**Keywords:** GIAO, NMR, stereochemistry assignment

## Abstract

Scoring permutations of experimental chemical shift deviations and DFT/GIAO calculated deviations of isotropic shieldings for sets of four diastereomers can help to assign their relative configurations. This method was exercised on a set of diastereomeric *Cinchona* alkaloid derivatives, where ^13^C NMR data always identified the proper configuration. The presented approach is also an attempt to quantify the assignment by exclusion.

## Introduction

In a stereodivergent synthesis [1] often two or more new stereocenters are created with or without control of stereochemistry and it is necessary to identify the configuration of the products. Any diastereomers exhibit disparate NMR spectra, while an experiment employing the nuclear Overhauser effect can often help to assign a particular relative configuration. However, such a task is sometimes impossible due to spectral congestion or lack of diagnostic correlations. On the other hand, the availability of cheap computing power combined with capable software has led to inclusion of computational chemistry into the organic laboratory practice [2]. This provided a powerful tool to augment the interpretation of experimental data including NMR results [3–10]. Comparisons of experimental and theoretical spectra for the pairs of diastereomers were studied and used quite extensively [8,10].

So far, however, assignments of configuration to four diastereomeric compounds, i.e., tetrads, were mostly performed by separately comparing selected pairs against each other. However, the availability of multiple stereoisomers should translate to more robust assignment when all the data were analyzed at the same time. This is because surplus information should increase the statistical power and therefore improve the confidence in the assignment. This is particularly true since DFT calculated chemical shifts suffer from various systematic errors while the relative differences between the isomers can be predicted quite accurately [8]. Thus, the accuracy should further increase if the results of calculations were referenced to an average of more than two sets of data owing to statistics. Such an approach predating the common use of NMR computation has been applied for comparison of diastereomers against a universal database made of known compounds [11–12].

## Results and Discussion

The idea presented herein is to rank all possible permutations of experimental and computed data rather than focusing on individual pairs. The tetrads need to encompass all stereomers at two selected stereocenters. Thus all possible configurations, e.g., *RR*, *RS*, *SR*, and *SS* need to be distributed to four isomeric compounds. For four different items there are overall *P*_4_ = 4! = 24 possible permutations. In other words, there are 24 ways in which configurations could be assigned, only one of them correct. The use of permutations ensures that two different diastereomers cannot share the same configuration. Each of these permutations needs to be ranked by how well the experimental and computed data agree. The best match is then expected for a properly assigned configuration, while any swapped configuration should obtain an adequately lower score. The score gap between the best permutation and a second runner-up conveys the confidence in the assignment.

The entire process begins with DFT prediction of isotropic shieldings by one of the established literature procedures, e.g., [3,9], and signal assignment of the experimental NMR spectra. Often, a single ^1^H,^13^C-HSQC experiment was sufficient for complete assignment of ^13^C NMR data. In order to calibrate the data for comparison, for each atom an average (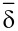) of corresponding chemical shifts in four diastereomers is calculated. Then differences (Δ_i_) between these averages and individual chemical shifts are calculated (Equation 1). The same approach is followed for calculated isotropic shieldings. In case of the latter a change of sign is necessary since shieldings are negatively proportional to shifts (Equation 2). This way listings of directly comparable Δ_i_ data are created.

[1]
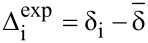


[2]
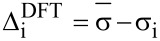


Where Δ_i_ is a chemical shift deviation for individual atom/signal, δ_i_ is a measured or calculated chemical shift; 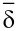 is an averaged chemical shift corresponding to an individual atom in all the stereomers; σ_i_ is the isotropic shielding obtained from DFT calculation.

Then, chemical shift listings are arranged into 24 different arrays corresponding to all permutations. For example data of experimental compounds **a**, **b**, **c**, and **d** are arranged to computed data for *RR*, *RS*, *SR*, and *SS* isomers in permutation 1, respectively, *RS*, *RR*, *SR*, and *SS* in permutation 2 and so on (cf. Table 2). A comparison is performed between the experimental and computed Δ_i_ data for each of the 24 arrays. There are a few different measures of comparison that can be applied, and some of them are listed in Table 1. These can represent difference as mean absolute error (MAE) or related root mean square deviation (RMS). Alternatively, an aggregate overlap of values can be calculated. Also comparison by covariance can be used by means of the Pearson correlation coefficient (R) or similar parameters such as CP1 and CP3 introduced by Smith and Goodman [8]. It has to be noted that for complete sets of Δ_i_ data some of these measures exhibit linear relationship. Finally the permutations are ordered according to their scores from highest to lowest or in the case of mismatch parameters (MAE, RMS) in an inverse order.

**Table 1:** Selected measures of comparison^a^.

entry	measure	applied formula

1	MAE	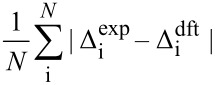
2	RMS	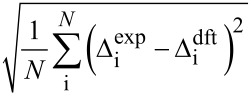
3	aggregate overlap	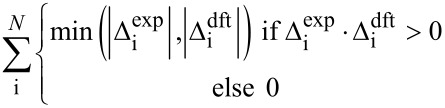
4	R	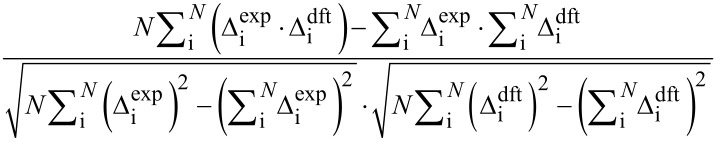
5	CP1	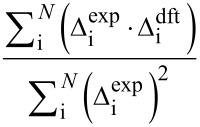
6	CP3	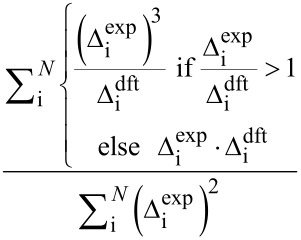

^a^Where Δ_i_ are individual deviations of chemical shifts given in Equation 1 and Equation 2 for corresponding atoms, *N* is the total number of signals in all four isomers. For terms with division of by zero in entry 6 assume zero result. Note: when complete sets are studied there is a linear relationship between measures in entries 1 and 3, as well as 4 and 5.

It is noteworthy that after the signal assignment is made, all the steps and numerical operations can be performed with the help of a simple computer program. An example code along with illustration of manual workflow is shown in Supporting Information File 1.

### Example application

**Figure 1:**
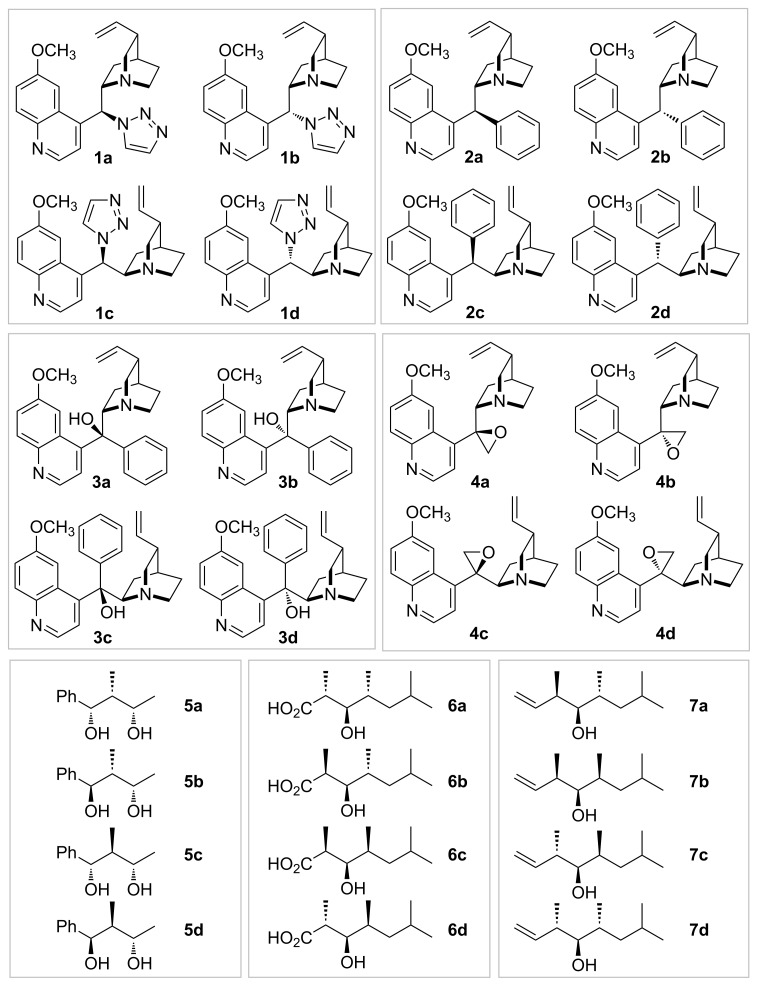
Studied tetrads **1**–**7**.

**Scheme 1:**
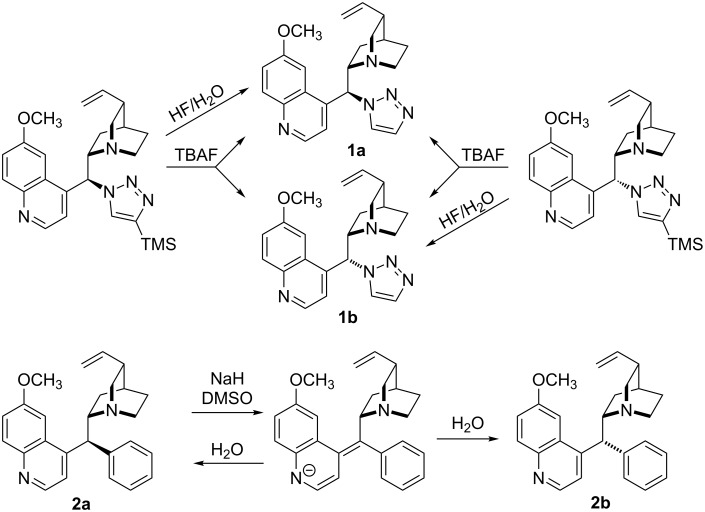
Synthesis and chemical correlation for compounds of type **1** and **2**.

In order to exercise the presented approach four (8*R*/*S*,9*R*/*S*) sets of derivatives of *Cinchona* alkaloids **1**–**4** obtained in our laboratory were analyzed (Figure 1, for references, see Supporting Information File 1). The configurations of these derivatives were established based on previous X-ray studies for compounds **2a** and **3d**, NOESY experiments combined with molecular modeling for compounds of type **4**, and chemical correlation for compounds **1a**–**d** (cf. Scheme 1). Also, three sets of matched computed and experimental data for alcohols **5**–**7** were taken directly from the report by Goodman and relevant experimental papers (Figure 1) [8,13–15].

Some previously not reported compounds of type **1** and **2** were obtained by epimerization at C9. Mixtures of the isomers of **1** were formed in TBAF desilylation of *Cinchona* 4-TMS-triazole derivatives. Partial isomerisation of **2a** into **2b** was performed by transient deprotonation using in situ generated sodium methylsulfinylmethylide (Scheme 1). Both mixtures of diastereomers were separable by chromatography. However, more than half of **2** was isolated in mixed fractions.

The NMR spectra in CDCl_3_ were assigned using ^1^H,^13^C-HSQC and HMBC experiments. For tetrad **4**, NOESY spectra were available and diastereotopic protons were distinguished. Otherwise, the geminal hydrogens were arranged by their chemical shifts. The computed geometries were all optimized at DFT/B3LYP/6-31G(d,p) in vacuum and GIAO calculations were performed at the same level of theory but including polarizable continuum solvent model (PCM) for chloroform utilizing Gaussian code [16]. The initial 6–9 geometries corresponded to rotation along major degrees of freedom, i.e., C4’–C9 and C9–C8 bonds and orientation of the pendant ring. Conformers of up to 3 kcal/mol higher in energy were considered in the calculation and their contribution was included in the Boltzmann-weighted average shieldings (Equation 3). The geometries for tetrads **1**–**3** were also reoptimized using the 6-311G+(2d,p) basis set and the GIAO calculation was redone using mPW1PW91 functional and the same basis set as recommended by Tantillo [3]

[3]
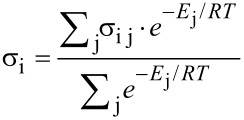


where *E*_j_ is the relative energy of conformer *j*, σ*_ij_* is isotropic shielding for atom *i* in conformer *j*.

An example of scoring of all permutations based on ^13^C NMR data for tetrad **1** is shown in Table 2 (for all data, see Supporting Information File 1).

**Table 2:** Permutation scores for the comparison of experimental ^13^C NMR chemical shifts deviations and DFT isotropic shieldings.^a^

entry	configuration				score^b^			

	**1a**	**1b**	**1c**	**1d**	R	CP3	overlap	RMS

1^c^	8*S*,9*S*	8*S*,9*R*	8*R*,9*R*	8*R*,9*S*	0.881	0.682	44.7	0.728
2	8*S*,9*S*	8*S*,9*R*	8*R*,9*S*	8*R*,9*R*	0.842	0.662	42.0	0.830
3	8*S*,9*R*	8*S*,9*S*	8*R*,9*R*	8*R*,9*S*	0.835	0.654	40.3	0.846
4	8*S*,9*R*	8*S*,9*S*	8*R*,9*S*	8*R*,9*R*	0.796	0.634	37.6	0.936
5	8*S*,9*S*	8*R*,9*S*	8*R*,9*R*	8*S*,9*R*	0.169	0.042	28.2	1.853
6	8*S*,9*S*	8*R*,9*R*	8*R*,9*S*	8*S*,9*R*	0.113	0.022	26.6	1.913
7	8*S*,9*R*	8*R*,9*S*	8*R*,9*R*	8*S*,9*S*	0.097	0.003	23.9	1.930
8	8*R*,9*S*	8*S*,9*S*	8*R*,9*R*	8*S*,9*R*	0.077	−0.056	22.8	1.951
9	8*R*,9*R*	8*S*,9*S*	8*R*,9*S*	8*S*,9*R*	0.059	−0.046	25.0	1.970
10	8*R*,9*S*	8*S*,9*R*	8*R*,9*R*	8*S*,9*S*	0.051	−0.067	22.9	1.978
11	8*S*,9*R*	8*R*,9*R*	8*R*,9*S*	8*S*,9*S*	0.042	−0.017	22.4	1.988
12	8*R*,9*R*	8*S*,9*R*	8*R*,9*S*	8*S*,9*S*	0.033	−0.057	25.1	1.997
13	8*S*,9*S*	8*R*,9*S*	8*S*,9*R*	8*R*,9*R*	−0.026	−0.266	23.4	2.057
14	8*S*,9*S*	8*R*,9*R*	8*S*,9*R*	8*R*,9*S*	−0.043	−0.267	24.5	2.073
15	8*S*,9*R*	8*R*,9*S*	8*S*,9*S*	8*R*,9*R*	−0.068	−0.277	21.1	2.097
16	8*S*,9*R*	8*R*,9*R*	8*S*,9*S*	8*R*,9*S*	−0.084	−0.277	22.2	2.113
17	8*R*,9*R*	8*S*,9*R*	8*S*,9*S*	8*R*,9*S*	−0.093	−0.317	24.9	2.122
18	8*R*,9*R*	8*S*,9*S*	8*S*,9*R*	8*R*,9*S*	−0.097	−0.335	22.9	2.126
19	8*R*,9*S*	8*S*,9*R*	8*S*,9*S*	8*R*,9*R*	−0.114	−0.346	20.1	2.142
20	8*R*,9*S*	8*S*,9*S*	8*S*,9*R*	8*R*,9*R*	−0.118	−0.364	18.0	2.146
21	8*R*,9*R*	8*R*,9*S*	8*S*,9*S*	8*S*,9*R*	−0.805	−0.957	8.5	2.721
22	8*R*,9*R*	8*R*,9*S*	8*S*,9*R*	8*S*,9*S*	−0.835	−0.986	6.5	2.744
23	8*R*,9*S*	8*R*,9*R*	8*S*,9*S*	8*S*,9*R*	−0.842	−0.986	4.7	2.749
24	8*R*,9*S*	8*R*,9*R*	8*S*,9*R*	8*S*,9*S*	−0.873	−1.015	2.8	2.772

^a^Permutations arranged by the value of Pearson correlation coefficient. ^b^Scores (cf. Table 1): R – Pearson correlation coefficient; CP3 – Smith and Goodman’s comparison parameter; overlap – aggregate overlap of datasets; RMS – root mean square difference. ^c^Correct assignment of configuration. For ^1^H NMR comparison table and for similar tables for compounds **2**–**7**, see Supporting Information File 1.

For all the compounds comparison of the ^13^C NMR data identified the permutations corresponding to proper configuration assignment with the highest scores (Table 3). This was found regardless of the measure used for comparison. The separation between the scores for the correct assignment and a following best permutation varied widely for different tetrads **1**–**7**. However, the separation was generally larger for sets where better agreement for the correct assignment was found. On the other hand, for two of the tested tetrads, namely **3** and **7**, the comparison of ^1^H NMR data did not identify the correct assignment with the highest score. The incorrect ^1^H assignments were characterized by relatively low values of highest correlation coefficients (0.71 and 0.47) compared to the coefficients (0.71–0.95) for properly identified tetrads **1**, **4**, **5**, and **6**. The order of lower ranking permutations was vastly different between ^1^H and ^13^C data. This indicates that both nuclei can be used for independent assignment of configuration. It has to be emphasized that the ^1^H NMR data for tetrads **1** and **4** were very predictive of configuration, as demonstrated by higher correlation coefficients than for ^13^C NMR data. These two compounds are less likely to form hydrogen bonds and specific interactions with the solvent, therefore their conformational equilibria are more likely to be accurately modeled by DFT calculation.

**Table 3:** Permutations with two highest scores for the comparison of experimental ^13^C NMR chemical shifts deviations and DFT isotropic shieldings.

entry	configuration				score			
					
					R	CP3	overlap	RMS

	**1a**	**1b**	**1c**	**1d**				
1^a^	8*S*,9*S*	8*S*,9*R*	8*R*,9*R*	8*R*,9*S*	0.881	0.682	44.7	0.728
2	8*S*,9*S*	8*S*,9*R*	8*R*,9*S*	8*R*,9*R*	0.842	0.662	42.0	0.830
	**2a**	**2b**	**2c**	**2d**				
3^a^	8*S*,9*S*	8*S*,9*R*	8*R*,9*R*	8*R*,9*S*	0.969	0.935	52.8	0.330
4	8*S*,9*S*	8*S*,9*R*	8*R*,9*S*	8*R*,9*R*	0.925	0.894	47.5	0.512
	**3a**	**3b**	**3c**	**3d**				
5^a^	8*S*,9*S*	8*S*,9*R*	8*R*,9*R*	8*R*,9*S*	0.895	0.781	64.1	0.719
6	8*S*,9*S*	8*S*,9*R*	8*R*,9*S*	8*R*,9*R*	0.726	0.599^b^	51.8	1.111
	**4a**	**4b**	**4c**	**4d**				
7^a^	8*S*,9*S*	8*S*,9*R*	8*R*,9*R*	8*R*,9*S*	0.960	0.912	61.5	0.411
8	8*S*,9*S*	8*S*,9*R*	8*R*,9*S*	8*R*,9*R*	0.677	0.613	48.2	1.161
	**5a**	**5b**	**5c**	**5d**				
9^a^	1*S*,3*R*	1*R*,3*R*	1*S*,3*S*	1*R*,3*S*	0.920	0.769	30.4	0.732
10	1*S*,3*R*	1*R*,3*S*	1*S*,3*S*	1*R*,3*R*	0.626	0.538	22.0	1.515
	**6a**	**6b**	**6c**	**6d**				
11^a^	2*R*,4*R*	2*S*,4*R*	2*S*,4*S*	2*R*,4*S*	0.657	0.479	23.3	1.193
12	2*R*,4*R*	2*S*,4*S*	2*S*,4*R*	2*R*,4*S*	0.424	0.297	21.1	1.517
	**7a**	**7b**	**7c**	**7d**				
13^a^	3*R*,5*R*	3*R*,5*S*	3*S*,5*S*	3*S*,5*R*	0.842	0.623	29.5	0.599
14	3*R*,5*R*	3*S*,5*S*	3*R*,5*S*	3*S*,5*R*	0.774	0.536	26.9	0.715

^a^Correct assignment of configuration ^b^For this parameter a different second highest scoring permutation exists. For the details, see Supporting Information File 1.

Reinforcing computational methods by extending the basis set to 6-311G++(2d,2p) for both optimization and NMR calculation steps, as well as applying the MPW1PW91 functional (compounds **1**–**3**) did not affect the comparison significantly. However, most of the comparison parameters for ^13^C NMR data were improved for the correct permutation. Also the separations between the scores for correct assignment and second best permutation were slightly increased according to the majority of measures.

### One compound missing

In order to address the scenario when not all diastereomers are available, for example due to inherent instability of one of them, an incomplete comparison was performed. For this purpose sets of experimental shifts for only three compounds were matched against four listings of DFT-derived data. All possible sets for compounds **1**–**7** were analyzed. It was then checked whether the highest ranked permutations corresponded to the correct assignment. It was found that in most cases the ^13^C NMR data correctly identified the proper configuration of the diastereomers. Unfortunately in this scenario the application of ^1^H NMR data would lead to a few incorrect assignments. The choice of comparison measures affected the result noticeably (Table 4). The lack of a fourth experimental data set introduces a bias in the average of experimental shifts. This particularly affects measures which overemphasize differences (such as CP3). On the other hand RMS deviation and Pearson correlation coefficient proved as the most effective scores. While assignment for the three out of four diastereomers was found to be mostly accurate, synthesis and inclusion of a “missing” isomer would noticeably improve the chance for a correct assignment (100% vs 92% for ^13^C, and 77% vs 62% for ^1^H) for the studied tetrads **1**–**7**.

**Table 4:** Percentage of correctly identified configuration by highest ranking permutation for sets of three out of four diastereomers of compounds **1**–**7**.

measure	^13^C NMR data	^1^H NMR data

CP1	96%	46%
CP2	79%	67%
CP3	89%	58%
overlap	89%	67%
RMS	100%	67%
correlation	100%	63%
MAE	96%	67%

## Conclusion

The presented approach using ^13^C NMR data led to a correct assignment of configuration to the sets of four or three diastereomers, alleviating the need for scaling of chemical shifts. By feeding all available NMR data into the algorithm a permutation with a highest rank is identified. The major limitation is how accurately the experiment is predicted by the DFT calculation. This is, however, a central problem and any data processing will fail in cases where the computed data do not agree with the experiment.

## Experimental

**9*R*-(4-Trimethylsilyl-1,2,3-triazol-1-yl)-9-deoxyquinidine:** To a solution of 9*R*-azido-9-deoxyquinidine (969 mg, 2.78 mmol) in a mixture of *tert*-butanol (18 mL) and water (9 mL) were added trimethylsilylacetylene (0.5 mL, 3.5 mmol, 1.3 equiv), CuSO_4_·5H_2_O (22 mg, 3 mol %), and sodium ascorbate (157 mg, 29 mol %). The mixture was stirred for 24 h, then a drop of saturated aqueous Na_2_S was added and the mixture was extracted with CH_2_Cl_2_, dried over MgSO_4_ and filtered through a pad (2 cm) of silica gel and evaporated. Evacuation on a rotary vane pump afforded 978 mg of the product with 10 mol % of residual *tert*-butanol as amorphous white solid (78%). ^1^H NMR (600 MHz, CDCl_3_) δ 8.76 (d, *J* = 4.6 Hz, 1H), 8.00 (d, *J* = 9.2 Hz, 1H), 7.54 (d, *J* = 4.6 Hz, 1H), 7.47 (d, *J* = 2.4 Hz, 1H), 7.47 (s, 1H), 7.37 (dd, *J* = 9.2, 2.4 Hz, 1H), 6.45 (br., 1H), 5.82 (ddd, *J* = 17.1, 10.6, 6.5 Hz 1H), 5.07 (d, *J* = 17.1 Hz, 1H), 5.05 (d, *J* = 10.6 Hz, 1H), 4.02–3.97 (m, 1H), 3.94 (s, 3H), 3.02–2.96 (m, 3H), 2.90–2.84 (m, 1H), 2.29–2.24 (m, 1H), 1.72–1.71 (m, 1H), 1.67–1.56 (m, 2H), 1.38–1.33 (m, 1H), 1.23–1.27 (s, 1H), 0.21 (s, 9H) ppm; ^13^C NMR (151 MHz, CDCl_3_) δ 158.7, 147.5, 146.3, 145.0, 140.39, 140.36, 132.1, 128.10, 128.00, 122.5, 119.9, 115.0, 100.7, 59.2 (br.), 58.4, 55.8, 49.4, 47.6, 39.2, 27.8, 26.5, 26.0, −1.0 ppm. HRMS–ESI–TOF (*m*/*z*): [M + H]^+^ calcd for C_25_H_34_N_5_OSi, 448.2527; found, 448.2521.

**9*S- *and 9*R*-(1,2,3-triazol-1-yl)-9-deoxyquinidine (1d and 1c):** 9*R*-(4-Trimethylsilyl-1,2,3-triazol-1-yl)-9-deoxyquinidine (329 mg, 0.77 mmol) was dissolved in THF (10 mL), then a solution of TBAF (1.1 mL, 1 M in THF, 1.4 equiv) was added, and the mixture was stirred at 60 °C for 28 h and evaporated. HRMS–ESI–TOF (*m*/*z*): [M + H]^+^ calcd for C_22_H_24_N_5_O, 376.2132; found, 376.2126. Chromatography on silica gel (CHCl_3_/MeOH, 20:1) afforded 128 mg of **1d** (46%), and 61 mg of **1c** (22%) as white amorphous solids.

**1c**: ^1^H NMR (600 MHz, CDCl_3_) δ 8.78 (d, *J* = 4.6 Hz, 1H), 8.02 (d, *J* = 9.2 Hz, 1H), 7.62 (s, 1H), 7.56 (s, 1H), 7.53 (d, *J* = 4.6 Hz, 1H), 7.47 (d, *J* = 2.6 Hz, 1H), 7.38 (dd, *J* = 9.2, 2.6 Hz, 1H), 6.51(d, *J* = 10.7 Hz, 1H), 5.85 (ddd, *J* = 17.1, 10.6, 6.5 Hz, 1H), 5.12 (dt, *J* = 17.1, 1.3 Hz, 1H), 5.09 (dt, *J* = 10.6, 1.4 Hz, 1H), 3.95 (s, 1H), 3.96–3.91 (m, 1H), 3.10–3.05 (m, 1H), 3.01–2.96 (m, 2H), 2.93–2.87 (m, 1H), 2.32–2.27 (m, 1H), 1.77–1.74 (m, 1H), 1.69–1.60 (m, 2H), 1.46–1.41 (m, 1H), 1.31–1.27 (m, 1H) ppm; ^13^C NMR (151 MHz, CDCl_3_) δ 158.7, 147.4, 145.0, 140.2, 139.7, 133.8, 132.1, 128.1, 122.5 (2C overlapped), 119.5, 115.0, 100.5, 59.6, 58.3, 55.8, 49.4, 47.2, 39.0, 27.6, 26.5, 26.2 ppm.

**1d**: ^1^H NMR (600 MHz, CDCl_3_) δ 8.82 (d, *J* = 4.6 Hz, 1H), 7.98 (d, *J* = 9.2 Hz, 1H), 7.63 (br., 1H), 7.59 (s, 1H), 7.34–7.29 (m, 3H), 6.45 (br. d, *J* = 10.4 Hz, 1H), 6.01 (ddd, *J* = 17.3, 10.4, 7.3 Hz, 1H), 5.12 (d, *J* = 10.4 Hz, 1H), 5.08 (d, *J* = 17.2 Hz, 1H), 3.88 (s, 3H), 3.90–3.86 (m, 1H), 3.09–3.04 (m, 1H), 2.92–2.82 (m, 2H), 2.73–2.68 (m, 1H), 2.31–2.26 (m, 1H), 1.82–1.79 (m, 1H), 1.70–1.61 (m, 3H), 1.21–1.15 (m, 1H) ppm. ^13^C NMR (151 MHz, CDCl_3_) *δ*: 158.5, 147.6, 145.1, 140.06, 140.02, 134.4, 132.1, 128.1, 122.46, 122.21, 119.7, 115.3, 100.5, 60.8, 57.5, 55.9, 49.3, 48.3, 39.8, 27.5, 26.4, 24.7 ppm.

**9*R*-(1,2,3-Triazol-1-yl)-9-deoxyquinine** (**1b**) was prepared analogously to **1d**: Starting from 1.81 g of 9*S*-(4-trimethylsilyl-1,2,3-triazol-1-yl)-9-deoxyquinine, 600 mg of pure **1b** (40%) and 542 mg (36%) of **1a** were obtained. Alternatively starting from 272 mg of 9*R*-(4-trimethylsilyl-1,2,3-triazol-1-yl)-9-deoxyquinine 97 mg of **1b** (42%) and 88 mg of **1a** (39%) were obtained both as amorphous solids. *R*_f_ (CHCl_3_:MeOH, 20:1): 0.34 for **1a**, 0.42 for **1b**.

**1b**: ^1^H NMR (600 MHz, CDCl_3_) δ 8.83 (d, *J* = 4.6 Hz, 1H), 7.99 (d, *J* = 9.2 Hz, 1H), 7.70 (d, *J* =4.6 Hz, 1H), 7.60 (s, 1H), 7.36 (d, *J* = 2.6 Hz, 1H), 7.31 (s, 1H), 7.30 (dd, *J* = 9.2, 2.6 Hz), 6.45 (d, *J* = 11.3 Hz, 1H), 5.89 (ddd, *J* = 17.3, 10.4, 7.3 Hz, 1H), 5.07 (dt, *J* = 17.3, 1.3 Hz, 1H), 5.05 (dt, *J* = 10.4, 1.4 Hz, 1H), 3.88 (s, 3H), 3.95–3.83 (m, 1H), 3.16 (dd, *J* = 13.6, 10.0 Hz, 1H), 2.93–2.84 (m, 2H), 2.66–2.60 (m, 1H), 2.35–2.29 (m, 1H), 1.85–1.82 (m, 1H), 1.77–1.72 (m, 1H), 1.57–1.52 (m, 2H), 1.37–1.33 (m, 1H) ppm; ^13^C NMR (151 MHz, CDCl_3_) δ 158.6, 147.6, 145.1, 141.7, 139.6, 134.6, 132.1, 128.0, 122.25, 122.20, 119.9, 114.9, 100.4, 61.4, 57.4, 56.0, 55.8, 41.5, 39.5, 27.7, 27.2, 25.2 ppm; HRMS–ESI–TOF (*m*/*z*): [M + H]^+^ calcd for C_22_H_26_N_5_O, 376.2132; found, 376.2141.

**9*R*-Phenyl-9-deoxyquinine (2b):** Under inert gas (Ar), a sodium hydride dispersion (60% in mineral oil, 195 mg, 4.9 mmol, 5 equiv) was dissolved in DMSO (9 mL) over 1.5 h at 65 °C and cooled to room temperature. Then a solution of 9*S*-deoxy-9-phenylquinine (**2a**, 395 mg, 1.03 mmol) in DMSO (1.8 mL) was added and the mixture stirred for 25 min. Then ice was added and the mixture diluted with Et_2_O (20 mL), and washed with brine (5 × 25 mL), dried over Na_2_SO_4_ and evaporated to give 322 mg of a **2a**:**2b** mixture, 38:62 by ^1^H NMR integration. Careful separation by chromatography (AcOEt/MTBE/MeOH, 10:10:1) gave 45 mg of pure **2b** (11%) as an off-white amorphous solid, the remaining **2b** was received in a mixture with **2a**. ^1^H NMR (300 MHz, CDCl_3_) δ 8.80 (d, *J* = 4.6 Hz, 1H), 7.96 (d, *J* = 9.2 Hz, 1H), 7.57 (d, *J* = 4.6 Hz, 1H), 7.42 (d, *J* = 2.6 Hz, 1H), 7.34 (d, *J* = 8.2 Hz, 2H), 7.27 (dd, *J* = 9.2, 2.6 Hz, 1H), 7.24 (t, *J* = 8.0 Hz, 2H), 7.13 (tt, *J* = 7.6, 1.3 Hz, 1H), 5.92 (ddd, *J* = 17.4, 10.2, 7.4 Hz,1H), 5.09–5.00 (m, 2H), 4.63 (d, *J* = 11.3 Hz, 1H), 3.90 (s, 3H), 3.73–3.63 (m, 1H), 3.19 (dd, *J* = 13.7, 9.9 Hz, 1H), 2.99–2.89 (m, 1H), 2.80–2.88 (m, 1H), 2.61–2.50 (m, 1H), 2.33–2.22 (m, 1H), 1.79–1.76 (m, 1H), 1.72–1.43 (m, 4H), 1.29–1.21 (m, 1H) ppm; ^13^C NMR (75.5 MHz, CDCl_3_) δ 157.5, 147.8, 146.9, 144.9, 142.1, 140.7, 131.9, 129.0, 128.6, 128.4, 126.9, 120.5, 119.7, 114.7, 102.0, 58.3, 56.2, 55.5, 50.2, 41.0, 39.8, 28.1, 28.0, 27.9 ppm.

## Supporting Information

File 1 Plots of NMR spectra for new compounds, HSQC experiments for tetrad **1**, supporting tables, complete reference [16], synthetic references for compounds **1**–**4**, presentation of manual workflow and Python code for automated processing.
