# Supplementary material for: A permutation approach to the assignment of the configuration to diastereomeric tetrads by comparison of experimental and ab initio calculated differences in NMR data
Source: Beilstein J Org Chem. 2017 Nov 22;13:2478–85. doi: 10.3762/bjoc.13.245 (PMC5704763; doi:10.3762/bjoc.13.245)
Supplement: File 1 — Plots of NMR spectra for new compounds, HSQC experiments for tetrad 1, supporting tables, complete reference [16], synthetic references for compounds 1–4, presentation of manual workflow and Python code for automated processing. [file Beilstein_J_Org_Chem-13-2478-s001.pdf]

Supporting Information

for

A permutation approach to the assignment of the configuration to diastereomeric tetrads by comparison of experimental and ab initio calculated differences in NMR data

Przemysław J. Boratyński*[1]

Address: [1]Department of Organic Chemistry, Wrocław University of Technology, Wyspiańskiego 27, 50-370 Wrocław, Poland

Email: Przemysław Boratyński - Przemyslaw.boratynski@pwr.wroc.pl

*Corresponding author

Plots of NMR spectra for new compounds, HSQC experiments for tetrad 1, supporting tables, complete reference 16, synthetic references for compounds 1–4, presentation of manual workflow and Python code for automated processing

Table of Contents

S1. Supporting references S2

S2. Manual workflow example S3

S3. Peripheral discussion S5

S4. Supporting tables S5

S4.1. Tables of experimental NMR shifts and GIAO shieldings S6

S4.2. Tables with all permutations and their scores S10

S5. Plots of ^{1}H and ^{13}C NMR spectra of new compounds S21

S5. Plots of ^{1}H,^{13}C HSQC experiments for tetrad **1** S26

S7. Computer program (python) for quick calculation of permutations and their scores S28

S8. Cartesian coordinates for gas phase optimized geometries of tetrads **1–3** S31

S1. Supporting references

Synthesis of **1a**:

Boratyński, P. J.; Kowalczyk, R. *J. Org. Chem.* **2016**, *81*, 8029-8034.

Synthesis of **2a** and **2c**:

Boratyński, P. J.; Turowska-Tyrk, I.; Skarżewski, J. *Org. Lett.* **2008**, *10*, 385–388.

Synthesis **2d** and **3a-d** including NMR calculation for tetrad **3**.

Boratyński, P. J.; Turowska-Tyrk, I.; Skarżewski, J. *Tetrahedron: Asymmetry* **2012,** *23*, 876-883.

Synthesis and NMR calculation of **4a-d**:

Boratyński, P. J.; Skarżewski, J. *J. Org. Chem.* **2013,** *78*, 4473-4482.

Complete Gaussian reference, Reference 16 from main text

Frisch, M. J.; Trucks, G. W.; Schlegel, H. B.; Scuseria, G. E.; Robb, M. A.; Cheeseman, J. R.; Scalmani, G.; Barone, V.; Mennucci, B.; Petersson, G. A.; Nakatsuji, H.; Caricato, M.; Li, X.; Hratchian, H. P.; Izmaylov, A. F.; Bloino, J.; Zheng, G.; Sonnenberg, J. L.; Hada, M.; Ehara, M.; Toyota, K.; Fukuda, R.; Hasegawa, J.; Ishida, M.; Nakajima, T.; Honda, Y.; Kitao, O.; Nakai, H.; Vreven, T.; Montgomery, J. A., Jr., Peralta, J. E.; Ogliaro, F.; Bearpark, M.; Heyd, J. J.; Brothers, E.; Kudin, K. N.; Staroverov, V. N.; Kobayashi, R.; Normand, J.; Raghavachari, K.; Rendell, A.; Burant, J. C.; Iyengar, S. S.; Tomasi, J.; Cossi, M.; Rega, N.; Millam, J. M.; Klene, M.; Knox, J. E.; Cross, J. B.; Bakken, V.; Adamo, C.; Jaramillo, J.; Gomperts, R.; Stratmann, R. E.; Yazyev, O.; Austin, A. J.; Cammi, R.; Pomelli, C.; Ochterski, J. W.; Martin, R. L.; Morokuma, K.; Zakrzewski, V. G.; Voth, G. A.; Salvador, P.; Dannenberg, J. J.; Dapprich, S.; Daniels, A. D.; Farkas, O.; Foresman, J. B.; Ortiz, J. V.; Cioslowski, J.; Fox, D. J. **Gaussian 09, E.01**, Gaussian, Inc.: Wallingford, CT, 2009.

S2. Manual workflow example

Here the approach is exemplified by conducting the entire process manually and stepwise on the example of three signals assigned to three atoms (C-6, C-7, and C-8) for diastereomeric tetrad **1**. For automatic processing, a simple computer program can be implemented, for example see Section S6.

Part A. Initial data

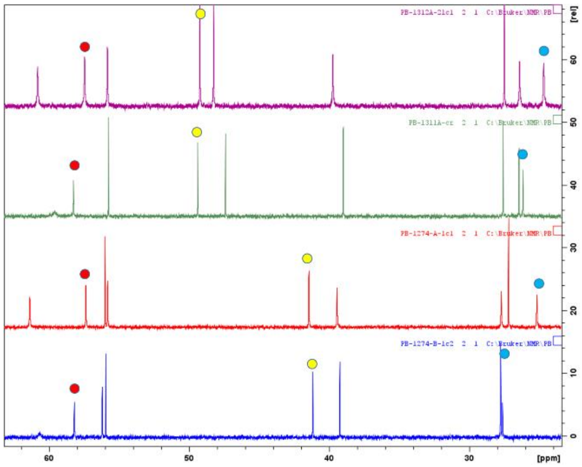

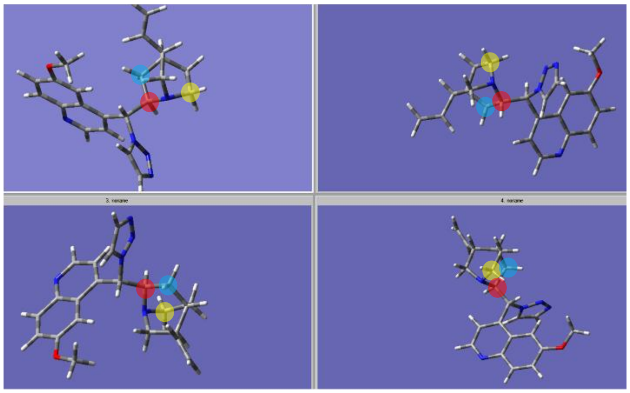

Interpretation of ^{13}C NMR data for four diastereomers **1a**-**1d**.
Assignment of chemical shifts to particular atoms.

Molecular models of diastereomers **1** of specified configurations (*SS*, *SR*, *RR*, *RS*) are made, their geometries are optimized. Populations of individual conformers are evaluated based on their energy.
Calculation of GIAO isotropic shieldings, e.g. at B3LYP/6-31G(d,p) level of theory or higher

experiment

atom		1a	1b	1c	1d	average	1a	1b	1c	1d
C-6	yellow	41.2	41.5	49.4	48.3	45.1	-3.9	-3.6	4.3	3.2
C-7	blue	27.7	25.2	26.2	24.7	25.9	1.7	-0.7	0.3	-1.2
C-8	red	58.2	57.4	58.3	57.5	57.8	0.4	-0.5	0.4	-0.4

For corresponding atoms, four individual shifts (or shieldings) are averaged

Then individual shifts are expressed as deviations from this average

		8S,9S	8S,9R	8R,9R	8R,9S	average	8S,9S	8S,9R	8R,9R	8R,9S
C-6	yellow	144.8	144.6	136.3	136.3	140.5	-4.3	-4.1	4.2	4.2
C-7	blue	157.3	160.0	158.2	160.7	159.0	1.8	-1.0	0.8	-1.6
C-8	red	126.4	127.1	126.2	126.8	126.6	0.2	-0.4	0.4	-0.2

GIAO calculation

Negative sign of deviation is used for shieldings

Part B. Processing

24 differently ordered non-repeating assignments of four configurations to four compounds, corresponding to permutations of experimental and DFT data

№1	№2	. . .	№24
**1a** = *SS*	**1a** = *RS*		**1a** = *RS*
**1b** = *SR*	**1b** = *SR*		**1b** = *RR*
**1c** = *RR*	**1c** = *RR*		**1c** = *SR*
**1d** = *RS*	**1d** = *SS*		**1d** = *SS*

And corresponding data are set together for all atoms and all isomers

№1:

		experiment	DFT	
1a	C-6	-3.9	-4.3	8S,9S
1a	C-7	1.7	1.8	8S,9S
1a	C-8	0.4	0.2	8S,9S
1b	C-6	-3.6	-4.1	8S,9R
1b	C-7	-0.7	-1.0	8S,9R
1b	C-8	-0.5	-0.4	8S,9R
1c	C-6	4.3	4.2	8R,9R
1c	C-7	0.3	0.8	8R,9R
1c	C-8	0.4	0.4	8R,9R
1d	C-6	3.2	4.2	8R,9S
1d	C-7	-1.2	-1.6	8R,9S
1d	C-8	-0.4	-0.2	8R,9S

№2:

		experiment	DFT	
1a	C-6	-3.9	4.2	8R,9S
1a	C-7	1.7	-1.6	8R,9S
1a	C-8	0.4	-0.2	8R,9S
1b	C-6	-3.6	-4.1	8S,9R
1b	C-7	-0.7	-1.0	8S,9R
1b	C-8	-0.5	-0.4	8S,9R
1c	C-6	4.3	4.2	8R,9R
1c	C-7	0.3	0.8	8R,9R
1c	C-8	0.4	0.4	8R,9R
1d	C-6	3.2	-4.3	8S,9S
1d	C-7	-1.2	1.8	8S,9S
1d	C-8	-0.4	0.2	8S,9S

. . .

№24:

		experiment	DFT	
1a	C-6	-3.9	4.2	8R,9S
1a	C-7	1.7	-1.6	8R,9S
1a	C-8	0.4	-0.2	8R,9S
1b	C-6	-3.6	4.2	8R,9R
1b	C-7	-0.7	0.8	8R,9R
1b	C-8	-0.5	0.4	8R,9R
1c	C-6	4.3	-4.1	8S,9R
1c	C-7	0.3	-1.0	8S,9R
1c	C-8	0.4	-0.4	8S,9R
1d	C-6	3.2	-4.3	8S,9S
1d	C-7	-1.2	1.8	8S,9S
1d	C-8	-0.4	0.2	8S,9S

Selected comparison measure (cf. Table 1 in main text) is calculated to rank all the 24 permutations. In the example below: aggregate overlap

1. IF signs of Δ exp and Δ DFT match
   add lower value from |Δ exp| and |Δ DFT|
2. Repeat for next item

Overlap score: **20.0**	Overlap score: **9.6**	Overlap score: **0.0**

Finally the permutations are sorted according to their scores, the highest ranking permutation reflects the assignment predicted by computation

S3. Peripheral discussion

Alternative number of stereocenters (N) and diastereomers

N =1: For one varying stereocenter there are can be two diastereomers. The number of permutations (P_2 = 2! = 2) reduces the approach to the method by Goodman and Smith of comparing two isomers (CP3), and offers no advantage.

N = 3: For three varying stereocenters and eight possible diastereomers the number of permutations increases significantly (P_8 = 8! = 40320). The method could easily be applied by using the algorithm run by a computer, however both experimental and DFT computed data have to be obtained with very high precision and accuracy which is often unattainable for some compounds.

N = 4: For four varying centers and sixteen diastereomers the number of permutation becomes very large (P_{16} = 16! = 2 × 10^{13}) to the point of unfeasibility.

Alternative definition of midpoint.

Referencing the data instead of averages of chemical shifts and isotropic shieldings can be done using alternatively the corresponding median values. This approach was considered, because the midpoint is unaffected by the extreme values. However, for the studied compounds **1**–**7** no advantage of using the median was noted, the correct permutations received slightly worse scores, and separation between two highest rating permutations did not improve. In case of assignments of four possible configurations to three compounds the application of the median had a noticeably lower success ratio.

S4. Supporting Tables

Table S1. Percentage of correctly identified configuration by highest ranking permutation for sets of three diastereomers of compounds **1**–**7**. This is an expanded version of Table 4 from the main text

	^{13}C NMR data							^{1}H NMR data					
	**1**	**2**	**3**	**4**	**5**	**6**	**7**	**1**	**3**	**4**	**5**	**6**	**7**
CP1	75%	100%	100%	100%	100%	100%	100%	50%	0%	100%	75%	25%	25%
CP2	75%	75%	75%	100%	100%	50%	75%	75%	50%	100%	100%	50%	25%
CP3	75%	100%	100%	100%	100%	75%	75%	75%	25%	100%	100%	25%	25%
OL[a]	100%	100%	100%	100%	100%	50%	75%	100%	25%	100%	75%	50%	50%
RMS	100%	100%	100%	100%	100%	100%	100%	100%	25%	100%	100%	50%	25%
R[b]	100%	100%	100%	100%	100%	100%	100%	100%	25%	100%	100%	25%	25%
MAE	100%	100%	100%	100%	100%	75%	100%	100%	25%	100%	100%	50%	25%
[c]	88%	97%	97%	100%	100%	81%	91%	81%	22%	100%	91%	38%	28%

[a]Aggregate overlap, [b]Pearson correlation coefficient, [c]Average for compound tetrad. Cases when some of the measures did not point to the correct assignment were highlighted in yellow or orange. Each entry corresponds to four tests, where one experimental data was taken out.

S4.1. Tables of experimental NMR shifts and GIAO shieldings

Table S2. Assignment of ^{13}C and ^{1}H NMR signals for tetrad **1**, and calculated isotropic shieldings at the GIAO/PW1PW91/6-311+G(2d,p) level of theory

Atom label	Experimental chemical shift δ (ppm)				DFT isotropic shielding, σ (ppm)			
	**1a**	**1b**	**1c**	**1d**	8S,9S	8S,9R	8R,9R	8R,9S
				^{13}C				
C-2	56.21	55.84	47.22	49.25	127.66	128.15	137.03	136.81
C-3	39.26	39.46	39.01	39.76	141.24	140.86	140.61	140.65
C-4	27.79	27.21	27.61	27.52	153.46	154.39	153.55	154.31
C-5	27.77	27.74	26.47	26.43	156.78	156.55	158.32	158.15
C-6	41.19	41.47	49.41	48.27	144.78	144.59	136.28	136.32
C-7	27.65	25.20	26.19	24.70	157.25	160.04	158.20	160.66
C-8	58.21	57.39	58.28	57.48	126.40	127.07	126.25	126.85
C-9	60.67	61.39	59.60	60.83	122.83	122.15	123.95	122.51
C-10	141.44	141.67	140.24	140.02	34.99	35.10	37.04	36.77
C-11	114.97	114.91	115.02	115.30	68.84	69.08	68.83	68.65
C-2'	147.46	147.60	147.38	147.59	35.11	34.91	35.02	35.18
C-3'	119.45	119.93	119.52	119.69	63.26	61.57	62.89	61.97
C-4'	139.30	139.64	139.72	140.06	41.57	40.89	41.19	40.63
C-5'	100.89	100.41	100.50	100.48	83.58	83.51	83.35	84.27
C-6'	158.83	158.55	158.72	158.54	22.43	22.89	22.52	22.89
C-7'	122.53	122.20	122.50	122.21	59.43	60.34	59.69	60.31
C-8'	134.04	132.07	132.08	132.08	49.91	49.65	49.67	49.80
C-9'	128.33	128.00	128.12	128.07	53.23	54.53	53.56	54.10
C-10'	145.20	145.13	145.03	145.12	37.09	36.93	36.87	37.21
OCH_3	55.96	56.02	55.77	55.85	131.09	131.28	131.05	131.35
C-4''	139.26	134.58	133.81	134.44	48.66	48.10	48.61	48.18
C-5''	122.12	122.25	122.50	122.46	59.92	58.51	59.71	58.42
				^{1}H				
H-2a	2.778	2.899	3.073	2.715	29.263	29.042	28.641	29.141
H-2s	3.214	3.168	2.990	2.891	28.639	28.604	29.012	29.119
H-3	2.340	2.324	2.298	2.288	29.459	29.484	29.493	29.539
H-4	1.787	1.870	1.758	1.807	30.244	30.178	30.223	30.166
H-5n	1.627	1.564	1.642	1.651	30.245	30.314	30.223	30.232
H-5x	1.627	1.771	1.642	1.651	30.218	30.067	30.185	30.159
H-6n	2.751	2.653	2.902	2.885	29.235	29.299	28.985	28.987
H-6x	3.462	2.899	2.984	3.070	28.240	28.892	28.951	28.778
H-7n	1.937	1.382	1.436	1.685	29.726	30.187	30.413	29.873

H-7x	0.944	1.568	1.292	1.182	30.895	30.305	30.170	30.607
H-8	3.968	3.877	3.940	3.884	27.692	27.701	27.624	27.760
H-9	6.505	6.417	6.506	6.445	24.892	24.954	24.873	24.891
H-10	5.914	5.883	5.850	6.006	25.195	25.243	25.199	24.998
H-11c	5.090	5.075	5.115	5.075	26.376	26.365	26.384	26.370
H-11t	5.094	5.050	5.085	5.121	26.384	26.458	26.514	26.410
H-2'	8.808	8.833	8.779	8.821	22.489	22.504	22.536	22.538
H-3'	7.496	7.701	7.528	7.628	23.683	23.686	23.629	23.683
H-5'	7.505	7.389	7.470	7.332	23.765	23.980	23.671	23.962
H-7'	7.360	7.316	7.382	7.306	23.958	24.012	23.937	23.992
H-8'	8.012	7.985	8.021	7.976	23.253	23.323	23.268	23.275
OCH_3	3.936	3.909	3.953	3.885	27.772	27.804	27.731	27.795
H-4"	7.594	7.602	7.622	7.586	23.990	23.918	23.946	23.917
H-5"	7.509	7.308	7.560	7.318	24.152	24.118	24.091	24.123

Table S3. Assignment of ^{13}C NMR signals for tetrad **2**, and calculated isotropic shieldings at the GIAO/B3LYP/6-31G(d,p) level of theory

Atom label	**Experimental chemical shift δ (ppm)**				**DFT isotropic shielding, σ (ppm)**			
	**2a**	**2b**	**2c**	**2d**	8S,9S	8S,9R	8R,9R	8R,9S
^{13}C								
C-2	56.55	56.18	47.55	47.63	133.77	134.05	142.43	142.92
C-3	39.58	39.76	39.78	39.80	146.46	146.57	145.87	146.04
C-4	28.08	28.02	28.02	28.15	159.43	159.72	159.66	159.59
C-5	28.08	28.14	26.66	26.58	160.80	161.38	162.63	162.41
C-6	40.92	41.03	49.58	49.33	149.18	149.82	141.03	141.25
C-7	28.81	27.86	27.74	27.15	160.63	161.92	161.55	162.77
C-8	59.48	58.26	58.99	58.08	129.30	130.49	129.34	131.29
C-9	49.47	50.18	48.11	49.33	139.57	138.10	140.60	139.30
C-10	142.03	142.12	140.90	140.83	54.30	54.70	56.27	56.11
C-11	114.28	114.71	114.44	114.67	82.15	82.23	81.77	81.71
OCH_3	55.48	55.47	55.45	55.54	138.74	138.73	138.74	139.05
C-2'	147.61	147.81	147.55	147.82	50.31	50.45	50.32	50.40
C-3'	119.75	119.70	120.06	119.46	75.03	73.39	75.34	74.31
C-4'	146.76	146.93	147.16	147.20	48.77	49.90	49.04	49.22
C-5'	102.09	102.01	101.71	102.17	93.68	93.91	93.84	93.69
C-6'	157.72	157.45	157.83	157.53	39.55	40.06	39.49	40.07
C-7'	120.92	120.53	121.28	121.47	74.91	75.68	74.88	75.63
C-8'	131.91	131.87	131.88	131.83	63.90	63.98	63.90	64.00
C-9'	128.79	128.44	128.79	128.67	65.90	66.31	65.79	66.17
C-10'	144.77	144.86	144.80	144.89	51.29	51.41	51.31	51.32
C-*ipso*	142.16	140.71	142.32	140.73	52.68	54.14	52.59	54.25

C-*ortho*	127.88	128.96	127.97	128.95	68.47	67.38	68.41	67.69
C-*meta*	128.47	128.61	128.48	128.70	69.14	68.57	69.19	68.56
C-*para*	126.68	126.87	126.60	127.01	70.95	69.01	70.97	70.28

Table S4. Assignment of ^{13}C and ^{1}H NMR signals for tetrad **3**, and calculated isotropic shieldings at the GIAO/PW1PW91/6-311+G(2d,p) level of theory

Atom label	**Experimental chemical shift δ (ppm)**				**DFT isotropic shielding, σ (ppm)**			
	**3a**	**3b**	**3c**	**3d**	8S,9S	8S,9R	8R,9R	8R,9S
				^{13}C				
C-2	57.5	57.6	48.8	49.5	127.22	126.87	135.10	134.91
C-3	39.8	39.9	39.6	39.8	140.73	140.36	141.06	140.25
C-4	28.4	28.3	29.4	29.1	153.18	153.25	152.56	152.42
C-5	27.5	27.8	26.4	26.2	157.63	157.50	157.78	158.64
C-6	43.1	43.6	50.8	50.8	143.02	141.56	134.35	135.29
C-7	25.3	25.1	24.4	23.8	158.99	160.24	159.73	161.35
C-8	62.8	61.1	63.1	61.4	120.68	124.68	119.89	124.50
C-9	78.9	79.7	78.4	79.3	103.37	101.10	103.37	99.96
C-10	141.9	142.2	139.1	139.7	35.07	34.37	37.43	35.64
C-11	115	114.7	114.6	114.4	68.39	69.79	68.66	70.09
C-2'	147.1	147.4	144	147.4	35.65	35.06	35.56	35.03
C-3'	117.6	120.4	117.5	120.5	66.23	61.51	65.70	61.37
C-4'	150	149.4	149.8	149.7	28.63	29.16	28.85	29.57
C-5'	106.3	104.8	106.3	105.1	77.09	80.02	77.48	80.31
C-6'	156.8	156.5	156.7	156.4	24.76	24.45	24.47	24.74
C-7'	121.5	121.3	121.5	121.1	61.36	61.43	61.36	61.57
C-8'	131.2	131.5	131	131.4	50.63	49.76	50.65	49.66
C-9'	128.8	126.9	127.5	127.1	54.55	55.93	54.67	56.29
C-10'	146.1	145.3	146.8	145.3	35.70	36.41	35.87	36.81
OCH3	55.4	55.3	55.3	55.3	131.85	132.06	131.88	132.04
C-*ipso*	143.9	145.7	146.1	145.5	35.40	31.84	35.67	32.62
C-*para*	127	127.5	127	127.7	56.31	54.17	55.67	54.77
C-*ortho*	127.7	126.9	127.5	127.2	53.71	53.22	54.05	53.23
C-*meta*	127.6	128.5	127.7	128.6	55.58	53.81	55.63	53.76
				^{1}H				
H-2a	3.102	3.16	2.624	2.719	29.058	28.897	29.387	29.009
H-2s	3.205	3.194	2.716	2.719	28.643	28.548	29.139	27.905
H-3	2.44	2.362	2.134	2.103	29.499	29.519	29.687	29.577
H-4	1.987	1.843	1.855	1.715	30.107	30.381	30.071	30.343
H-5n	1.259	1.382	1.692	1.62	30.812	30.419	30.207	30.449
H-5x	1.386	1.431	1.874	1.741	30.566	30.178	29.954	30.345
H-6n	2.635	2.623	2.883	2.889	29.349	29.072	28.884	28.907

H-6x	2.635	3.154	3.129	3.242	28.834	27.640	28.740	28.598
H-7n	1.751	1.451	1.828	1.742	30.035	31.012	29.718	31.460
H-7x	2.093	1.612	1.984	1.742	29.308	30.361	29.630	29.904
H-8	3.833	4.051	3.719	3.966	27.839	27.616	27.842	27.684
H-10	6.072	5.946	5.228	5.446	25.018	25.666	26.327	25.003
H-11c	5.218	5.134	4.537	4.64	26.327	26.520	26.582	26.460
H-11t	5.24	5.099	4.758	4.777	26.366	26.697	26.796	26.500
H-2'	8.809	8.801	8.795	8.834	22.606	22.483	22.508	22.487
H-3'	7.395	8.037	7.488	8.096	23.923	23.231	23.783	23.159
H-5'	7.589	6.839	7.623	6.885	23.667	24.638	23.621	24.585
H-7'	7.183	7.184	7.186	7.188	24.128	24.083	24.145	24.108
H-8'	7.902	7.94	7.901	7.951	23.392	23.309	23.415	23.315
para	7.205	7.252	7.214	7.266	24.139	24.071	24.128	24.061
ortho	7.434	7.483	7.449	7.503	23.842	23.127	23.823	23.189
meta	7.266	7.31	7.274	7.32	24.087	24.038	24.087	23.963
OCH_3	3.639	3.484	3.645	3.465	28.006	28.256	28.006	28.220

Table S5. Assignment of ^{13}C and ^{1}H NMR signals for tetrad **4**, and calculated isotropic shieldings at the GIAO/B3LYP/6-31G(d,p) level of theory

Atom label	**Experimental chemical shift δ (ppm)**				**DFT isotropic shielding, σ (ppm)**			
	**4a**	**4b**	**4c**	**4d**	8S,9S	8S,9R	8R,9R	8R,9S
^{13}C								
C-2	56.1	57.4	49	49.1	134.26	133.52	141.51	141.21
C-3	39.4	39.8	40.1	39.7	146.62	145.96	146.27	146.02
C-4	27.7	27.8	28.3	28.3	159.41	159.38	158.97	159.14
C-5	27.4	27.8	26.6	26.4	161.58	161.31	162.58	162.89
C-6	42.4	42.7	49.4	50.3	148.12	147.70	141.24	140.41
C-7	20.2	24	20.1	20.9	168.32	163.98	168.73	165.13
C-8	57.5	56.1	57.6	55.5	131.30	134.72	131.41	134.87
C-9	59.1	62.8	59.2	63.2	130.45	125.81	130.07	125.91
C-10	142.1	141.8	140.7	140.3	54.40	54.69	54.58	55.85
C-11	114.5	114.4	114.1	114.5	82.06	82.09	81.82	81.84
C-2'	147.7	147.4	147.7	147.4	50.08	49.89	50.07	49.91
C-3'	119.9	120.2	120.3	120	75.61	75.32	75.49	75.38
C-4'	144.1	143.7	144.6	143.7	49.17	51.16	49.18	51.12
C-5'	102.2	102.1	102.4	102.0	94.20	93.99	94.30	93.94
C-6'	157.5	158	157.5	157.9	39.58	39.20	39.63	39.31
C-7'	120.5	121.8	120.4	121.7	74.49	74.46	74.82	74.47
C-8'	131.9	131.8	132	131.8	63.65	63.69	63.69	63.71
C-9'	126.7	126.8	126.7	126.8	68.07	68.09	68.07	68.09
C-10'	144.7	144.5	144.1	144.4	51.84	51.90	51.87	51.88

C-1"	47.2	51.4	47.4	51.8	143.81	139.33	143.55	139.15
OCH_3	55.5	55.6	55.6	55.5	138.87	138.59	138.82	138.62
^1H								
H-2s	2.43	2.66	2.98	2.97	29.57	29.15	28.62	28.4
H-2a	2.86	3.04	2.75	2.76	29.08	28.84	29.13	29.16
H-3	2.16	2.2	2.13	2.13	29.75	29.74	29.73	29.75
H-4	1.79	1.76	1.72	1.68	30.04	30.09	30.1	30.16
H-5n	1.39	1.43	1.47	1.44	30.46	30.51	30.43	30.45
H-5x	1.64	1.62	1.47	1.42	30.22	30.22	30.37	30.45
H-6n	2.51	2.59	2.56	2.7	29.4	29.41	29.38	29.14
H-6x	3.22	3.18	2.56	2.84	28.44	28.22	29.36	28.91
H-7n	1.64	1.54	1.62	1.81	30.09	30.35	30.03	29.7
H-7x	1.24	1.48	1.29	1.15	30.48	30.13	30.57	30.8
H-8	3.67	3.4	3.56	3.29	27.92	28.23	27.97	28.31
H-10	5.84	5.65	6.01	5.93	25.44	25.73	25.08	25.2
H-11c	4.97	4.88	5	5.01	26.62	26.69	26.56	26.58
H-11t	4.98	4.85	5.02	5.01	26.72	26.85	26.63	26.66
H-1"p	3.01	3.61	3.03	3.63	28.71	27.85	28.68	27.79
H-1"f	2.58	2.8	2.62	2.75	29.17	29.02	29.14	29
H-2'	8.7	8.68	8.7	8.67	22.93	22.88	22.92	22.88
H-3'	7.44	7.35	7.44	7.37	24.12	24.13	24.09	24.1
H-5'	7.1	7.26	7.12	7.22	24.61	24.51	24.61	24.54
H-7'	7.33	7.33	7.33	7.31	24.26	24.23	24.27	24.24
H-8'	8	7.99	8	7.97	23.63	23.62	23.64	23.62
OCH_3	3.89	3.88	3.88	3.87	27.82	27.79	27.83	27.79

S4.2. Tables with all permutations and their scores

Table S6a. Complete list of permutations with scores for comparison of experimental and calculated ^{13}C NMR data for compound tetrad **1**

**Permutation**				**^{13}C data score**						
**1a**	**1b**	**1c**	**1d**	**CP1**	**CP2**	**CP3**	**overlap**	**RMS**	**correl**	**MAE**
8S,9S	8S,9R	8R,9R	8R,9S	0.951	0.740	0.739	48.05	0.640	0.892	0.378
8S,9S	8S,9R	8R,9S	8R,9R	0.905	0.700	0.694	43.59	0.758	0.849	0.479
8S,9R	8S,9S	8R,9R	8R,9S	0.902	0.720	0.705	42.51	0.764	0.846	0.503
8S,9R	8S,9S	8R,9S	8R,9R	0.855	0.680	0.660	38.05	0.864	0.802	0.605
8S,9S	8R,9S	8R,9R	8S,9R	0.180	0.237	0.101	30.47	1.766	0.169	0.777
8S,9S	8R,9R	8R,9S	8S,9R	0.120	0.210	0.053	26.60	1.824	0.113	0.865
8S,9R	8R,9S	8R,9R	8S,9S	0.098	0.226	0.059	25.45	1.846	0.092	0.891
8R,9S	8S,9S	8R,9R	8S,9R	0.085	0.124	-0.001	24.53	1.857	0.080	0.912
8R,9R	8S,9S	8R,9S	8S,9R	0.077	0.133	-0.007	25.41	1.866	0.072	0.892

8R,9S	8S,9R	8R,9R	8S,9S	0.052	0.134	-0.009	25.04	1.888	0.049	0.900
8R,9R	8S,9R	8R,9S	8S,9S	0.044	0.143	-0.015	25.93	1.897	0.041	0.880
8S,9R	8R,9R	8R,9S	8S,9S	0.038	0.200	0.011	21.57	1.902	0.035	0.979
8S,9S	8R,9S	8S,9R	8R,9R	-0.036	-0.134	-0.199	24.14	1.969	-0.034	0.921
8S,9S	8R,9R	8S,9R	8R,9S	-0.049	-0.120	-0.201	24.72	1.981	-0.046	0.908
8S,9R	8R,9S	8S,9S	8R,9R	-0.080	-0.143	-0.209	22.50	2.008	-0.075	0.958
8R,9R	8S,9R	8S,9S	8R,9S	-0.087	-0.187	-0.238	27.44	2.014	-0.082	0.846
8R,9R	8S,9S	8S,9R	8R,9S	-0.093	-0.198	-0.262	23.54	2.019	-0.087	0.934
8S,9R	8R,9R	8S,9S	8R,9S	-0.093	-0.130	-0.212	23.08	2.019	-0.087	0.945
8R,9S	8S,9R	8S,9S	8R,9R	-0.125	-0.236	-0.277	22.09	2.047	-0.117	0.967
8R,9S	8S,9S	8S,9R	8R,9R	-0.130	-0.246	-0.301	18.20	2.051	-0.122	1.056
8R,9R	8R,9S	8S,9S	8S,9R	-0.859	-0.691	-0.876	9.86	2.601	-0.806	1.245
8R,9R	8R,9S	8S,9R	8S,9S	-0.897	-0.691	-0.908	6.48	2.627	-0.841	1.322
8R,9S	8R,9R	8S,9S	8S,9R	-0.910	-0.726	-0.918	5.10	2.635	-0.854	1.354
8R,9S	8R,9R	8S,9R	8S,9S	-0.948	-0.727	-0.949	1.72	2.661	-0.889	1.430

Table S6b. Complete list of permutations with scores for comparison of experimental and calculated ^{1}H NMR data for compound tetrad **1**

**Permutation**				**^{1}H data score**						
**1a**	**1b**	**1c**	**1d**	**CP1**	**CP2**	**CP3**	**overlap**	**RMS**	**correl**	**MAE**
8S,9S	8S,9R	8R,9R	8R,9S	1.114	0.642	0.641	4.61	0.070	0.850	0.049
8S,9S	8R,9R	8S,9R	8R,9S	0.860	0.560	0.497	3.73	0.100	0.656	0.068
8S,9S	8R,9R	8R,9S	8S,9R	0.605	0.396	0.298	3.05	0.123	0.462	0.083
8S,9S	8S,9R	8R,9S	8R,9R	0.592	0.470	0.329	3.40	0.124	0.452	0.076
8S,9S	8R,9S	8R,9R	8S,9R	0.556	0.268	0.176	3.07	0.127	0.425	0.083
8R,9S	8S,9R	8R,9R	8S,9S	0.511	0.285	0.207	3.04	0.131	0.390	0.083
8S,9S	8R,9S	8S,9R	8R,9R	0.289	0.261	0.064	2.54	0.147	0.221	0.094
8R,9S	8R,9R	8S,9R	8S,9S	0.257	0.204	0.064	2.16	0.149	0.196	0.103
8S,9R	8R,9R	8R,9S	8S,9S	0.047	-0.089	-0.177	1.92	0.163	0.036	0.108
8S,9R	8R,9S	8R,9R	8S,9S	-0.002	-0.217	-0.299	1.93	0.166	-0.002	0.108
8S,9R	8R,9R	8S,9S	8R,9S	-0.013	-0.022	-0.209	2.27	0.166	-0.010	0.100
8R,9S	8R,9R	8S,9S	8S,9R	-0.058	0.107	-0.168	1.84	0.169	-0.044	0.110
8S,9R	8S,9S	8R,9R	8R,9S	-0.060	-0.162	-0.365	2.51	0.169	-0.046	0.095
8R,9S	8S,9R	8S,9S	8R,9R	-0.071	0.180	-0.137	2.19	0.170	-0.054	0.102
8R,9S	8S,9S	8R,9R	8S,9R	-0.105	-0.034	-0.324	2.07	0.172	-0.080	0.105
8R,9R	8S,9R	8R,9S	8S,9S	-0.171	-0.195	-0.333	2.20	0.176	-0.131	0.102
8R,9R	8S,9R	8S,9S	8R,9S	-0.231	-0.128	-0.365	2.56	0.179	-0.177	0.094
8R,9S	8S,9S	8S,9R	8R,9R	-0.372	-0.041	-0.436	1.54	0.187	-0.284	0.116
8R,9R	8R,9S	8S,9R	8S,9S	-0.474	-0.404	-0.598	1.33	0.192	-0.362	0.121
8R,9R	8S,9S	8S,9R	8R,9S	-0.532	-0.350	-0.665	1.91	0.195	-0.406	0.108
8S,9R	8S,9S	8R,9S	8R,9R	-0.582	-0.334	-0.677	1.30	0.198	-0.444	0.121
8S,9R	8R,9S	8S,9S	8R,9R	-0.584	-0.322	-0.643	1.08	0.198	-0.446	0.126

8R,9R	8S,9S	8R,9S	8S,9R	-0.787	-0.514	-0.864	1.23	0.208	-0.601	0.123
8R,9R	8R,9S	8S,9S	8S,9R	-0.789	-0.501	-0.830	1.01	0.208	-0.602	0.128

Data is sorted according to Pearson correlation coefficient (correl). Permutation corresponding to correct assignment is highlighted in green, scores corresponding to best match of the data (highest CP1, CP2, CP3, aggregate overlap, correlation; and lowest RMS deviation and MAE) are highlighted in blue.

Table S7. Complete list of permutations with scores for comparison of experimental and calculated ^{13}C NMR data for compound tetrad **2**

**Permutation**				**^{13}C data score**						
**2a**	**2b**	**2c**	**2d**	**CP1**	**CP2**	**CP3**	**overlap**	**RMS**	**correl**	**MAE**
8S,9S	8S,9R	8R,9R	8R,9S	0.995	0.938	0.935	52.82	0.330	0.969	0.226
8S,9S	8S,9R	8R,9S	8R,9R	0.950	0.901	0.894	47.55	0.512	0.925	0.336
8S,9R	8S,9S	8R,9R	8R,9S	0.948	0.891	0.884	46.58	0.519	0.923	0.356
8S,9R	8S,9S	8R,9S	8R,9R	0.903	0.854	0.843	41.31	0.650	0.879	0.466
8S,9S	8R,9S	8R,9R	8S,9R	0.057	0.081	0.035	31.99	1.813	0.055	0.660
8R,9R	8S,9R	8S,9S	8R,9S	0.037	0.003	-0.014	31.11	1.831	0.036	0.678
8S,9R	8R,9S	8R,9R	8S,9S	0.028	0.046	0.002	27.97	1.839	0.027	0.744
8S,9S	8R,9R	8R,9S	8S,9R	0.018	0.031	-0.007	26.37	1.849	0.017	0.777
8S,9R	8R,9R	8S,9S	8R,9S	0.015	0.001	-0.025	26.53	1.852	0.014	0.774
8R,9R	8S,9R	8R,9S	8S,9S	0.011	-0.001	-0.028	26.93	1.855	0.011	0.766
8S,9R	8R,9S	8S,9S	8R,9R	0.009	0.014	-0.025	26.88	1.857	0.009	0.767
8S,9S	8R,9R	8S,9R	8R,9S	0.001	-0.005	-0.032	27.40	1.865	0.001	0.756
8R,9S	8S,9R	8R,9R	8S,9S	-0.002	-0.002	-0.033	28.18	1.867	-0.002	0.739
8S,9S	8R,9S	8S,9R	8R,9R	-0.005	0.007	-0.032	27.75	1.870	-0.005	0.748
8R,9R	8S,9S	8R,9S	8S,9R	-0.007	-0.014	-0.046	24.72	1.872	-0.007	0.812
8S,9R	8R,9R	8R,9S	8S,9S	-0.011	-0.003	-0.040	22.35	1.875	-0.011	0.861
8R,9S	8S,9S	8R,9R	8S,9R	-0.021	-0.015	-0.051	25.97	1.884	-0.020	0.786
8R,9S	8S,9R	8S,9S	8R,9R	-0.021	-0.034	-0.060	27.09	1.884	-0.021	0.762
8R,9R	8S,9S	8S,9R	8R,9S	-0.025	-0.051	-0.072	25.74	1.887	-0.024	0.790
8R,9S	8S,9S	8S,9R	8R,9R	-0.083	-0.089	-0.118	21.72	1.939	-0.081	0.874
8R,9R	8R,9S	8S,9S	8S,9R	-0.901	-0.854	-0.914	10.28	2.556	-0.878	1.112
8R,9R	8R,9S	8S,9R	8S,9S	-0.944	-0.895	-0.954	7.13	2.584	-0.919	1.178
8R,9S	8R,9R	8S,9S	8S,9R	-0.954	-0.904	-0.961	5.92	2.590	-0.929	1.203
8R,9S	8R,9R	8S,9R	8S,9S	-0.997	-0.945	-1.001	2.76	2.618	-0.971	1.269

Data is sorted according to Pearson correlation coefficient (correl), permutation corresponding to correct assignment is highlighted in green, scores corresponding to best match of the data (highest CP1, CP2, CP3, aggregate overlap, correlation; and lowest RMS deviation and MAE) are highlighted in blue.

Table S8a. Complete list of permutations with scores for comparison of experimental and calculated ^{13}C NMR data for compound tetrad **3**

**Permutation**	**^{13}C data score**

3a	3b	3c	3d	CP1	CP2	CP3	overlap	RMS	correl	MAE
8S,9S	8S,9R	8R,9R	8R,9S	0.986	0.806	0.797	62.86	0.703	0.881	0.522
8S,9S	8S,9R	8R,9S	8R,9R	0.780	0.707	0.641	50.74	1.104	0.697	0.775
8S,9R	8S,9S	8R,9R	8R,9S	0.747	0.697	0.612	49.75	1.157	0.667	0.795
8S,9R	8S,9S	8R,9S	8R,9R	0.541	0.599	0.456	37.63	1.437	0.483	1.048
8S,9S	8R,9S	8R,9R	8S,9R	0.295	0.135	0.108	44.98	1.711	0.264	0.895
8R,9R	8S,9R	8S,9S	8R,9S	0.141	0.055	-0.001	44.09	1.863	0.126	0.913
8S,9S	8R,9S	8S,9R	8R,9R	0.072	-0.015	-0.076	34.25	1.928	0.064	1.118
8R,9S	8S,9S	8R,9R	8S,9R	0.038	0.015	-0.086	30.96	1.959	0.034	1.187
8S,9R	8R,9S	8R,9R	8S,9S	0.029	0.046	-0.070	32.95	1.967	0.025	1.145
8S,9S	8R,9R	8R,9S	8S,9R	0.015	0.039	-0.083	32.68	1.979	0.014	1.151
8R,9S	8S,9R	8R,9R	8S,9S	0.010	0.035	-0.079	32.03	1.983	0.009	1.165
8S,9R	8R,9S	8S,9S	8R,9R	0.001	-0.010	-0.116	32.09	1.992	0.001	1.163
8S,9S	8R,9R	8S,9R	8R,9S	-0.002	-0.013	-0.111	34.07	1.994	-0.002	1.122
8R,9R	8S,9S	8R,9S	8S,9R	-0.009	-0.007	-0.119	31.76	2.000	-0.008	1.170
8R,9S	8S,9R	8S,9S	8R,9R	-0.017	-0.021	-0.125	31.17	2.008	-0.016	1.183
8R,9R	8S,9S	8S,9R	8R,9S	-0.027	-0.059	-0.146	33.15	2.016	-0.024	1.141
8R,9R	8S,9R	8R,9S	8S,9S	-0.036	0.012	-0.111	32.83	2.024	-0.033	1.148
8S,9R	8R,9R	8S,9S	8R,9S	-0.074	-0.008	-0.151	31.91	2.056	-0.066	1.167
8R,9S	8S,9S	8S,9R	8R,9R	-0.185	-0.135	-0.271	20.23	2.150	-0.166	1.410
8S,9R	8R,9R	8R,9S	8S,9S	-0.251	-0.050	-0.261	20.64	2.204	-0.225	1.402
8R,9R	8R,9S	8S,9S	8S,9R	-0.549	-0.616	-0.690	26.22	2.430	-0.491	1.286
8R,9R	8R,9S	8S,9R	8S,9S	-0.745	-0.710	-0.828	16.35	2.568	-0.665	1.491
8R,9S	8R,9R	8S,9S	8S,9R	-0.782	-0.689	-0.849	13.11	2.594	-0.699	1.559
8R,9S	8R,9R	8S,9R	8S,9S	-0.978	-0.783	-0.987	3.24	2.723	-0.873	1.764

Table S8b. Complete list of permutations with scores for comparison of experimental and calculated ^{1}H NMR data for compound tetrad **3**

Permutation				^{1}H data score						
**3a**	**3b**	**3c**	**3d**	**CP1**	**CP2**	**CP3**	**overlap**	**RMS**	**correl**	**MAE**
8S,9S	8R,9S	8R,9R	8S,9R	1.111	0.568	0.551	10.22	0.212	0.714	0.143
8S,9S	8S,9R	8R,9R	8R,9S	0.870	0.509	0.424	9.57	0.251	0.559	0.157
8S,9S	8R,9S	8S,9R	8R,9R	0.551	0.250	0.132	7.28	0.295	0.354	0.207
8R,9S	8S,9S	8R,9R	8S,9R	0.462	0.206	0.050	7.14	0.306	0.297	0.210
8R,9S	8S,9R	8R,9R	8S,9S	0.339	0.144	-0.030	6.18	0.321	0.218	0.231
8S,9S	8S,9R	8R,9S	8R,9R	0.319	0.130	-0.037	6.70	0.323	0.205	0.220
8R,9R	8R,9S	8S,9S	8S,9R	0.318	0.135	-0.056	6.65	0.323	0.204	0.221
8R,9S	8S,9R	8S,9S	8R,9R	0.235	-0.001	-0.136	5.68	0.332	0.151	0.242
8S,9R	8R,9S	8R,9R	8S,9S	0.124	0.056	-0.227	5.58	0.345	0.080	0.244
8R,9R	8S,9R	8S,9S	8R,9S	0.077	0.077	-0.183	5.99	0.350	0.049	0.235
8S,9R	8R,9S	8S,9S	8R,9R	0.020	-0.089	-0.333	5.09	0.356	0.013	0.255

8S,9R	8S,9S	8R,9R	8R,9S	0.006	0.059	-0.274	5.90	0.357	0.004	0.237
8R,9S	8S,9S	8S,9R	8R,9R	-0.099	-0.113	-0.369	4.20	0.368	-0.064	0.274
8S,9S	8R,9R	8R,9S	8S,9R	-0.102	0.019	-0.320	5.20	0.368	-0.065	0.252
8S,9S	8R,9R	8S,9R	8R,9S	-0.111	0.080	-0.277	5.12	0.369	-0.071	0.254
8R,9R	8R,9S	8S,9R	8S,9S	-0.139	-0.038	-0.369	4.20	0.372	-0.089	0.274
8R,9S	8R,9R	8S,9S	8S,9R	-0.186	-0.111	-0.419	4.18	0.377	-0.119	0.275
8R,9R	8S,9S	8R,9S	8S,9R	-0.248	-0.096	-0.458	4.59	0.383	-0.159	0.266
8R,9R	8S,9S	8S,9R	8R,9S	-0.257	-0.035	-0.416	4.51	0.384	-0.165	0.267
8R,9R	8S,9R	8R,9S	8S,9S	-0.371	-0.158	-0.538	3.63	0.395	-0.238	0.287
8S,9R	8S,9S	8R,9S	8R,9R	-0.545	-0.321	-0.735	3.03	0.411	-0.351	0.299
8S,9R	8R,9R	8S,9S	8R,9S	-0.642	-0.259	-0.743	2.93	0.420	-0.412	0.302
8R,9S	8R,9R	8S,9R	8S,9S	-0.643	-0.285	-0.732	1.73	0.420	-0.413	0.328
8S,9R	8R,9R	8R,9S	8S,9S	-1.089	-0.493	-1.098	0.56	0.458	-0.700	0.353

Data is sorted according to Pearson correlation coefficient (correl), permutation corresponding to correct assignment is highlighted in green, scores corresponding to best match of the data (highest CP1, CP2, CP3, aggregate overlap, correlation; and lowest RMS deviation and MAE) are highlighted in blue.

Table S9a. Complete list of permutations with scores for comparison of experimental and calculated ^{13}C NMR data for compound tetrad **4**

**Permutation**				**^{13}C data score**						
**4a**	**4b**	**4c**	**4d**	**CP1**	**CP2**	**CP3**	**overlap**	**RMS**	**correl**	**MAE**
8S,9S	8S,9R	8R,9R	8R,9S	1.000	0.915	0.912	61.46	0.411	0.960	0.275
8S,9S	8S,9R	8R,9S	8R,9R	0.705	0.645	0.613	48.20	1.161	0.677	0.590
8S,9R	8S,9S	8R,9R	8R,9S	0.643	0.624	0.567	45.98	1.263	0.618	0.643
8R,9R	8S,9R	8S,9S	8R,9S	0.397	0.361	0.314	45.71	1.606	0.381	0.650
8S,9R	8S,9S	8R,9S	8R,9R	0.349	0.353	0.268	32.72	1.665	0.335	0.959
8S,9S	8R,9S	8R,9R	8S,9R	0.243	0.194	0.154	42.24	1.787	0.234	0.732
8R,9S	8S,9R	8S,9S	8R,9R	0.069	0.074	0.006	31.96	1.972	0.067	0.977
8R,9R	8S,9R	8R,9S	8S,9S	0.056	0.046	-0.017	32.59	1.986	0.053	0.962
8R,9R	8S,9S	8S,9R	8R,9S	0.029	0.093	-0.010	31.74	2.013	0.028	0.982
8R,9S	8S,9R	8R,9R	8S,9S	0.023	0.029	-0.026	32.10	2.019	0.022	0.974
8S,9R	8R,9R	8S,9S	8R,9S	-0.001	-0.012	-0.077	30.27	2.042	-0.001	1.017
8S,9R	8R,9S	8S,9S	8R,9R	-0.002	-0.025	-0.099	29.30	2.043	-0.002	1.040
8R,9R	8S,9S	8R,9S	8S,9R	-0.009	0.019	-0.078	29.90	2.050	-0.009	1.026
8S,9S	8R,9R	8S,9R	8R,9S	-0.012	0.011	-0.056	31.78	2.053	-0.011	0.981
8S,9S	8R,9S	8S,9R	8R,9R	-0.013	-0.002	-0.078	30.81	2.054	-0.013	1.004
8R,9S	8S,9S	8R,9R	8S,9R	-0.042	0.002	-0.086	29.41	2.082	-0.040	1.038
8S,9R	8R,9S	8R,9R	8S,9S	-0.049	-0.069	-0.131	29.44	2.088	-0.047	1.037
8S,9S	8R,9R	8R,9S	8S,9R	-0.050	-0.063	-0.123	29.95	2.089	-0.048	1.025
8R,9S	8S,9S	8S,9R	8R,9R	-0.298	-0.195	-0.317	17.98	2.315	-0.286	1.310
8S,9R	8R,9R	8R,9S	8S,9S	-0.342	-0.327	-0.408	17.15	2.352	-0.328	1.330

8R,9R	8R,9S	8S,9S	8S,9R	-0.360	-0.360	-0.445	26.49	2.368	-0.346	1.107
8R,9R	8R,9S	8S,9R	8S,9S	-0.663	-0.600	-0.708	15.20	2.611	-0.636	1.376
8R,9S	8R,9R	8S,9S	8S,9R	-0.686	-0.635	-0.730	13.70	2.628	-0.659	1.412
8R,9S	8R,9R	8S,9R	8S,9S	-0.989	-0.875	-0.993	2.42	2.850	-0.950	1.680

Table S9b. Complete list of permutations with scores for comparison of experimental and calculated ^{1}H NMR data for compound tetrad **4**

**Permutation**				**^{1}H data score**						
**4a**	**4b**	**4c**	**4d**	**CP1**	**CP2**	**CP3**	**overlap**	**RMS**	**correl**	**MAE**
8S,9S	8S,9R	8R,9R	8R,9S	1.528	0.655	0.654	6.59	0.089	0.952	0.056
8S,9S	8S,9R	8R,9S	8R,9R	0.836	0.366	0.233	4.95	0.169	0.521	0.093
8S,9R	8S,9S	8R,9R	8R,9S	0.799	0.320	0.197	4.68	0.173	0.498	0.099
8R,9R	8S,9R	8S,9S	8R,9S	0.596	0.210	0.005	4.85	0.190	0.371	0.096
8S,9S	8R,9S	8R,9R	8S,9R	0.593	0.314	0.076	4.06	0.190	0.370	0.114
8R,9S	8S,9R	8R,9R	8S,9S	0.168	0.029	-0.309	3.57	0.221	0.105	0.125
8S,9R	8S,9S	8R,9S	8R,9R	0.107	0.031	-0.224	3.04	0.225	0.067	0.137
8R,9R	8S,9R	8R,9S	8S,9S	0.076	0.066	-0.299	3.49	0.227	0.047	0.127
8S,9R	8R,9S	8R,9R	8S,9S	0.067	0.010	-0.312	2.89	0.228	0.042	0.140
8S,9S	8R,9R	8S,9R	8R,9S	0.064	0.004	-0.356	3.33	0.228	0.040	0.130
8S,9S	8R,9R	8R,9S	8S,9R	0.006	0.118	-0.299	2.93	0.232	0.004	0.139
8S,9R	8R,9R	8S,9S	8R,9S	0.001	-0.042	-0.384	3.13	0.232	0.000	0.135
8R,9S	8S,9R	8S,9S	8R,9R	-0.003	-0.116	-0.426	3.29	0.232	-0.002	0.131
8R,9S	8S,9S	8R,9R	8S,9R	-0.035	-0.002	-0.377	2.82	0.234	-0.022	0.142
8S,9S	8R,9S	8S,9R	8R,9R	-0.042	-0.089	-0.402	2.81	0.235	-0.026	0.142
8R,9R	8S,9S	8S,9R	8R,9S	-0.070	-0.079	-0.424	3.13	0.237	-0.044	0.135
8S,9R	8R,9S	8S,9S	8R,9R	-0.104	-0.134	-0.429	2.62	0.239	-0.065	0.146
8R,9R	8S,9S	8R,9S	8S,9R	-0.127	0.035	-0.368	2.74	0.240	-0.079	0.144
8R,9R	8R,9S	8S,9S	8S,9R	-0.339	-0.130	-0.572	2.32	0.253	-0.211	0.153
8S,9R	8R,9R	8R,9S	8S,9S	-0.520	-0.186	-0.688	1.77	0.264	-0.324	0.166
8R,9S	8S,9S	8S,9R	8R,9R	-0.670	-0.405	-0.855	1.58	0.272	-0.417	0.170
8R,9R	8R,9S	8S,9R	8S,9S	-0.802	-0.389	-0.933	1.35	0.279	-0.500	0.175
8R,9S	8R,9R	8S,9S	8S,9R	-0.833	-0.364	-0.958	1.27	0.281	-0.519	0.177
8R,9S	8R,9R	8S,9R	8S,9S	-1.297	-0.622	-1.318	0.31	0.305	-0.808	0.199

Data is sorted according to Pearson correlation coefficient (correl), permutation corresponding to correct assignment is highlighted in green, scores corresponding to best match of the data (highest CP1, CP2, CP3, aggregate overlap, correlation; and lowest RMS deviation and MAE) are highlighted in blue.

Table S10a. Complete list of permutations with scores for comparison of experimental and calculated ^{13}C NMR data for compound tetrad **5**

**Permutation**	**^{13}C data score**

5a	5b	5c	5d	CP1	CP2	CP3	overlap	RMS	correl	MAE
1S,3R	1R,3R	1S,3S	1R,3S	1.059	0.780	0.769	30.38	0.732	0.920	0.569
1S,3R	1R,3S	1S,3S	1R,3R	0.720	0.553	0.538	21.96	1.515	0.626	1.037
1S,3R	1S,3S	1R,3R	1R,3S	0.610	0.554	0.446	20.80	1.693	0.531	1.101
1S,3R	1R,3R	1R,3S	1S,3S	0.489	0.377	0.287	20.30	1.870	0.425	1.129
1S,3R	1R,3S	1R,3R	1S,3S	0.425	0.378	0.297	17.38	1.958	0.369	1.291
1S,3R	1S,3S	1R,3S	1R,3R	0.336	0.327	0.205	15.30	2.072	0.292	1.406
1R,3S	1R,3R	1S,3S	1S,3R	0.231	0.202	0.092	19.79	2.200	0.201	1.157
1S,3S	1R,3R	1S,3R	1R,3S	0.062	0.055	-0.138	16.95	2.391	0.054	1.315
1R,3R	1S,3R	1S,3S	1R,3S	0.004	0.046	-0.116	16.28	2.453	0.004	1.352
1R,3R	1R,3S	1S,3S	1S,3R	-0.017	0.037	-0.083	14.77	2.476	-0.015	1.436
1S,3S	1R,3R	1R,3S	1S,3R	-0.068	-0.021	-0.192	13.42	2.529	-0.059	1.511
1R,3S	1S,3R	1S,3S	1R,3R	-0.087	-0.016	-0.172	12.88	2.548	-0.075	1.541
1S,3S	1S,3R	1R,3R	1R,3S	-0.112	-0.012	-0.215	12.01	2.573	-0.097	1.589
1S,3S	1R,3S	1R,3R	1S,3R	-0.133	-0.021	-0.182	10.50	2.595	-0.115	1.673
1R,3S	1R,3R	1S,3R	1S,3S	-0.208	-0.125	-0.336	13.24	2.669	-0.181	1.521
1R,3S	1S,3S	1R,3R	1S,3R	-0.217	-0.024	-0.231	10.21	2.678	-0.189	1.689
1R,3R	1S,3S	1S,3R	1R,3S	-0.270	-0.113	-0.361	11.64	2.729	-0.235	1.610
1S,3S	1R,3S	1S,3R	1R,3R	-0.276	-0.172	-0.369	8.52	2.735	-0.240	1.783
1R,3S	1S,3S	1S,3R	1R,3R	-0.361	-0.175	-0.418	8.23	2.814	-0.314	1.799
1R,3S	1S,3R	1R,3R	1S,3S	-0.382	-0.191	-0.413	8.30	2.833	-0.332	1.795
1S,3S	1S,3R	1R,3S	1R,3R	-0.386	-0.239	-0.456	6.51	2.837	-0.335	1.895
1R,3R	1S,3S	1R,3S	1S,3R	-0.401	-0.189	-0.415	8.11	2.851	-0.348	1.806
1R,3R	1R,3S	1S,3R	1S,3S	-0.456	-0.289	-0.511	8.21	2.900	-0.396	1.800
1R,3R	1S,3R	1R,3S	1S,3S	-0.565	-0.356	-0.598	6.20	2.997	-0.491	1.912

Table S10b. Complete list of permutations with scores for comparison of experimental and calculated ^{1}H NMR data for compound tetrad **5**

Permutation				^{1}H data score						
**5a**	**5b**	**5c**	**5d**	**CP1**	**CP2**	**CP3**	**overlap**	**RMS**	**correl**	**MAE**
1S,3R	1R,3R	1S,3S	1R,3S	1.260	0.657	0.654	1.61	0.116	0.827	0.081
1S,3R	1S,3S	1R,3R	1R,3S	0.927	0.524	0.476	1.24	0.157	0.608	0.118
1R,3S	1R,3R	1S,3S	1S,3R	0.822	0.313	0.303	1.16	0.168	0.539	0.126
1R,3R	1S,3R	1S,3S	1R,3S	0.621	0.187	0.081	0.95	0.187	0.408	0.148
1S,3R	1R,3S	1S,3S	1R,3R	0.530	0.293	0.117	0.96	0.195	0.347	0.146
1R,3S	1S,3S	1R,3R	1S,3R	0.489	0.180	0.125	0.79	0.199	0.321	0.163
1R,3S	1S,3R	1S,3S	1R,3R	0.407	0.027	-0.106	0.73	0.205	0.267	0.170
1R,3R	1R,3S	1S,3S	1S,3R	0.306	0.109	-0.047	0.73	0.214	0.201	0.169
1R,3R	1S,3S	1S,3R	1R,3S	0.196	-0.102	-0.182	0.65	0.222	0.128	0.177
1S,3R	1S,3S	1R,3S	1R,3R	0.108	-0.003	-0.177	0.85	0.229	0.071	0.157
1R,3S	1S,3S	1S,3R	1R,3R	-0.018	-0.262	-0.370	0.43	0.238	-0.012	0.199
1S,3R	1R,3S	1R,3R	1S,3S	-0.068	0.276	-0.176	0.80	0.241	-0.045	0.162

1R,3R	1S,3S	1R,3S	1S,3R	-0.116	-0.187	-0.341	0.62	0.245	-0.076	0.180
1S,3S	1S,3R	1R,3R	1R,3S	-0.130	0.049	-0.334	0.61	0.246	-0.085	0.181
1S,3R	1R,3R	1R,3S	1S,3S	-0.157	0.114	-0.292	1.05	0.247	-0.103	0.137
1R,3S	1S,3R	1R,3R	1S,3S	-0.190	0.011	-0.399	0.56	0.250	-0.125	0.186
1S,3S	1R,3R	1S,3R	1R,3S	-0.223	-0.107	-0.419	0.68	0.252	-0.146	0.174
1R,3S	1R,3R	1S,3R	1S,3S	-0.283	-0.145	-0.484	0.64	0.256	-0.186	0.179
1S,3S	1R,3S	1R,3R	1S,3R	-0.446	-0.029	-0.462	0.39	0.266	-0.292	0.203
1S,3S	1R,3R	1R,3S	1S,3R	-0.535	-0.191	-0.578	0.64	0.272	-0.351	0.178
1R,3R	1S,3R	1R,3S	1S,3S	-0.796	-0.355	-0.865	0.39	0.288	-0.522	0.203
1R,3R	1R,3S	1S,3R	1S,3S	-0.799	-0.349	-0.834	0.21	0.288	-0.524	0.221
1S,3S	1S,3R	1R,3S	1R,3R	-0.950	-0.477	-0.987	0.21	0.296	-0.623	0.221
1S,3S	1R,3S	1S,3R	1R,3R	-0.953	-0.471	-0.956	0.03	0.297	-0.625	0.239

Data is sorted according to Pearson correlation coefficient (correl), permutation corresponding to correct assignment is highlighted in green, scores corresponding to best match of the data (highest CP1, CP2, CP3, aggregate overlap, correlation; and lowest RMS deviation and MAE) are highlighted in blue.

Table S11a. Complete list of permutations with scores for comparison of experimental and calculated ^{13}C NMR data for compound tetrad **6**

**Permutation**				**^{13}C data score**						
**6a**	**6b**	**6c**	**6d**	**CP1**	**CP2**	**CP3**	**overlap**	**RMS**	**correl**	**MAE**
2R,4R	2S,4R	2S,4S	2R,4S	0.858	0.525	0.479	23.29	1.193	0.657	0.995
2R,4R	2S,4S	2S,4R	2R,4S	0.554	0.442	0.297	19.23	1.517	0.424	1.199
2R,4R	2S,4R	2R,4S	2S,4S	0.539	0.428	0.279	21.08	1.531	0.413	1.106
2R,4S	2S,4R	2S,4S	2R,4R	0.490	0.356	0.192	17.82	1.576	0.375	1.269
2R,4R	2S,4S	2R,4S	2S,4R	0.423	0.467	0.243	19.68	1.637	0.324	1.176
2R,4R	2R,4S	2S,4S	2S,4R	0.355	0.230	0.073	18.30	1.696	0.272	1.245
2R,4S	2S,4S	2S,4R	2R,4R	0.186	0.273	0.010	13.76	1.834	0.142	1.472
2R,4S	2R,4R	2S,4S	2S,4R	0.171	0.099	-0.100	15.86	1.845	0.131	1.367
2R,4R	2R,4S	2S,4R	2S,4S	0.166	0.109	-0.072	15.64	1.849	0.127	1.378
2S,4R	2R,4R	2S,4S	2R,4S	0.054	-0.221	-0.307	14.90	1.934	0.042	1.415
2R,4S	2S,4R	2R,4R	2S,4S	0.041	0.185	-0.119	13.83	1.944	0.032	1.468
2R,4S	2R,4R	2S,4R	2S,4S	-0.017	-0.023	-0.245	13.20	1.987	-0.013	1.500
2S,4R	2S,4S	2R,4S	2R,4R	-0.062	-0.021	-0.251	13.24	2.019	-0.047	1.498
2R,4S	2S,4S	2R,4R	2S,4R	-0.075	0.224	-0.155	12.44	2.028	-0.057	1.538
2S,4R	2R,4S	2S,4S	2R,4R	-0.130	-0.258	-0.421	11.86	2.067	-0.100	1.567
2S,4S	2S,4R	2R,4S	2R,4R	-0.145	-0.082	-0.319	12.84	2.077	-0.111	1.518
2S,4R	2S,4S	2R,4R	2R,4S	-0.192	-0.095	-0.363	11.47	2.109	-0.147	1.587
2S,4R	2R,4R	2R,4S	2S,4S	-0.265	-0.317	-0.506	12.68	2.159	-0.203	1.526
2S,4S	2S,4R	2R,4R	2R,4S	-0.275	-0.156	-0.431	11.07	2.166	-0.211	1.607
2S,4S	2R,4R	2S,4R	2R,4S	-0.334	-0.364	-0.556	10.43	2.204	-0.256	1.639
2S,4S	2R,4R	2R,4S	2S,4R	-0.465	-0.339	-0.611	10.88	2.288	-0.356	1.616

2S,4S	2R,4S	2S,4R	2R,4R	-0.519	-0.402	-0.670	7.39	2.322	-0.397	1.790
2S,4R	2R,4S	2R,4R	2S,4S	-0.579	-0.429	-0.732	7.88	2.359	-0.444	1.766
2S,4S	2R,4S	2R,4R	2S,4R	-0.779	-0.451	-0.836	6.07	2.478	-0.597	1.856

Table S11b. Complete list of permutations with scores for comparison of experimental and calculated ^{1}H NMR data for compound tetrad **6**

Permutation				^{1}H data score						
**6a**	**6b**	**6c**	**6d**	**CP1**	**CP2**	**CP3**	**overlap**	**RMS**	**correl**	**MAE**
2R,4R	2S,4R	2S,4S	2R,4S	1.817	0.435	0.303	1.32	0.131	0.713	0.096
2R,4S	2S,4R	2S,4S	2R,4R	1.508	0.417	0.196	1.20	0.141	0.591	0.102
2R,4R	2S,4R	2R,4S	2S,4S	1.432	0.327	0.080	1.22	0.143	0.561	0.101
2R,4S	2R,4R	2S,4S	2S,4R	1.090	0.371	0.048	1.16	0.153	0.428	0.104
2S,4R	2R,4R	2S,4S	2R,4S	1.001	0.353	-0.030	1.11	0.156	0.393	0.107
2S,4S	2S,4R	2R,4S	2R,4R	0.841	0.287	-0.196	0.95	0.160	0.330	0.115
2S,4R	2R,4R	2R,4S	2S,4S	0.615	0.245	-0.252	1.01	0.166	0.241	0.112
2R,4S	2S,4R	2R,4R	2S,4S	0.604	0.178	-0.360	0.91	0.167	0.237	0.117
2S,4S	2R,4R	2R,4S	2S,4R	0.423	0.241	-0.344	0.91	0.171	0.166	0.117
2R,4S	2R,4R	2S,4R	2S,4S	0.390	0.233	-0.381	0.98	0.172	0.153	0.113
2S,4S	2S,4R	2R,4R	2R,4S	0.322	0.157	-0.530	0.76	0.174	0.126	0.124
2S,4S	2R,4R	2S,4R	2R,4S	0.108	0.212	-0.550	0.83	0.179	0.042	0.121
2R,4R	2R,4S	2S,4S	2S,4R	-0.075	-0.210	-0.890	0.86	0.184	-0.029	0.119
2R,4R	2S,4S	2R,4S	2S,4R	-0.238	-0.130	-0.965	0.88	0.188	-0.093	0.118
2S,4R	2R,4S	2S,4S	2R,4R	-0.474	-0.246	-1.074	0.69	0.193	-0.186	0.128
2R,4R	2S,4S	2S,4R	2R,4S	-0.553	-0.159	-1.171	0.80	0.195	-0.217	0.122
2S,4R	2S,4S	2R,4S	2R,4R	-0.637	-0.166	-1.150	0.71	0.197	-0.250	0.127
2R,4R	2R,4S	2S,4R	2S,4S	-0.775	-0.347	-1.318	0.68	0.200	-0.304	0.128
2R,4S	2S,4S	2S,4R	2R,4R	-0.862	-0.178	-1.279	0.68	0.202	-0.338	0.128
2R,4S	2S,4S	2R,4R	2S,4R	-1.066	-0.279	-1.406	0.57	0.206	-0.418	0.134
2S,4R	2S,4S	2R,4R	2R,4S	-1.156	-0.297	-1.483	0.52	0.208	-0.453	0.136
2S,4S	2R,4S	2S,4R	2R,4R	-1.366	-0.387	-1.595	0.41	0.213	-0.536	0.142
2S,4R	2R,4S	2R,4R	2S,4S	-1.378	-0.485	-1.630	0.40	0.213	-0.540	0.142
2S,4S	2R,4S	2R,4R	2S,4R	-1.571	-0.488	-1.722	0.30	0.217	-0.616	0.147

Data is sorted according to Pearson correlation coefficient (correl), permutation corresponding to correct assignment is highlighted in green, scores corresponding to best match of the data (highest CP1, CP2, CP3, aggregate overlap, correlation; and lowest RMS deviation and MAE) are highlighted in blue.

Table S12a. Complete list of permutations with scores for comparison of experimental and calculated ^{13}C NMR data for compound tetrad **7**

Permutation				^{13}C data score						
**7a**	**7b**	**7c**	**7d**	**CP1**	**CP2**	**CP3**	**overlap**	**RMS**	**correl**	**MAE**

3R,5R	3R,5S	3S,5S	3S,5R	0.910	0.634	0.623	29.46	0.599	0.842	0.459
3R,5R	3S,5S	3R,5S	3S,5R	0.836	0.552	0.536	26.90	0.715	0.774	0.576
3S,5R	3R,5S	3S,5S	3R,5R	0.560	0.390	0.320	23.58	1.040	0.518	0.726
3S,5R	3S,5S	3R,5S	3R,5R	0.486	0.308	0.233	21.03	1.111	0.450	0.843
3R,5R	3R,5S	3S,5R	3S,5S	0.178	0.114	-0.003	17.83	1.368	0.165	0.988
3R,5S	3R,5R	3S,5S	3S,5R	0.170	0.136	0.016	17.50	1.374	0.157	1.003
3R,5R	3S,5S	3S,5R	3R,5S	0.110	0.077	-0.062	16.42	1.419	0.102	1.052
3R,5R	3S,5R	3S,5S	3R,5S	0.085	0.130	-0.046	15.73	1.437	0.079	1.083
3R,5R	3S,5R	3R,5S	3S,5S	0.080	0.085	-0.073	14.59	1.441	0.074	1.135
3S,5S	3R,5R	3R,5S	3S,5R	0.045	-0.011	-0.119	14.31	1.465	0.042	1.148
3R,5S	3S,5S	3R,5R	3S,5R	0.042	0.035	-0.124	15.36	1.467	0.039	1.100
3S,5S	3R,5S	3R,5R	3S,5R	-0.009	-0.030	-0.173	14.73	1.503	-0.009	1.129
3S,5R	3R,5R	3S,5S	3R,5S	-0.020	-0.038	-0.165	13.80	1.510	-0.018	1.171
3S,5R	3R,5R	3R,5S	3S,5S	-0.025	-0.083	-0.192	12.66	1.514	-0.023	1.223
3R,5S	3S,5S	3S,5R	3R,5R	-0.050	0.006	-0.184	14.24	1.531	-0.047	1.151
3R,5S	3S,5R	3S,5S	3R,5R	-0.075	0.060	-0.168	13.56	1.548	-0.070	1.182
3S,5R	3R,5S	3R,5R	3S,5S	-0.080	-0.102	-0.246	13.08	1.550	-0.074	1.204
3S,5S	3R,5S	3S,5R	3R,5R	-0.102	-0.058	-0.233	13.61	1.565	-0.094	1.180
3S,5R	3S,5S	3R,5R	3R,5S	-0.148	-0.138	-0.306	11.66	1.595	-0.137	1.268
3S,5S	3S,5R	3R,5S	3R,5R	-0.201	-0.088	-0.303	10.37	1.629	-0.186	1.327
3R,5S	3R,5R	3S,5R	3S,5S	-0.562	-0.385	-0.610	5.88	1.844	-0.520	1.531
3S,5S	3R,5R	3S,5R	3R,5S	-0.682	-0.486	-0.718	3.82	1.910	-0.631	1.625
3R,5S	3S,5R	3R,5R	3S,5S	-0.715	-0.432	-0.734	3.05	1.928	-0.661	1.660
3S,5S	3S,5R	3R,5R	3R,5S	-0.835	-0.534	-0.842	1.00	1.991	-0.772	1.753

Table S12b. Complete list of permutations with scores for comparison of experimental and calculated ^{1}H NMR data for compound tetrad **7**

**Permutation**				**^{1}H data score**						
**7a**	**7b**	**7c**	**7d**	**CP1**	**CP2**	**CP3**	**overlap**	**RMS**	**correl**	**MAE**
3S,5R	3R,5S	3S,5S	3R,5R	0.698	0.435	0.365	0.98	0.058	0.469	0.042
3R,5R	3R,5S	3S,5S	3S,5R	0.608	0.120	0.101	0.99	0.061	0.409	0.041
3S,5S	3R,5S	3S,5R	3R,5R	0.371	0.196	0.125	0.78	0.068	0.249	0.049
3S,5R	3R,5R	3S,5S	3R,5S	0.364	0.395	0.168	0.84	0.068	0.244	0.047
3S,5R	3S,5S	3R,5S	3R,5R	0.268	0.181	-0.003	0.77	0.070	0.180	0.050
3S,5S	3R,5R	3R,5S	3S,5R	0.225	0.157	-0.034	0.76	0.071	0.151	0.050
3R,5R	3R,5S	3S,5R	3S,5S	0.215	-0.047	-0.163	0.79	0.072	0.144	0.049
3R,5R	3S,5S	3R,5S	3S,5R	0.178	-0.134	-0.267	0.78	0.073	0.120	0.050
3S,5S	3R,5S	3R,5R	3S,5R	0.173	0.178	-0.039	0.73	0.073	0.116	0.051
3S,5R	3R,5R	3R,5S	3S,5S	0.158	0.228	-0.058	0.76	0.073	0.106	0.050
3S,5R	3R,5S	3R,5R	3S,5S	0.106	0.250	-0.063	0.73	0.074	0.071	0.051
3R,5R	3S,5R	3S,5S	3R,5S	0.057	-0.109	-0.254	0.62	0.076	0.039	0.056

3S,5S	3R,5R	3S,5R	3R,5S	0.036	0.156	-0.072	0.63	0.076	0.025	0.055
3S,5S	3S,5R	3R,5S	3R,5R	0.008	-0.032	-0.193	0.53	0.077	0.006	0.059
3R,5R	3S,5S	3S,5R	3R,5S	-0.010	-0.135	-0.305	0.65	0.077	-0.007	0.054
3R,5S	3R,5R	3S,5S	3S,5R	-0.104	-0.117	-0.375	0.70	0.079	-0.070	0.052
3S,5R	3S,5S	3R,5R	3R,5S	-0.119	0.163	-0.205	0.59	0.080	-0.080	0.057
3R,5R	3S,5R	3R,5S	3S,5S	-0.148	-0.275	-0.481	0.54	0.080	-0.099	0.058
3R,5S	3S,5R	3S,5S	3R,5R	-0.321	-0.306	-0.534	0.47	0.084	-0.216	0.061
3S,5S	3S,5R	3R,5R	3R,5S	-0.378	-0.050	-0.395	0.35	0.086	-0.254	0.066
3R,5S	3S,5S	3S,5R	3R,5R	-0.389	-0.332	-0.584	0.51	0.086	-0.261	0.060
3R,5S	3R,5R	3S,5R	3S,5S	-0.498	-0.284	-0.639	0.50	0.088	-0.335	0.060
3R,5S	3S,5S	3R,5R	3S,5R	-0.587	-0.349	-0.748	0.46	0.090	-0.394	0.062
3R,5S	3S,5R	3R,5R	3S,5S	-0.913	-0.491	-0.962	0.22	0.096	-0.613	0.071

Data is sorted according to Pearson correlation coefficient (correl), permutation corresponding to correct assignment is highlighted in green, scores corresponding to best match of the data (highest CP1, CP2, CP3, aggregate overlap, correlation; and lowest RMS deviation and MAE) are highlighted in blue.

S5. Plots of ^{1}H and ^{13}C NMR spectra of new compounds

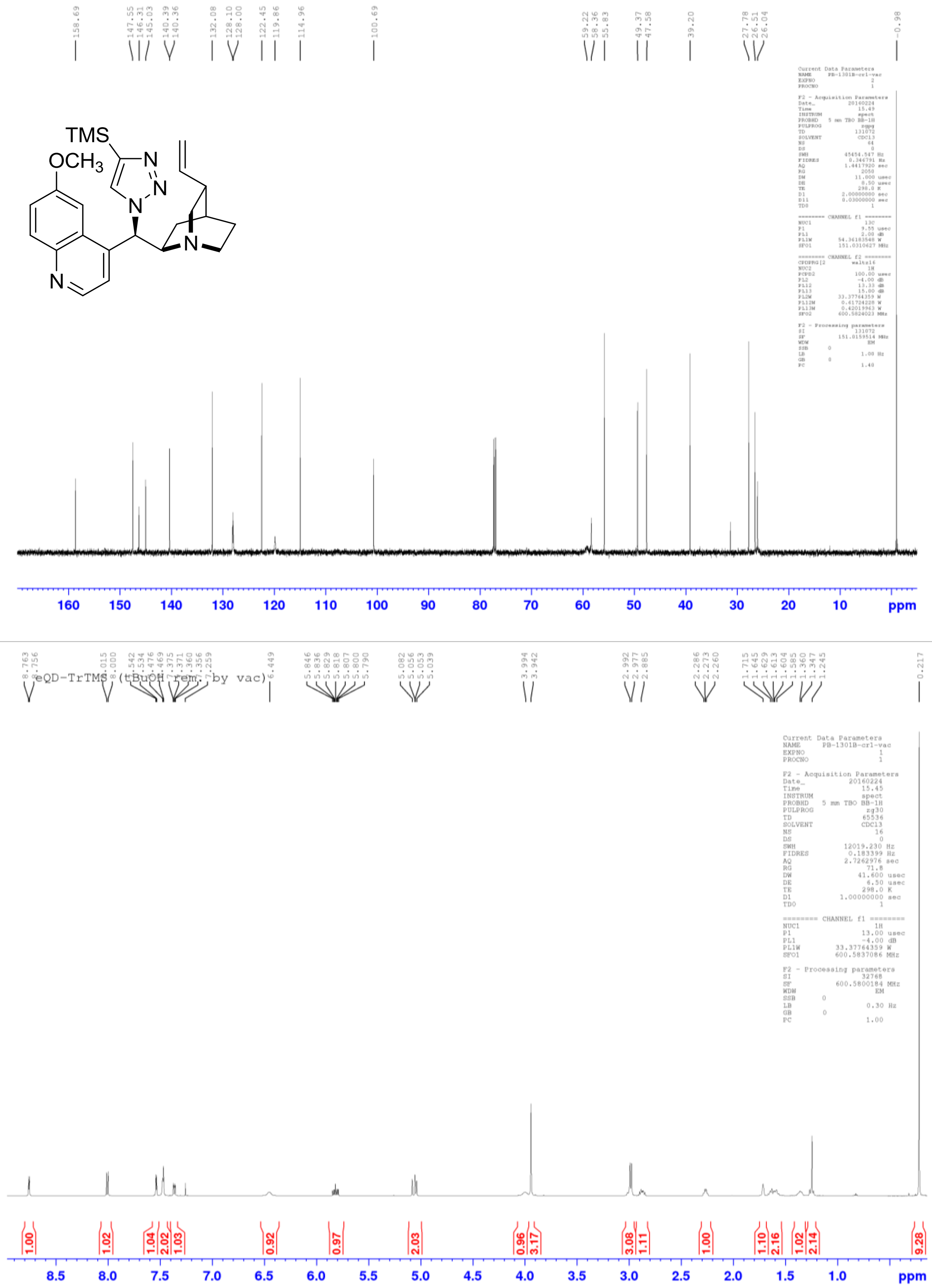

Figure S1. ^{1}H and ^{13}C NMR spectra for 9*R*-(4-trimethylsilyl-1,2,3-triazol-1-yl)-9-deoxyquinidine. Sample contains approx. 10 %mol of tBuOH (^{1}H NMR: 1.24 ppm (s))

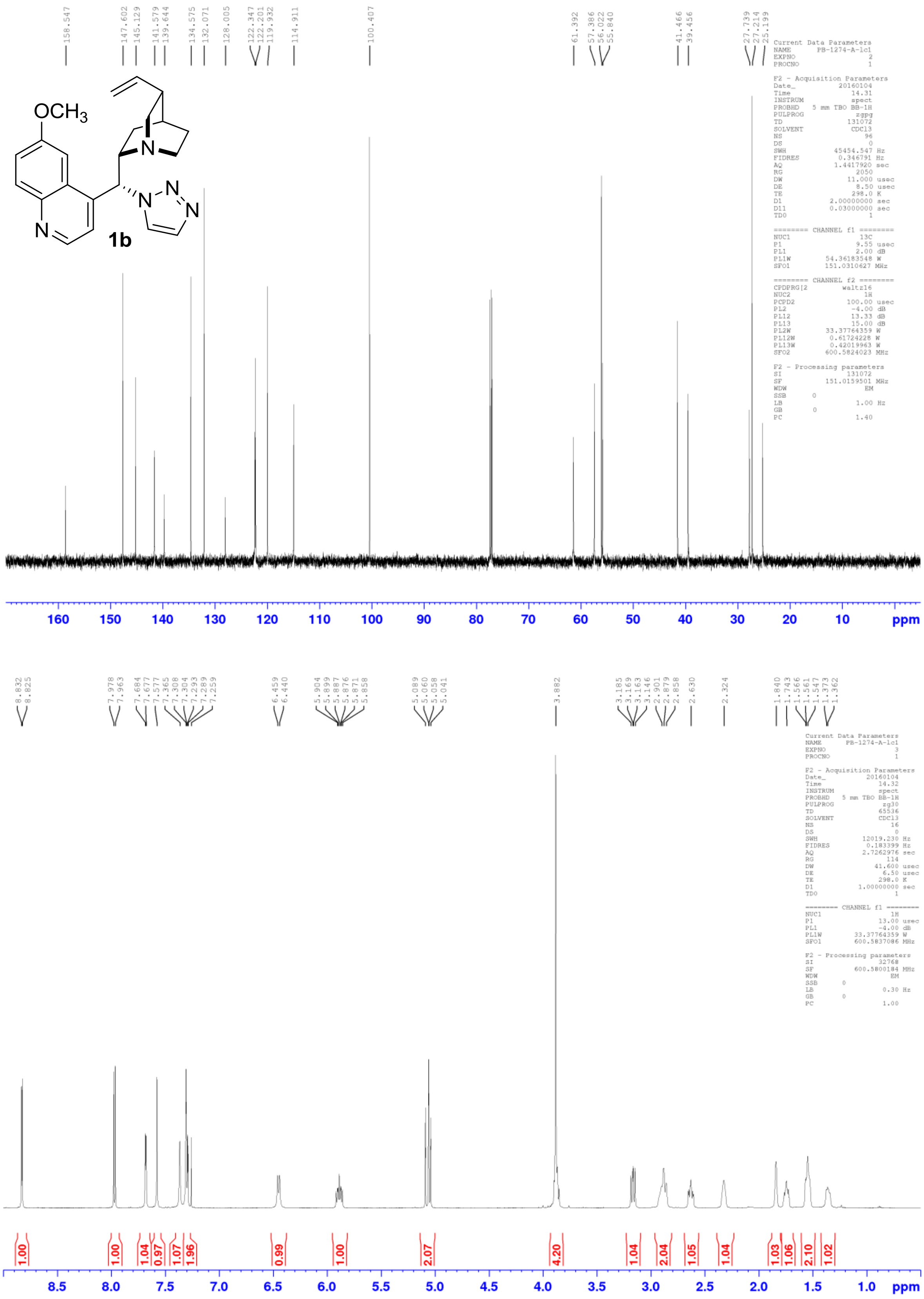

Figure S2. ^{1}H and ^{13}C NMR spectra for **1b**

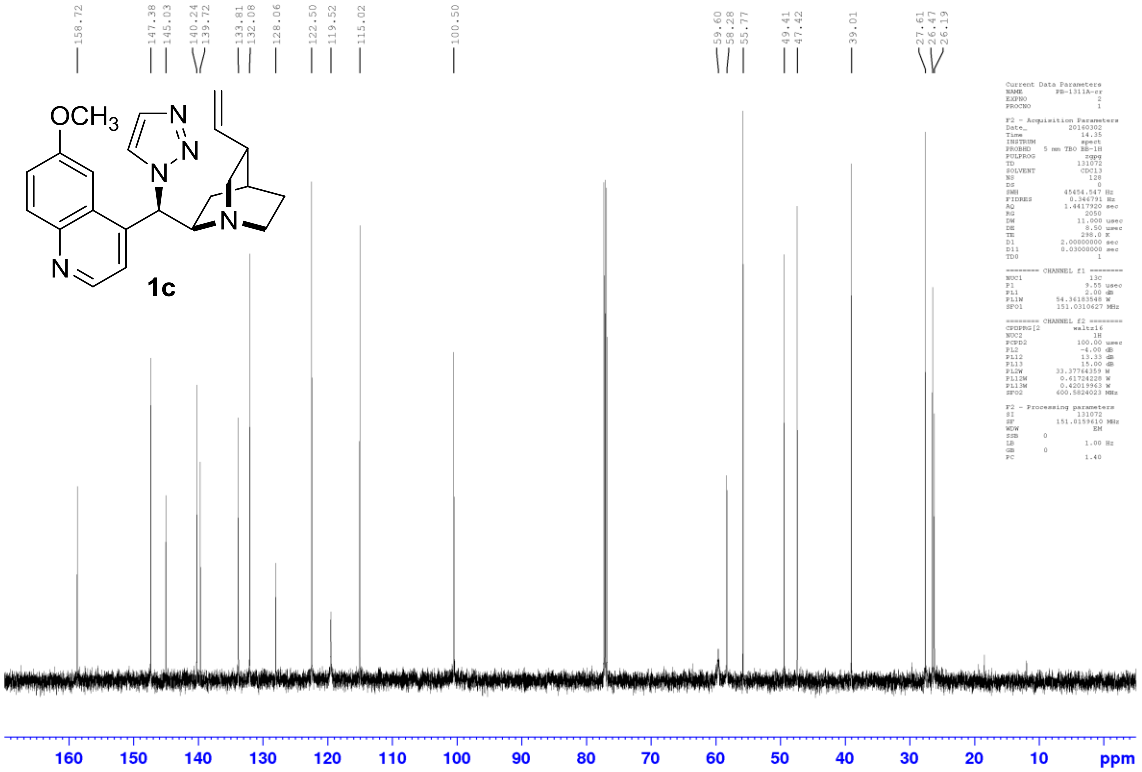

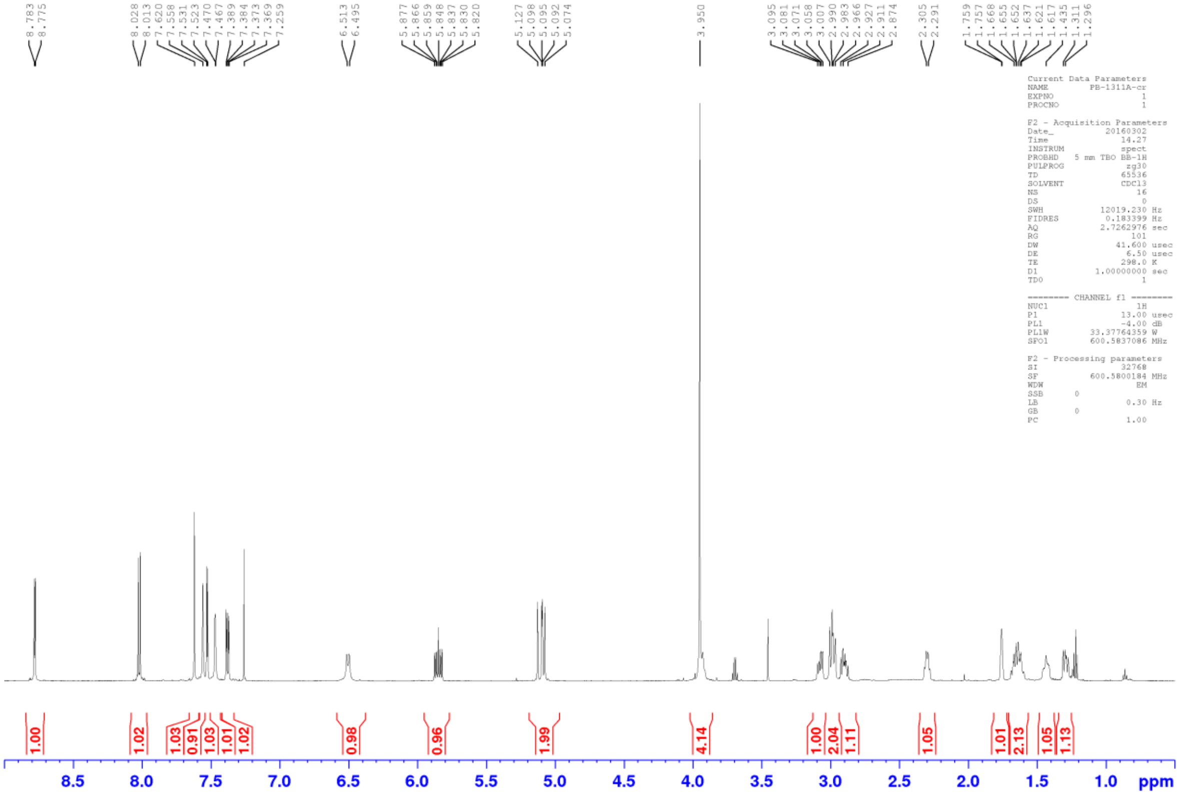

Figure S3. ^{1}H and ^{13}C NMR spectra for **1c**

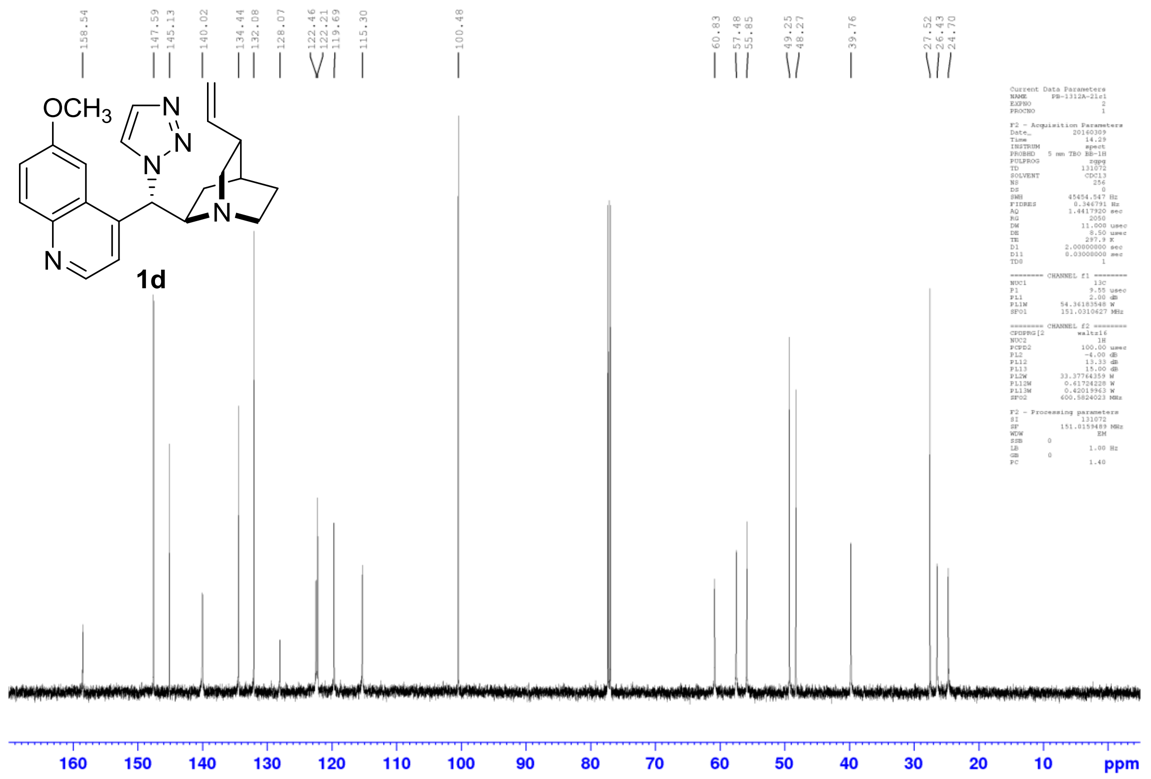

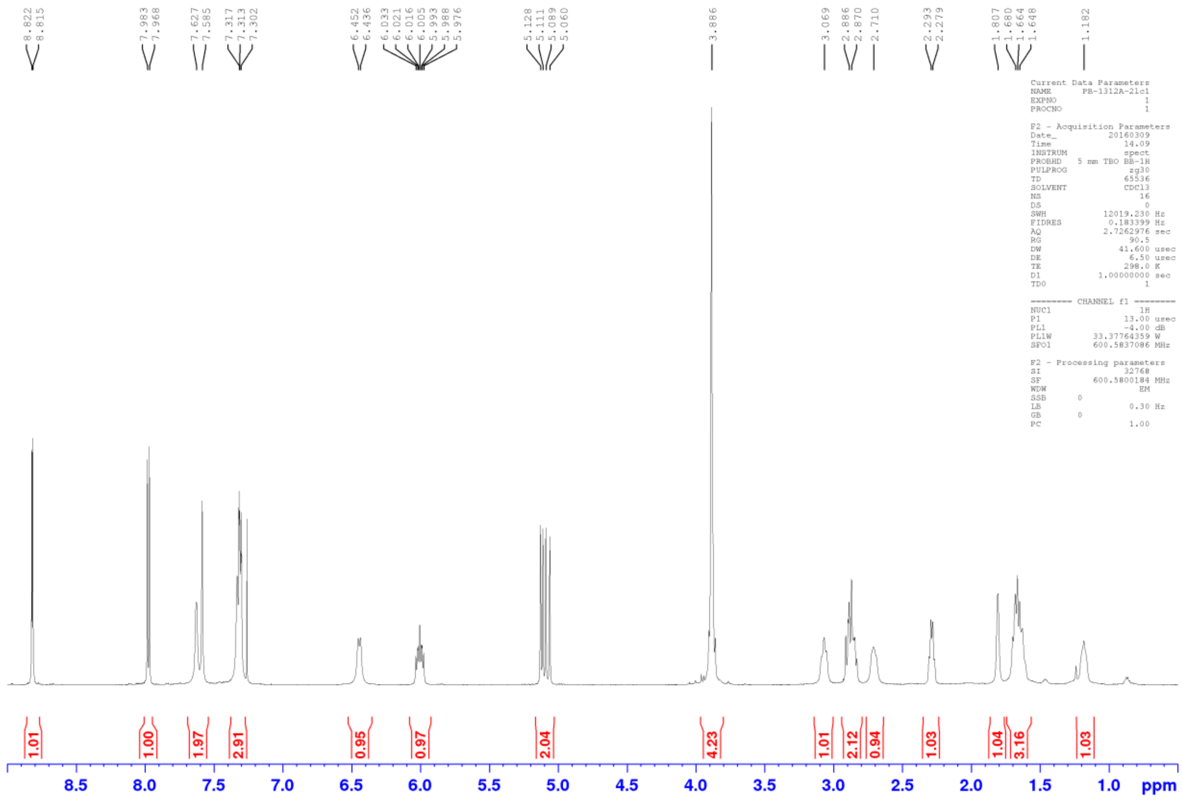

Figure S4. ^{1}H and ^{13}C NMR spectra for **1d**

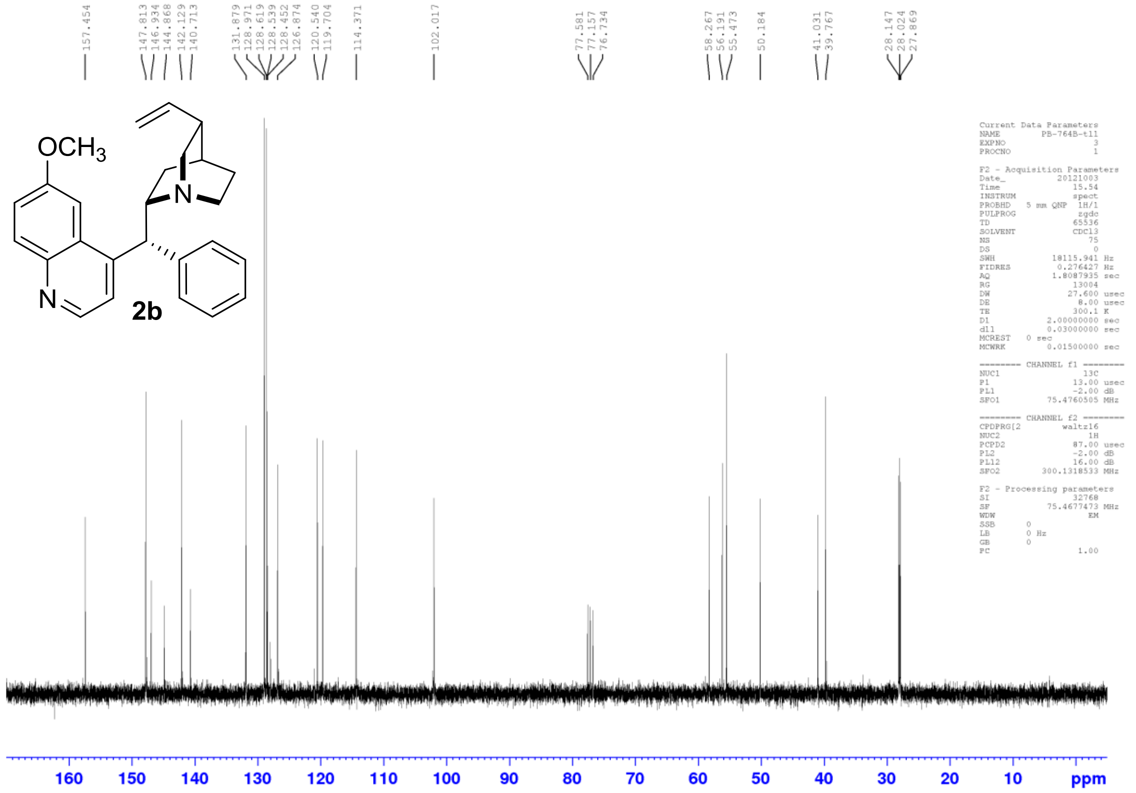

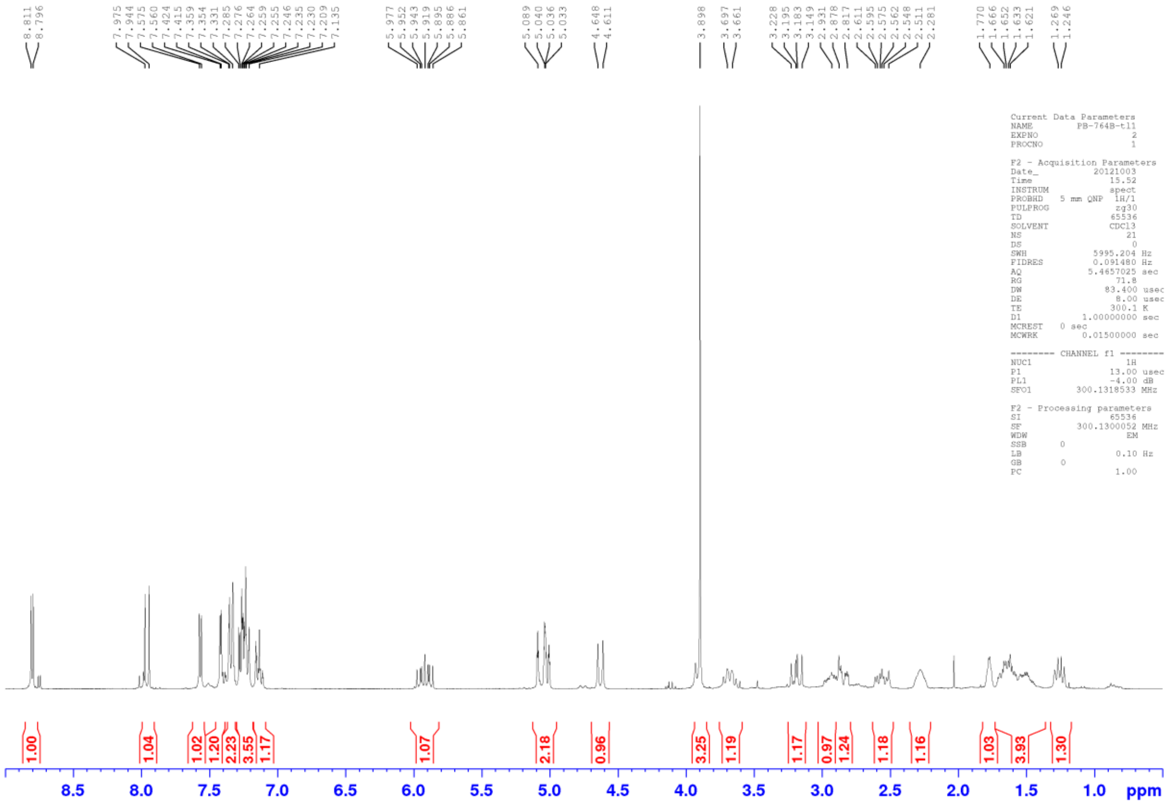

Figure S5. ^{1}H and ^{13}C NMR spectra for **2b**. Trace contamination with **2a** is visible

S5. Plots of $^{1}H,^{13}C$ HSQC experiments for tetrad 1

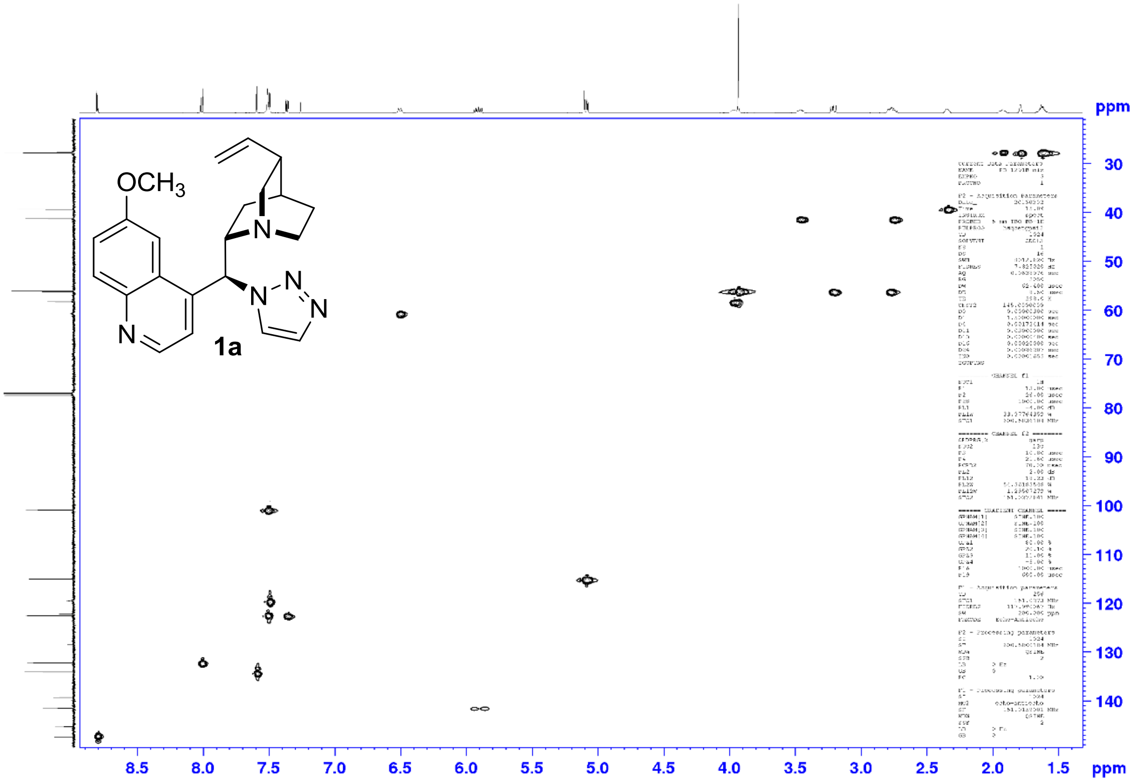

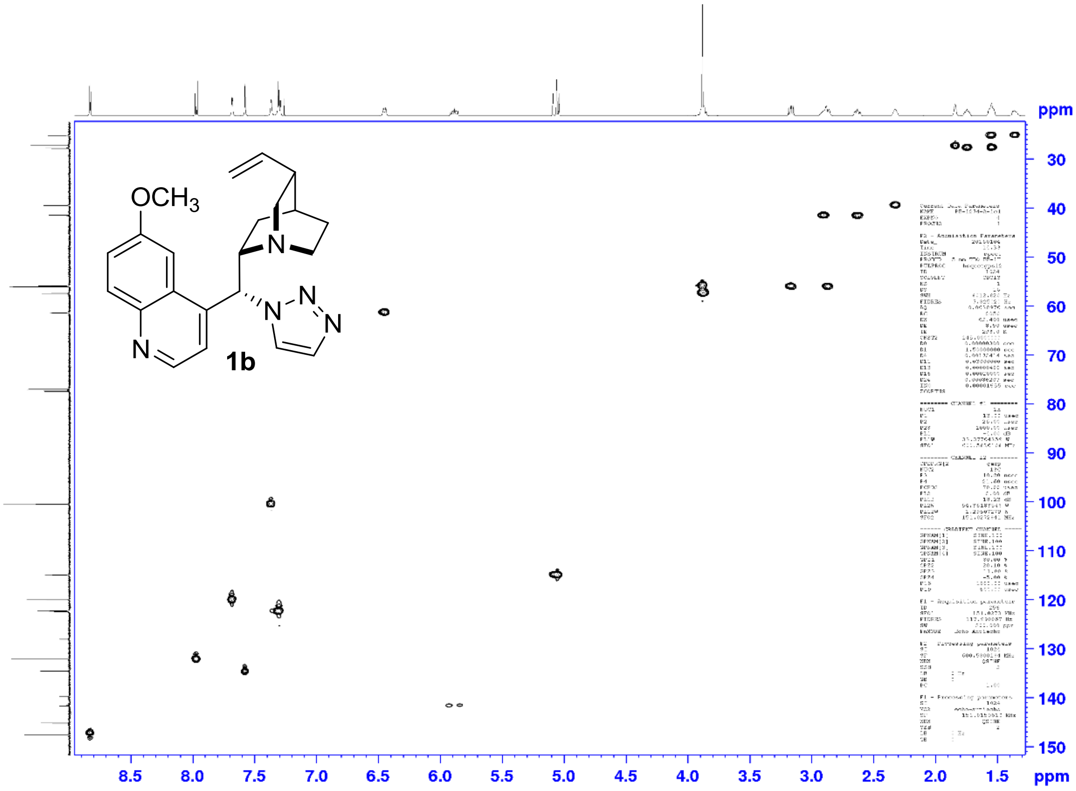

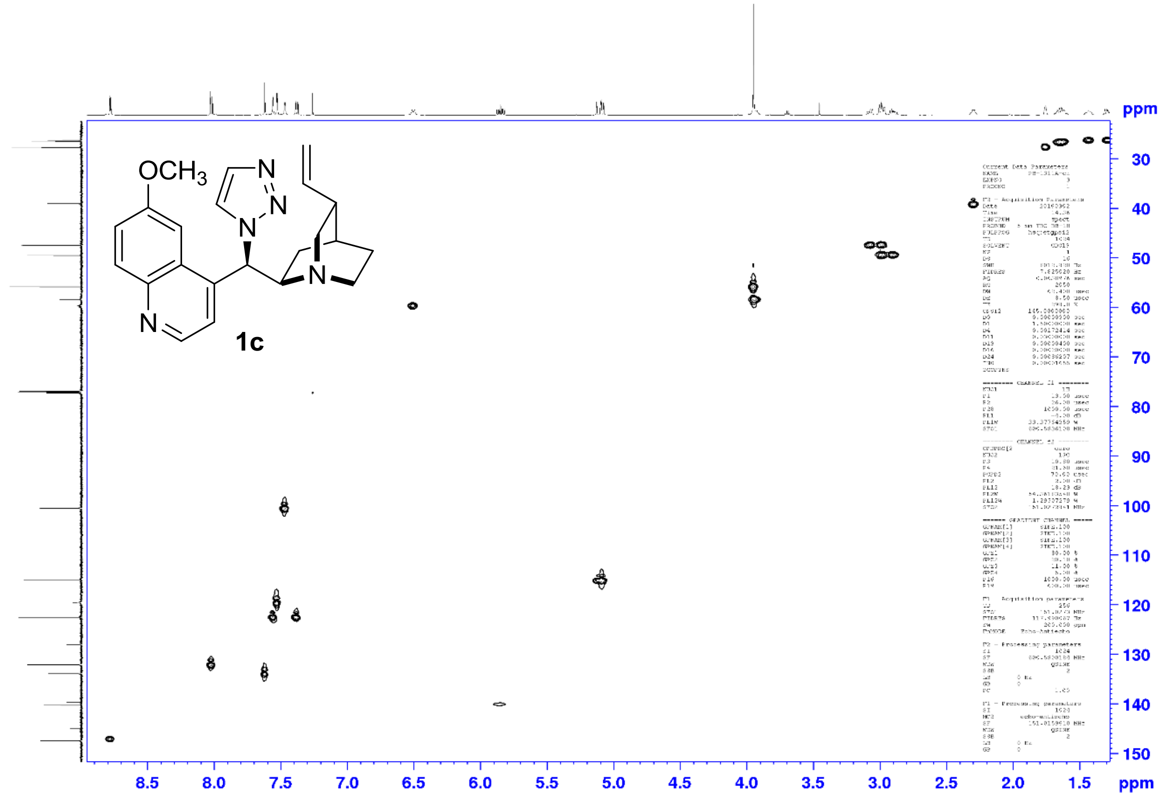
OCH3
N
N
N
N
N
1c
ppm
30
40
50
60
70
80
90
100
110
120
130
140
150
8.5
8.0
7.5
7.0
6.5
6.0
5.5
5.0
4.5
4.0
3.5
3.0
2.5
2.0
1.5
ppm

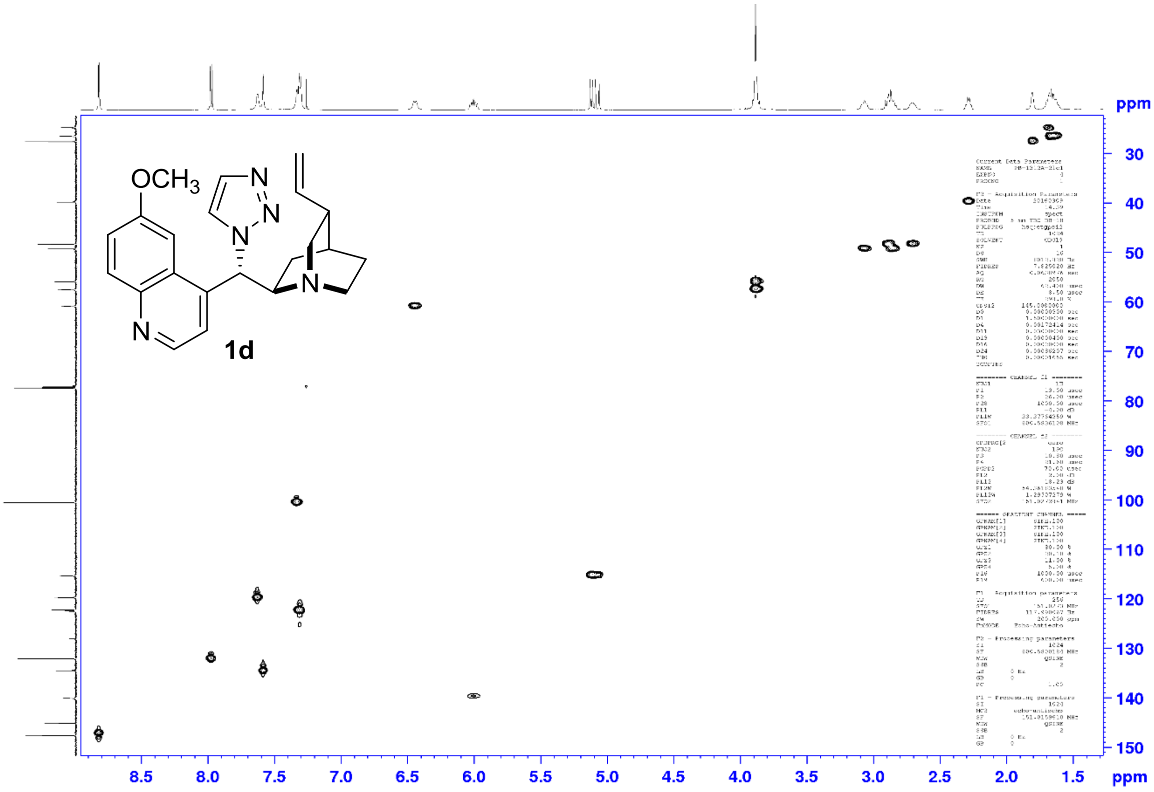
OCH3
N
N
N
N
N
1d
ppm
30
40
50
60
70
80
90
100
110
120
130
140
150
8.5
8.0
7.5
7.0
6.5
6.0
5.5
5.0
4.5
4.0
3.5
3.0
2.5
2.0
1.5
ppm

S7. Computer program (python) for quick calculation of permutations and their scores

Prerequisites: python 2.7, open source libraries: openpyxl, numpy. Excel file (Book1.xlsx) arranged as in the example below.

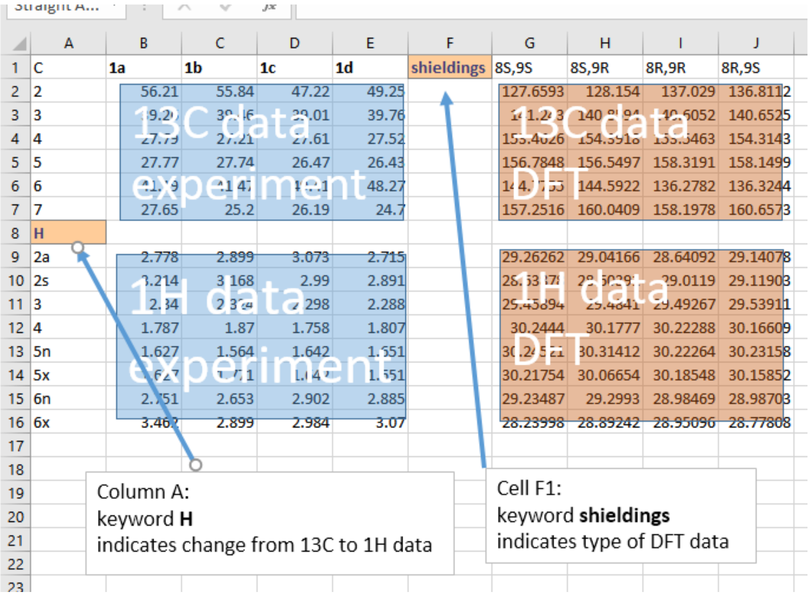

	A	B	C	D	E	F	G	H	I	J
1	C	1a	1b	1c	1d	shieldings	8S,9S	8S,9R	8R,9R	8R,9S
2	2	56.21	55.84	47.22	49.25		127.6593	128.154	137.029	136.8112
3	3	[illegible]	[illegible]	[illegible]	39.76		[illegible]	[illegible]	[illegible]	140.6525
4	4	27.79	27.21	27.61	27.52		[illegible]	[illegible]	[illegible]	154.3143
5	5	27.77	27.74	26.47	26.43		156.7848	156.5497	158.3191	158.1499
6	6	[illegible]	[illegible]	[illegible]	48.27		[illegible]	144.5922	136.2782	136.3244
7	7	27.65	25.2	26.19	24.7		157.2516	160.0409	158.1978	160.6573
8	H									
9	2a	2.778	2.899	3.073	2.715		29.26262	29.04166	28.64092	29.14078
10	2s	[illegible]	3.168	2.99	2.891		[illegible]	[illegible]	29.0119	29.11903
11	3	[illegible]	[illegible]	[illegible]	2.288		[illegible]	[illegible]	[illegible]	29.53911
12	4	1.787	1.87	1.758	1.807		30.2444	30.1777	30.22288	30.16609
13	5n	1.627	1.564	1.642	1.651		[illegible]	30.31412	30.22264	30.23158
14	5x	[illegible]	[illegible]	[illegible]	1.651		30.21754	30.06654	30.18548	30.15852
15	6n	[illegible]	2.653	2.902	2.885		29.23487	29.2993	28.98469	28.98703
16	6x	[illegible]	2.899	2.984	3.07		28.23998	28.89242	28.95096	28.77808

Program code (filename: code.py, intended for Public-domain 🄍):

```
## Required libraries: numpy - for calculations and openpyxl - for handling excel files
## Use python code.py [filename] [-1 (for shieldings)], otherwise program
## will take Book1.xlsx
from openpyxl import Workbook
from openpyxl import load_workbook
from itertools import permutations
from operator import itemgetter
from statistics import mean
import sys
import numpy

def comparison_measure(x, y, z):           #main comparison routine returns a/b from imput lists
# x[] -experimental list, y[] -dft list;
#comparison_measure: z=0 sum product, z = 1 CP1, z= 2 CP2, Z = 3 CP3,
#Z = 4 overlap, Z = 5 RMS deviation, Z = 6 correlation coefficent, Z = 7 MAE
  a=0                                           #variable a result
  b=0                                           #divisor, if necessary
  if (z == 6):                                  #correlation
      a = numpy.corrcoef(x, y)[0, 1]
      b = 1
  elif (z==0):
      for i in range (min(len(x),len(y))):      #sum of products
        a+=x[i]*y[i]
      b=1
  elif (z==1):
      for i in range (min(len(x),len(y))):      #CP1 Sum(exp*dft)/Sum(exp^2)
        b += x[i]*x[i]
        a += x[i]*y[i]
  elif (z==2):                             #CP2 Sum f/Sum(exp^2),
      for i in range (min(len(x),len(y))): # where f=(exp)^3/dft if |dft/exp|>1 otherwise exp*dft
        b += x[i]*x[i]
        if (x[i]<>0 and y[i]<>0):          #when dft=0 or exp=0 avoid division by 0
            if (abs(y[i]/x[i])>1):
              a += x[i]*x[i]*x[i]/y[i]
            else:
```

```
             a += x[i]*y[i]
  elif (z==3):                               #CP3 Sum f/Sum(exp^2),
      for i in range (min(len(x),len(y))):   #where f=(exp)^3/dft if |dft/exp|>1 otherwise exp*dft
        b += x[i]*x[i]
        if (x[i]<>0 and y[i]<>0):            #when dft=0 or exp=0 avoid division by 0
            if ((y[i]/x[i])>1):
              a += x[i]*x[i]*x[i]/y[i]
            else:
             a += x[i]*y[i]
  elif (z == 4):                             # for overlap
      for i in range (min(len(x),len(y))):
        if (x[i]*y[i]>0):                    # if signs are equal
          a += min (abs(x[i]), abs(y[i]))    # count report min absolute value
      b=1
  elif (z==5):
      for i in range (min(len(x),len(y))):                    #RMS deviation
        b += 1
        a += (x[i]-y[i])*(x[i]-y[i])
      a=a**.5
      b=b**.5
  elif (z==7):
      for i in range (min(len(x),len(y))):                    #MAE
        b += 1
        a += abs(x[i]-y[i])
  if (b==0):          #unexpected
   return 0
  else:
   return a/b

shf = 1                       #for dft shifts = 1, for shieldings = -1
if len(sys.argv)<2:
 excelfilename='Book1.xlsx'
else:
 excelfilename=sys.argv[1]
 if len(sys.argv)>2:
  if sys.argv[2]==-1:
    shf=-1
excelfile = load_workbook(excelfilename, data_only=True)
sheet = excelfile.active
split_row=0
if sheet.cell(row=1, column=6).value=='shieldings':         #cell F1 keyword "shieldings"
    shf=-1                                                  #then use DFT data as shieldings
for row in range (2, sheet.max_row+1):                      #prepare input sheet
    xtab,otab,xabs,oabs=[],[],0, 0
    newnuclei=False
    test = sheet.cell(row=row, column=1).value
    if (test == None):
        break
    if (test == "H"):         #keword "H" in column A separates 13C and 1H data
        split_row=row
        continue
    for col in range (1,5):
     if (sheet.cell(row=row, column=col).value == None):
        break
     xtab.append(float(sheet.cell(row=row, column=col+1).value))
     otab.append(float(sheet.cell(row=row, column=col+6).value))
    sheet.cell(row=row, column=12).value=mean(xtab)
    sheet.cell(row=row, column=13).value=mean(otab)
    for col in range (1,5):
     sheet.cell(row=row, column=14+col).value=\
       +sheet.cell(row=row, column=col+1).value-sheet.cell(row=row, column=12).value
     sheet.cell(row=row, column=19+col).value=\
        (+sheet.cell(row=row, column=col+6).value-sheet.cell(row=row, column=13).value)*shf
sheet.cell(row=1, column=12).value="avg exper"
sheet.cell(row=1, column=13).value="avg dft"
sheet.cell(row=1, column=15).value="dev exper"
sheet.cell(row=1, column=20).value="dev dft"
sheet.append(["H","experiment","","","","","dft"])
xc,xh,yc,yh=[],[],[],[]
for i in range (1,5):
    x,y=[],[]
    for k in range (2,split_row):
         if (sheet.cell(row=k, column=14+i).value == None or \
```

```
                        sheet.cell(row=k, column=19+i).value == None):
                 continue
          x.append(sheet.cell(row=k, column=14+i).value)
          y.append(sheet.cell(row=k, column=19+i).value)
     xc.append(x)
     yc.append(y)
     x,y=[],[]
     for k in range (split_row,sheet.max_row):
             if (sheet.cell(row=k, column=14+i).value == None or \
                        sheet.cell(row=k, column=19+i).value == None):
                 continue
             x.append(sheet.cell(row=k, column=14+i).value)
             y.append(sheet.cell(row=k, column=19+i).value)
     xh.append(x)
     yh.append(y)
permu=[]                                #create permutation indexed list permu
permu = list(permutations("1234"))      #create permutation list
for i in range (0, len(permu)):         #create permutation list
     k=[]                                 #create permutation list
     for j in range (0,4):                #create permutation list
        k.append(int(permu[i][j]))        #create permutation list
     permu[i]=k                           #
for i in range (len(permu)):
  x,y=[],[]
  for j in range(4):
    for t in range (len(xc[j])):
      x.append(xc[j][t])
      y.append(yc[permu[i][j]-1][t])
  for met in range(8):
    permu[i].append(comparison_measure(x,y,met))

for i in range (len(permu)):
  x,y=[],[]
  for j in range(4):
    for t in range (len(xc[j])):
      x.append(xh[j][t])
      y.append(yh[permu[i][j]-1][t])
  for met in range(8):
    permu[i].append(comparison_measure(x,y,met))
permu = sorted(permu, key=itemgetter(10), reverse = True)
name_of_operation=["product", "CP1", "CP2", "CP3", "overlap", "RMS", "correl", "MAE","H_product",
"H_CP1", "H_CP2", "H_CP3", "H_overlap", "H_RMS", "H_correl", "H_MAE",]
resultsheet=excelfile.create_sheet("Result")
resultsheet.sheet_properties.tabColor = "00FFFF"
for j in range (4):
 resultsheet.cell(row=1, column=j+1).value=sheet.cell(row=1, column=2+j).value
for j in range (len(name_of_operation)):
  resultsheet.cell(row=1, column=j+5).value=name_of_operation[j]
for i in range (len(permu)):
 for j in range (len(permu[i])):
  if j<4:
   resultsheet.cell(row=i+2, column=j+1).value=sheet.cell(row=1, column=6+permu[i][j]).value
      else:
        resultsheet.cell(row=i+2, column=j+1).value=permu[i][j]
excelfile.save(excelfilename.replace(".xlsx","-result.xlsx"))
```

S8. Cartesian coordinates for gas phase optimized geometries of tetrads 1–3

1a-conformer 1 [mPW1PW91/6-311+G(2d,p)]

atom	X	Y	Z
C	-0.136453	0.853889	-0.411364
C	0.925017	0.886628	0.684104
C	0.725448	1.657693	1.806586
C	1.691079	1.681519	2.829064
N	2.811200	0.996361	2.789955
C	3.042206	0.230229	1.694486
C	4.257371	-0.503390	1.651683
C	4.568814	-1.293910	0.586546
C	3.672450	-1.395171	-0.506665
C	2.483438	-0.703089	-0.503063
C	2.131658	0.130757	0.595652
O	4.097198	-2.211504	-1.509950
C	3.271322	-2.369005	-2.654078
C	-1.425654	0.122413	0.025513
C	-2.012964	-1.079446	-2.018652
C	-2.038749	-2.469298	-1.316643
C	-2.276241	-2.227701	0.182367
C	-1.180027	-1.266723	0.680998
C	-3.690218	-1.605975	0.343222
C	-3.703283	-0.290297	-0.520164
N	-2.381852	0.005190	-1.093178
C	-4.083762	-1.362909	1.770158
C	-5.088174	-1.965912	2.397211
H	0.267854	0.353916	-1.287627
H	-0.164086	2.267224	1.900433
H	1.524426	2.294344	3.710312
H	4.925873	-0.407953	2.497785
H	5.493682	-1.855366	0.545364
H	1.814615	-0.798125	-1.343888
H	2.303403	-2.805194	-2.388543
H	3.803775	-3.048927	-3.315210
H	3.115374	-1.413790	-3.164912
H	-1.904741	0.779238	0.752437
H	-1.026037	-0.867804	-2.435437
H	-2.711004	-1.045903	-2.857461
H	-1.095154	-3.002021	-1.465105
H	-2.830000	-3.101765	-1.728153
H	-2.227793	-3.168501	0.733980
H	-0.204087	-1.676881	0.407272
H	-1.182806	-1.183204	1.768667
H	-4.406730	-2.317076	-0.079457
H	-4.417907	-0.381841	-1.340702
H	-4.012202	0.566425	0.081421
H	-3.502618	-0.622036	2.316234
H	-5.706781	-2.705606	1.898635
H	-5.330820	-1.741497	3.428980
N	-0.462521	2.207574	-0.883198
C	-0.020774	2.826642	-2.001113
C	-0.594598	4.072213	-1.954817
H	0.636301	2.351952	-2.708819
H	-0.516400	4.891227	-2.649655
N	-1.266629	3.027822	-0.180541
N	-1.347506	4.156804	-0.827772

Energies in solvent (SMD)

SCF = -1202.37577335

Sum of electronic and zero-point Energies= -1201.930975

Sum of electronic and thermal Energies= -1201.907832

Sum of electronic and thermal Enthalpies= -1201.906887

Sum of electronic and thermal Free Energies= -1201.983488

Number of imaginary frequencies:

0

1a - conformer 2 [mPW1PW91/6-311+G(2d,p)]

C	0.127840	-0.212099	0.209910
C	-0.755780	1.010111	0.441003
C	-0.291963	2.146307	1.061591
C	-1.142117	3.255253	1.246539
N	-2.392442	3.286969	0.851093
C	-2.889886	2.178843	0.243412
C	-4.244710	2.207786	-0.178857
C	-4.817547	1.125237	-0.777223
C	-4.061792	-0.056697	-0.985934
C	-2.740256	-0.119288	-0.604185
C	-2.119992	0.996971	0.015550
O	-4.748560	-1.072737	-1.566364
C	-4.073130	-2.309718	-1.781333
C	1.646316	0.064721	0.248036
C	2.351993	-1.821764	-1.138553
C	2.864102	-0.870648	-2.260739
C	3.309174	0.445184	-1.601708
C	2.093672	1.050006	-0.876453
C	4.448148	0.116943	-0.598756
C	3.831471	-0.853499	0.473815
N	2.429662	-1.181557	0.187613
C	5.080678	1.327086	0.019655
C	6.352514	1.684338	-0.125090
H	-0.112217	-0.618429	-0.773034
H	0.728791	2.221761	1.410793
H	-0.760595	4.147225	1.735845
H	-4.804609	3.117334	-0.002391
H	-5.852011	1.134535	-1.097135
H	-2.184232	-1.033353	-0.742101
H	-3.234982	-2.183814	-2.472521
H	-4.811028	-2.975945	-2.221848
H	-3.708689	-2.731332	-0.841488
H	1.881860	0.495972	1.224541
H	1.327118	-2.145916	-1.315360
H	2.953716	-2.730916	-1.087583
H	2.077517	-0.669148	-2.993589
H	3.698225	-1.320573	-2.805589
H	3.676453	1.144655	-2.355343
H	1.290169	1.210585	-1.601727
H	2.329813	2.032734	-0.464885
H	5.227072	-0.414095	-1.154636
H	4.399117	-1.785183	0.514002
H	3.881399	-0.405387	1.469374
H	4.438760	1.944739	0.646086
H	7.040118	1.102851	-0.731027
H	6.753431	2.568239	0.356418
N	-0.244351	-1.293699	1.130511
C	-0.257439	-1.317991	2.480811
C	-0.653196	-2.593874	2.796841
H	-0.006952	-0.463986	3.084949
H	-0.799999	-3.046549	3.762933
N	-0.613550	-2.493689	0.635674
N	-0.863369	-3.279933	1.643141

Energies in solvent (SMD)

SCF = -1202.37770714

Sum of electronic and zero-point Energies= -1201.932427

Sum of electronic and thermal Energies= -1201.909437

Sum of electronic and thermal Enthalpies= -1201.908493

Sum of electronic and thermal Free Energies= -1201.984540

Number of imaginary frequencies:

0

1a - conformer 3 [mPW1PW91/6-311+G(2d,p)]

C	0.173471	-1.414260	-0.202798
C	-1.049502	-1.039368	-1.038323
C	-1.254124	-1.750682	-2.199938
C	-2.321359	-1.436195	-3.061680
N	-3.173235	-0.466846	-2.827271
C	-3.014834	0.250430	-1.684878
C	-3.944033	1.294253	-1.436891
C	-3.867207	2.052847	-0.307338
C	-2.850027	1.802066	0.647651
C	-1.919425	0.809075	0.438331
C	-1.971726	0.008846	-0.733153
O	-2.894225	2.600274	1.744844
C	-1.936719	2.381454	2.778466
C	1.259391	-0.319705	-0.097012
C	3.285846	-1.641362	-0.464000
C	3.767944	-0.764623	-1.656321
C	3.097196	0.611632	-1.519755
C	1.574677	0.384554	-1.445887
C	3.636509	1.271899	-0.221091
C	3.301140	0.283065	0.956723
N	2.496436	-0.860000	0.503691
C	3.109720	2.656173	0.016117
C	3.851325	3.758394	0.049717
H	0.625234	-2.266599	-0.711124
H	-0.585323	-2.560158	-2.471180
H	-2.464439	-2.006729	-3.975237
H	-4.717588	1.458796	-2.176048
H	-4.576131	2.846330	-0.106268
H	-1.175993	0.599519	1.189879
H	-1.997979	1.362936	3.168341
H	-2.182707	3.092774	3.563403
H	-0.921252	2.570923	2.419387
H	0.896180	0.422418	0.611178
H	2.685969	-2.483331	-0.814350
H	4.129561	-2.073443	0.077487
H	3.497292	-1.220962	-2.612474
H	4.855996	-0.657029	-1.650860
H	3.339514	1.242606	-2.377042
H	1.269035	-0.226753	-2.299746
H	1.020697	1.321054	-1.527345
H	4.723471	1.346969	-0.323171
H	4.220919	-0.102264	1.401707
H	2.751234	0.794437	1.749000
H	2.038095	2.749512	0.183830
H	4.925313	3.725198	-0.104346
H	3.414098	4.732547	0.233333
N	-0.175016	-1.935627	1.132424
C	-0.487568	-3.212382	1.452394
C	-0.728394	-3.182760	2.802344
H	-0.513291	-4.004428	0.724402
H	-1.005654	-3.981512	3.469221
N	-0.217903	-1.158115	2.230653
N	-0.554889	-1.908713	3.240960

Energies in solvent (SMD)

SCF = -1202.37403101

Sum of electronic and zero-point Energies= -1201.928856

Sum of electronic and thermal Energies= -1201.905837

Sum of electronic and thermal Enthalpies= -1201.904893

Sum of electronic and thermal Free Energies= -1201.980948

Number of imaginary frequencies:

0

1b - conformer 1 [mPW1PW91/6-311+G(2d,p)]

C	-0.136787	-0.898596	1.063386
C	1.072882	-1.391897	0.276761
C	1.290871	-2.749988	0.242832
C	2.361507	-3.289775	-0.493575
N	3.208150	-2.556240	-1.175802
C	3.037973	-1.208658	-1.151228
C	3.965248	-0.420787	-1.881772
C	3.878723	0.939289	-1.894747
C	2.851859	1.591319	-1.167390
C	1.922946	0.863378	-0.458768
C	1.986679	-0.554992	-0.432617
O	2.884508	2.947955	-1.235035
C	1.912398	3.685183	-0.498928
C	-1.256864	-0.244716	0.217812
C	-2.370097	-2.276249	-0.565471
C	-3.726474	-1.888519	0.093664
C	-3.764515	-0.357335	0.222063
C	-2.537514	0.081072	1.041932
C	-3.722975	0.241123	-1.210152
C	-2.391559	-0.273901	-1.871735
N	-1.588675	-1.086218	-0.946760
C	-3.839625	1.735826	-1.242502
C	-4.833598	2.411524	-1.809362
H	-0.566965	-1.779164	1.544557
H	0.629659	-3.425318	0.774371
H	2.513864	-4.365443	-0.516059
H	4.745361	-0.942117	-2.421691
H	4.585704	1.548623	-2.443828
H	1.162088	1.373731	0.108024
H	0.901634	3.472376	-0.858460
H	2.145061	4.733607	-0.670640
H	1.970247	3.464041	0.569477
H	-0.857946	0.680622	-0.193607
H	-1.769923	-2.896036	0.101865
H	-2.524051	-2.864602	-1.472055
H	-3.827653	-2.347807	1.081094
H	-4.568819	-2.239718	-0.508388
H	-4.681089	-0.038680	0.722506
H	-2.540310	-0.456471	1.996099
H	-2.570565	1.141416	1.289891
H	-4.575972	-0.169147	-1.759466
H	-2.615402	-0.881515	-2.750929
H	-1.778769	0.564753	-2.208935
H	-3.034021	2.295999	-0.771275
H	-5.659147	1.903853	-2.298269
H	-4.859105	3.494673	-1.806092
N	0.241158	-0.018427	2.187614
C	0.815592	-0.387880	3.355916
C	0.990444	0.786091	4.043226
H	1.050016	-1.411840	3.589545
H	1.411229	0.954408	5.020066
N	0.072388	1.319720	2.155870
N	0.523568	1.806457	3.277136

Energies in solvent (SMD)

SCF = -1202.37463188

Sum of electronic and zero-point Energies= -1201.929464

Sum of electronic and thermal Energies= -1201.906468

Sum of electronic and thermal Enthalpies= -1201.905523

Sum of electronic and thermal Free Energies= -1201.981352

Number of imaginary frequencies:

0

1b - conformer 2 [mPW1PW91/6-311+G(2d,p)]

C	-0.139418	0.216002	-0.135754
C	0.787779	-0.946680	-0.467126
C	0.345745	-2.074182	-1.116507
C	1.241655	-3.121269	-1.408857
N	2.513781	-3.104563	-1.085886
C	2.985398	-2.006227	-0.441736
C	4.360102	-1.985267	-0.088300
C	4.906631	-0.910978	0.548415
C	4.102368	0.213071	0.866265
C	2.762882	0.228159	0.548428
C	2.168939	-0.881931	-0.109088
O	4.764193	1.226919	1.481456
C	4.037475	2.407181	1.810741
C	-1.659155	-0.071965	-0.182638
C	-1.950714	-0.823441	2.120583
C	-2.915735	0.351511	2.457800
C	-3.610395	0.781082	1.154928
C	-2.519608	1.187625	0.147325
C	-4.435701	-0.425054	0.633207
C	-3.409181	-1.589035	0.383356
N	-2.028952	-1.196327	0.696690
C	-5.259959	-0.117913	-0.580796
C	-6.586481	-0.171818	-0.638063
H	0.104685	0.582528	0.861784
H	-0.693826	-2.193334	-1.384420
H	0.879688	-4.007797	-1.922296
H	4.955821	-2.851728	-0.346015
H	5.954732	-0.883775	0.819134
H	2.168683	1.100005	0.773035
H	3.607749	2.870695	0.919243
H	4.759849	3.082318	2.263499
H	3.240403	2.190759	2.527841
H	-1.895437	-0.399795	-1.199060
H	-0.917751	-0.572648	2.364668
H	-2.194388	-1.715528	2.700486
H	-2.368575	1.197264	2.882441
H	-3.658691	0.046612	3.199608
H	-4.276393	1.625997	1.340322
H	-1.907417	1.978447	0.586581
H	-2.954854	1.607729	-0.761095
H	-5.121810	-0.721332	1.432494
H	-3.662986	-2.457341	0.994919
H	-3.444192	-1.912183	-0.660315
H	-4.713756	0.155687	-1.482055
H	-7.181852	-0.444850	0.227486
H	-7.125816	0.054578	-1.550045
N	0.163639	1.365593	-1.004859
C	0.195075	1.440446	-2.354578
C	0.504739	2.751716	-2.618505
H	0.023587	0.590234	-2.991100
H	0.636197	3.247899	-3.565285
N	0.444064	2.570928	-0.462035
N	0.650065	3.408371	-1.437924

Energies in solvent (SMD)

SCF = -1202.37742888

Sum of electronic and zero-point Energies= -1201.932469

Sum of electronic and thermal Energies= -1201.909423

Sum of electronic and thermal Enthalpies= -1201.908479

Sum of electronic and thermal Free Energies= -1201.984658

Number of imaginary frequencies:

0

1b - conformer 3 [mPW1PW91/6-311+G(2d,p)]

C	0.151152	0.718440	-0.441897
C	-0.899844	0.843554	0.655181
C	-0.649021	1.652025	1.739375
C	-1.601924	1.776624	2.765415
N	-2.760435	1.157377	2.761913
C	-3.046269	0.364365	1.699027
C	-4.306372	-0.290135	1.690127
C	-4.678271	-1.093232	0.654195
C	-3.801061	-1.287351	-0.441689
C	-2.569610	-0.675468	-0.469428
C	-2.152392	0.166022	0.599800
O	-4.289574	-2.105785	-1.415496
C	-3.484109	-2.359791	-2.555840
C	1.475812	0.086042	0.046719
C	1.016626	-2.278710	-0.360066
C	2.258018	-2.461826	-1.282457
C	3.274615	-1.367986	-0.918731
C	2.570228	-0.004876	-1.054090
C	3.735459	-1.617561	0.543712
C	2.438261	-1.588113	1.433315
N	1.241054	-1.235678	0.656422
C	4.781684	-0.653461	1.019052
C	6.018480	-0.986604	1.372814
H	-0.235730	0.115310	-1.260067
H	0.276133	2.210512	1.806464
H	-1.392099	2.414843	3.619080
H	-4.959784	-0.122526	2.536793
H	-5.638023	-1.594195	0.638126
H	-1.916776	-0.838891	-1.312546
H	-3.266325	-1.436383	-3.101800
H	-4.065505	-3.024773	-3.190574
H	-2.546454	-2.851256	-2.278496
H	1.839732	0.725961	0.850828
H	0.130423	-2.019466	-0.940140
H	0.775431	-3.202594	0.169028
H	1.978263	-2.377137	-2.336438
H	2.702921	-3.451797	-1.149546
H	4.138822	-1.411432	-1.584479
H	2.132051	0.067689	-2.055356
H	3.270840	0.825336	-0.965050
H	4.169829	-2.621344	0.581007
H	2.270074	-2.564707	1.891903
H	2.544356	-0.865865	2.245269
H	4.486153	0.391964	1.086332
H	6.363325	-2.015115	1.331716
H	6.730286	-0.245733	1.716685
N	0.428595	2.030561	-1.050196
C	-0.172567	2.597458	-2.121965
C	0.396317	3.842367	-2.217644
H	-0.928340	2.093980	-2.699377
H	0.215841	4.627224	-2.932587
N	1.318351	2.885843	-0.506468
N	1.302420	3.979894	-1.214864

Energies in solvent (SMD)

SCF = -1202.37561629

Sum of electronic and zero-point Energies= -1201.930731

Sum of electronic and thermal Energies= -1201.907665

Sum of electronic and thermal Enthalpies= -1201.906721

Sum of electronic and thermal Free Energies= -1201.982971

Number of imaginary frequencies:

0

1c - conformer 1 [mPW1PW91/6-311+G(2d,p)]

C	0.382153	-0.973079	0.311492
C	-0.883030	-1.198127	-0.509510
C	-0.926871	-2.218462	-1.432118
C	-2.082197	-2.420264	-2.208435
N	-3.162684	-1.679074	-2.113584
C	-3.156260	-0.669754	-1.207006
C	-4.329867	0.122375	-1.098549
C	-4.409315	1.149661	-0.206934
C	-3.307568	1.444825	0.634626
C	-2.149664	0.705699	0.556328
C	-2.038320	-0.373964	-0.364856
O	-3.512209	2.486649	1.486185
C	-2.474926	2.840596	2.389620
C	1.576794	-0.480539	-0.536763
C	3.931121	-0.140336	-0.511544
C	3.740214	0.733682	-1.788466
C	2.480277	1.597969	-1.588407
C	1.244191	0.677638	-1.516242
C	2.645326	2.348806	-0.241041
C	2.554769	1.247320	0.869357
N	2.717464	-0.109131	0.325400
C	1.665261	3.460976	-0.028249
C	1.986908	4.727608	0.215325
H	0.183560	-0.237635	1.086381
H	-0.081617	-2.883972	-1.551718
H	-2.107025	-3.228325	-2.933949
H	-5.158680	-0.124660	-1.749654
H	-5.301331	1.756472	-0.115363
H	-1.322558	0.948919	1.204697
H	-2.234973	2.010632	3.061242
H	-2.858042	3.676100	2.970975
H	-1.571807	3.153822	1.857283
H	1.908211	-1.345091	-1.112403
H	4.146895	-1.178934	-0.767097
H	4.763154	0.222859	0.095618
H	3.623752	0.106456	-2.676836
H	4.612672	1.369081	-1.960667
H	2.370742	2.313801	-2.405100
H	1.003194	0.281580	-2.504620
H	0.368521	1.245697	-1.192910
H	3.648456	2.786763	-0.224255
H	3.320108	1.409542	1.631159
H	1.592531	1.309980	1.383064
H	0.610554	3.194269	-0.073000
H	3.021667	5.050627	0.269615
H	1.229881	5.488129	0.365492
N	0.763778	-2.185842	1.050681
C	0.536975	-2.472216	2.352199
C	1.056342	-3.730752	2.523841
H	0.047740	-1.787248	3.022499
H	1.096532	-4.342740	3.409023
N	1.389585	-3.214159	0.447267
N	1.567809	-4.149978	1.337814

Energies in solvent (SMD)

SCF = -1202.37640527

Sum of electronic and zero-point Energies= -1201.931598

Sum of electronic and thermal Energies= -1201.908547

Sum of electronic and thermal Enthalpies= -1201.907603

Sum of electronic and thermal Free Energies= -1201.983615

Number of imaginary frequencies:

0

1c - conformer 2 [mPW1PW91/6-311+G(2d,p)]

C	-0.261584	0.263418	0.472218
C	0.843228	1.227389	0.049488
C	0.683109	2.592763	0.090018
C	1.730538	3.445792	-0.311967
N	2.892315	3.016818	-0.744563
C	3.091889	1.674193	-0.789052
C	4.350711	1.207519	-1.249831
C	4.630258	-0.125626	-1.300303
C	3.660230	-1.073648	-0.885338
C	2.421462	-0.661771	-0.447001
C	2.104096	0.720710	-0.392892
O	4.066213	-2.366371	-0.954099
C	3.165281	-3.382375	-0.519473
C	-1.688938	0.851184	0.432550
C	-3.920443	0.686024	1.254493
C	-4.429302	1.351893	-0.060340
C	-3.577276	0.810637	-1.222690
C	-2.118380	1.256586	-1.010357
C	-3.675178	-0.737723	-1.196063
C	-2.968851	-1.198268	0.125974
N	-2.677711	-0.068642	1.022210
C	-3.112172	-1.413189	-2.409530
C	-3.802627	-2.182907	-3.244376
H	-0.236370	-0.593473	-0.200830
H	-0.243745	3.042835	0.419193
H	1.588603	4.522462	-0.276215
H	5.079155	1.948977	-1.552352
H	5.589673	-0.491801	-1.643829
H	1.703008	-1.390312	-0.105107
H	2.880665	-3.239167	0.525642
H	3.704017	-4.320557	-0.628935
H	2.266187	-3.402227	-1.141834
H	-1.705690	1.733066	1.078315
H	-3.732792	1.432518	2.029702
H	-4.658478	-0.012050	1.654564
H	-4.338926	2.440912	-0.008913
H	-5.485863	1.128319	-0.228483
H	-3.947485	1.188323	-2.177781
H	-2.034526	2.335993	-1.152754
H	-1.464796	0.800164	-1.758752
H	-4.737732	-0.995609	-1.141523
H	-3.597924	-1.906058	0.668543
H	-2.038671	-1.724667	-0.090034
H	-2.050416	-1.266942	-2.600436
H	-4.861698	-2.371184	-3.099237
H	-3.335557	-2.654562	-4.100645
N	0.045044	-0.322998	1.782201
C	0.227058	0.273270	2.980281
C	0.443424	-0.765922	3.850741
H	0.201749	1.340357	3.113785
H	0.637032	-0.744106	4.909936
N	0.141777	-1.663330	1.904921
N	0.385957	-1.931838	3.155440

Energies in solvent (SMD)

SCF = -1202.37785436

Sum of electronic and zero-point Energies= -1201.932744

Sum of electronic and thermal Energies= -1201.909740

Sum of electronic and thermal Enthalpies= -1201.908795

Sum of electronic and thermal Free Energies= -1201.984868

Number of imaginary frequencies:

0

1c - conformer 3 [mPW1PW91/6-311+G(2d,p)]

C	0.343296	-0.183485	1.038269
C	-0.655671	0.971426	0.996087
C	-0.367139	2.075675	1.767127
C	-1.202161	3.208373	1.747627
N	-2.283771	3.291740	1.010187
C	-2.609870	2.219187	0.243227
C	-3.782504	2.316754	-0.550264
C	-4.188166	1.281331	-1.338155
C	-3.439117	0.078243	-1.370349
C	-2.287626	-0.052471	-0.627094
C	-1.838309	1.013235	0.196058
O	-3.958379	-0.890575	-2.167393
C	-3.292695	-2.150315	-2.220462
C	1.085879	-0.472209	-0.285719
C	2.595305	-1.977598	-1.342155
C	2.805746	-0.881102	-2.429647
C	2.897157	0.481846	-1.716281
C	1.540994	0.797975	-1.053828
C	3.990658	0.359708	-0.623264
C	3.405148	-0.618093	0.451625
N	2.240664	-1.365351	-0.048352
C	4.427236	1.664341	-0.030798
C	5.673367	2.126770	-0.036252
H	1.096779	0.108158	1.770325
H	0.516645	2.088765	2.395696
H	-0.957581	4.070944	2.361436
H	-4.342705	3.241657	-0.500052
H	-5.084971	1.344327	-1.941835
H	-1.747112	-0.984595	-0.630551
H	-2.287653	-2.045798	-2.639052
H	-3.895711	-2.774860	-2.875433
H	-3.226303	-2.606909	-1.230370
H	0.405437	-1.039172	-0.917398
H	1.802945	-2.673579	-1.622843
H	3.503578	-2.565361	-1.193116
H	1.976242	-0.872631	-3.142629
H	3.716058	-1.070556	-3.004257
H	3.163057	1.269069	-2.424016
H	0.797860	1.073573	-1.805061
H	1.637733	1.659808	-0.389515
H	4.869800	-0.102336	-1.083727
H	4.164236	-1.335851	0.768968
H	3.114564	-0.063743	1.346856
H	3.655371	2.263576	0.449331
H	6.483695	1.570716	-0.497018
H	5.929298	3.076567	0.417905
N	-0.233203	-1.424851	1.590212
C	-0.358267	-1.754238	2.896036
C	-0.951434	-2.991003	2.886917
H	-0.028845	-1.109508	3.692123
H	-1.230433	-3.625984	3.710679
N	-0.723202	-2.409947	0.813973
N	-1.159905	-3.356987	1.595475

Energies in solvent (SMD)

SCF = -1202.37483053

Sum of electronic and zero-point Energies= -1201.929744

Sum of electronic and thermal Energies= -1201.906718

Sum of electronic and thermal Enthalpies= -1201.905774

Sum of electronic and thermal Free Energies= -1201.981822

Number of imaginary frequencies:

0

1d - conformer 1 [mPW1PW91/6-311+G(2d,p)]

C	0.230097	0.918341	0.344773
C	-1.104043	1.061087	-0.379872
C	-1.332275	2.172087	-1.158210
C	-2.551176	2.318507	-1.843653
N	-3.526570	1.440536	-1.789924
C	-3.338880	0.338890	-1.020560
C	-4.399982	-0.602218	-0.954345
C	-4.297749	-1.730431	-0.197334
C	-3.115624	-1.982278	0.542653
C	-2.063178	-1.097955	0.501899
C	-2.140462	0.087777	-0.280656
O	-3.137317	-3.139104	1.261814
C	-1.997704	-3.477996	2.037444
C	1.423792	0.726661	-0.621036
C	2.116707	-0.326564	-2.639737
C	3.587284	-0.081659	-2.183774
C	3.669850	-0.374149	-0.673498
C	2.808738	0.658449	0.079937
C	3.101157	-1.798741	-0.447780
C	1.569524	-1.698354	-0.759665
N	1.221956	-0.460535	-1.474474
C	3.366963	-2.365705	0.913045
C	4.003932	-3.506718	1.156560
H	0.194265	0.068732	1.022841
H	-0.582834	2.949378	-1.238780
H	-2.719510	3.198455	-2.458076
H	-5.293004	-0.386125	-1.526847
H	-5.102710	-2.452260	-0.139519
H	-1.169407	-1.312818	1.065685
H	-1.111184	-3.603102	1.408571
H	-2.234675	-4.424186	2.518754
H	-1.800068	-2.721564	2.803544
H	1.413174	1.586863	-1.290919
H	1.752476	0.491122	-3.264299
H	2.032379	-1.243139	-3.227466
H	3.889526	0.951006	-2.378290
H	4.278057	-0.725306	-2.734440
H	4.704740	-0.325738	-0.329652
H	3.284035	1.640016	0.069408
H	2.709365	0.376452	1.132487
H	3.570632	-2.464563	-1.178994
H	1.244763	-2.547986	-1.363691
H	0.995419	-1.743399	0.167301
H	2.992348	-1.792642	1.759867
H	4.397930	-4.120711	0.352921
H	4.157698	-3.867381	2.166645
N	0.479843	2.075685	1.219790
C	0.163073	2.216038	2.528082
C	0.544279	3.496703	2.839528
H	-0.294117	1.430005	3.103642
H	0.480245	4.024131	3.776223
N	1.022571	3.215758	0.745441
N	1.065494	4.073834	1.725709

Energies in solvent (SMD)

SCF = -1202.37642441

Sum of electronic and zero-point Energies= -1201.931236

Sum of electronic and thermal Energies= -1201.908229

Sum of electronic and thermal Enthalpies= -1201.907285

Sum of electronic and thermal Free Energies= -1201.983282

Number of imaginary frequencies:

0

1d - conformer 2 [mPW1PW91/6-311+G(2d,p)]

C	0.256544	0.153640	0.492778
C	-0.823486	1.180425	0.177998
C	-0.633468	2.528043	0.367224
C	-1.668272	3.437980	0.074044
N	-2.842224	3.082168	-0.393003
C	-3.064211	1.758031	-0.595952
C	-4.331266	1.369153	-1.104515
C	-4.628762	0.057048	-1.323895
C	-3.668566	-0.947662	-1.041246
C	-2.426078	-0.611205	-0.553125
C	-2.090405	0.749463	-0.321657
O	-4.087581	-2.215932	-1.286108
C	-3.192084	-3.289351	-1.010728
C	1.700477	0.686211	0.650944
C	3.358770	2.179342	-0.198395
C	4.460351	1.250996	0.394662
C	4.003948	-0.205309	0.196868
C	2.719604	-0.437605	1.013444
C	3.718219	-0.408436	-1.314808
C	2.477082	0.490256	-1.646424
N	2.148791	1.415661	-0.550383
C	3.512902	-1.837306	-1.717835
C	4.247561	-2.488184	-2.613892
H	0.258447	-0.598423	-0.296294
H	0.313228	2.915989	0.714055
H	-1.505918	4.501260	0.228021
H	-5.050070	2.152486	-1.308768
H	-5.593151	-0.250080	-1.708839
H	-1.714754	-1.387901	-0.319774
H	-2.293065	-3.219773	-1.629866
H	-3.734988	-4.198141	-1.259709
H	-2.906511	-3.308127	0.043993
H	1.689950	1.420483	1.461185
H	3.080485	2.964171	0.509402
H	3.707810	2.676712	-1.105665
H	4.609989	1.451277	1.459478
H	5.420298	1.421651	-0.099233
H	4.782543	-0.897967	0.521722
H	2.945025	-0.419349	2.082187
H	2.307643	-1.426660	0.806559
H	4.586031	-0.032738	-1.866354
H	2.663592	1.081627	-2.544877
H	1.603857	-0.127021	-1.863247
H	2.688189	-2.364604	-1.241817
H	5.078801	-2.010553	-3.123032
H	4.045297	-3.520874	-2.871545
N	-0.113935	-0.612925	1.694406
C	-0.438585	-0.173218	2.931204
C	-0.657292	-1.318542	3.656071
H	-0.507131	0.871075	3.180550
H	-0.944654	-1.432391	4.687724
N	-0.134008	-1.963552	1.656914
N	-0.462653	-2.389538	2.842983

Energies in solvent (SMD)

SCF = -1202.37786413

Sum of electronic and zero-point Energies= -1201.932886

Sum of electronic and thermal Energies= -1201.909877

Sum of electronic and thermal Enthalpies= -1201.908933

Sum of electronic and thermal Free Energies= -1201.984886

Number of imaginary frequencies:

0

1d - conformer 3 [mPW1PW91/6-311+G(2d,p)]

C	0.402395	0.108225	0.947429
C	-0.682892	-0.963137	0.975245
C	-0.440616	-2.085203	1.733559
C	-1.373466	-3.137498	1.781341
N	-2.510657	-3.122159	1.127738
C	-2.793681	-2.023945	0.379866
C	-4.029755	-2.010344	-0.317573
C	-4.399313	-0.940994	-1.077631
C	-3.545802	0.185892	-1.177981
C	-2.332516	0.207325	-0.528053
C	-1.919794	-0.896124	0.265006
O	-4.032048	1.198953	-1.942267
C	-3.253045	2.385769	-2.065770
C	1.101559	0.300735	-0.419869
C	1.808890	-0.817169	-2.400028
C	2.810671	0.352867	-2.635330
C	3.416513	0.737329	-1.272075
C	2.295210	1.298076	-0.376088
C	4.004433	-0.552776	-0.642807
C	2.775732	-1.465667	-0.311311
N	1.541128	-0.999815	-0.961953
C	4.877942	-0.317523	0.551316
C	6.149955	-0.689083	0.653410
H	1.164538	-0.214316	1.659242
H	0.482473	-2.179662	2.294548
H	-1.162897	-4.019312	2.380335
H	-4.667596	-2.879254	-0.217508
H	-5.342801	-0.920119	-1.608704
H	-1.706163	1.081201	-0.593781
H	-3.067110	2.843165	-1.091175
H	-3.840470	3.061373	-2.683277
H	-2.297638	2.180146	-2.556931
H	0.356160	0.676345	-1.118551
H	0.859638	-0.637725	-2.908389
H	2.205379	-1.759832	-2.783079
H	2.304562	1.218513	-3.071791
H	3.597973	0.058243	-3.333874
H	4.204552	1.482075	-1.398935
H	1.976681	2.280718	-0.724458
H	2.658232	1.438893	0.646541
H	4.617144	-1.044153	-1.405483
H	2.964920	-2.492035	-0.632083
H	2.614859	-1.507024	0.767644
H	4.416976	0.186321	1.399220
H	6.659919	-1.198507	-0.158069
H	6.727933	-0.497827	1.549663
N	-0.068357	1.403084	1.479810
C	-0.303830	1.715217	2.775495
C	-0.751255	3.011356	2.744464
H	-0.148146	1.016189	3.578716
H	-1.051059	3.654525	3.554439
N	-0.359499	2.455987	0.688242
N	-0.770569	3.428682	1.451618

Energies in solvent (SMD)

SCF = -1202.37512148

Sum of electronic and zero-point Energies= -1201.930141

Sum of electronic and thermal Energies= -1201.907122

Sum of electronic and thermal Enthalpies= -1201.906178

Sum of electronic and thermal Free Energies= -1201.982132

Number of imaginary frequencies:

0

2a - conformer 1 [B3LYP/6-31G(d,p)]

C	-0.266105	2.278815	-0.331469
C	0.682419	2.930532	-1.132513
H	1.466980	2.346963	-1.608768
C	0.644345	4.311577	-1.319764
H	1.391155	4.792055	-1.945518
C	-0.351225	5.071860	-0.705004
H	-0.387197	6.147749	-0.848998
C	-1.303904	4.435990	0.091178
H	-2.089501	5.016256	0.567039
C	-1.263659	3.052781	0.274325
H	-2.031289	2.576856	0.873779
C	-0.131146	0.768382	-0.128523
H	0.217992	0.364852	-1.081811
C	0.936561	0.463444	0.929330
C	0.779735	0.946803	2.215308
H	-0.076276	1.564958	2.467256
C	1.737376	0.666382	3.211188
H	1.593319	1.053477	4.219133
N	2.825579	-0.048843	3.003872
C	3.021385	-0.530656	1.746212
C	4.201008	-1.294620	1.517400
H	4.865519	-1.443701	2.361719
C	4.482480	-1.815249	0.283771
H	5.378739	-2.397565	0.097707
C	3.588684	-1.594878	-0.798973
C	2.432793	-0.860653	-0.618423
H	1.765632	-0.698861	-1.453212
C	2.112368	-0.308750	0.657091
O	3.980275	-2.159574	-1.977082
C	3.151083	-1.986232	-3.114796
H	2.154808	-2.419805	-2.957917
H	3.646694	-2.510769	-3.932670
H	3.042840	-0.926020	-3.377399
C	-1.448362	0.025444	0.220038
H	-1.776125	0.383796	1.202727
C	-2.335583	-0.207144	-2.045461
H	-1.441217	0.244169	-2.484300
H	-3.176646	0.107961	-2.671880
C	-2.228015	-1.763315	-1.990693
H	-1.259162	-2.106171	-2.373696
H	-2.999209	-2.234315	-2.611382
C	-2.387074	-2.197981	-0.519745
H	-2.312664	-3.287086	-0.434858
C	-1.264602	-1.527415	0.298363
H	-0.296789	-1.832949	-0.114538
H	-1.273551	-1.868941	1.337867
C	-3.788297	-1.734746	-0.029637
H	-4.532192	-2.160041	-0.715594
C	-3.804490	-0.165583	-0.157850
H	-4.626400	0.155392	-0.806625
H	-3.972963	0.296532	0.821632
N	-2.550061	0.367361	-0.706479
C	-4.130049	-2.201362	1.358138
H	-3.504232	-1.817407	2.165465
C	-5.135414	-3.022232	1.665412
H	-5.794127	-3.427527	0.900626
H	-5.338852	-3.320057	2.689680

Energies in solvent (SMD)

SCF = -1192.38180481

Sum of electronic and zero-point Energies= -1191.895611

Sum of electronic and thermal Energies= -1191.871940

Sum of electronic and thermal Enthalpies= -1191.870996

Sum of electronic and thermal Free Energies= -1191.950792

Number of imaginary frequencies:

1 (-7 cm^{-1})

Frequencies in vacuum

Number of imaginary frequencies:

0

2a - conformer 2 [B3LYP/6-31G(d,p)]

C	0.620422	2.032271	-0.410046
C	1.706631	2.587630	-1.105224
H	2.003856	2.158418	-2.058149
C	2.420382	3.668211	-0.590988
H	3.257144	4.075470	-1.151454
C	2.064822	4.220557	0.640711
H	2.619747	5.061454	1.046456
C	0.984345	3.684419	1.339337
H	0.686684	4.112711	2.292722
C	0.264916	2.605335	0.818280
H	-0.601972	2.234196	1.349089
C	-0.112111	0.839093	-1.047960
H	-0.546002	1.216688	-1.983059
C	0.865636	-0.257051	-1.485739
C	0.931999	-0.603790	-2.822765
H	0.320917	-0.079399	-3.552497
C	1.777139	-1.647484	-3.260697
H	1.809732	-1.904806	-4.318812
N	2.540826	-2.356283	-2.455572
C	2.517928	-2.032968	-1.131610
C	3.351013	-2.790914	-0.261452
H	3.942102	-3.584090	-0.706637
C	3.407968	-2.521111	1.079408
H	4.044462	-3.087445	1.751200
C	2.632317	-1.461722	1.622827
C	1.800627	-0.711535	0.813731
H	1.242786	0.115421	1.226457
C	1.712374	-0.980740	-0.581590
O	2.795781	-1.269391	2.961996
C	2.077596	-0.210166	3.578216
H	2.331282	0.760866	3.135888
H	2.370104	-0.219697	4.628999
H	0.993231	-0.363379	3.502665
C	-1.296517	0.233206	-0.237639
H	-0.902747	-0.082745	0.734004
C	-3.154383	1.577587	-1.105935
H	-2.500510	2.024111	-1.860146
H	-3.856211	2.362182	-0.804448
C	-3.907070	0.332674	-1.669342
H	-3.661638	0.169652	-2.725150
H	-4.993362	0.468600	-1.612329
C	-3.477911	-0.892138	-0.837924
H	-3.946971	-1.801157	-1.228249
C	-1.943058	-1.009530	-0.931920
H	-1.663145	-1.058949	-1.989761
H	-1.581980	-1.938088	-0.479399
C	-3.935523	-0.662049	0.629856
H	-5.021991	-0.507745	0.616271
C	-3.232113	0.663855	1.105358
H	-3.980531	1.419662	1.366568
H	-2.636754	0.479112	2.007066
N	-2.347161	1.232743	0.077770
C	-3.651036	-1.824659	1.540035
H	-2.599945	-2.077380	1.688485
C	-4.576626	-2.544103	2.176094
H	-5.637828	-2.331322	2.067709
H	-4.310972	-3.371692	2.827026

Energies in solvent (SMD)

SCF = -1192.37973204

Sum of electronic and zero-point Energies= -1191.893368

Sum of electronic and thermal Energies= -1191.868916

Sum of electronic and thermal Enthalpies= -1191.867972

Sum of electronic and thermal Free Energies= -1191.949958

Number of imaginary frequencies:

0

2a - conformer 3 [B3LYP/6-31G(d,p)]

C	-0.217333	1.557627	-0.770570
C	-0.494626	2.711507	-0.027468
H	-0.467336	2.667719	1.058574
C	-0.794114	3.919917	-0.656808
H	-1.004766	4.802119	-0.058704
C	-0.819271	3.995202	-2.049539
H	-1.053048	4.933759	-2.543449
C	-0.545354	2.852639	-2.802481
H	-0.567381	2.897683	-3.887669
C	-0.251680	1.645623	-2.167909
H	-0.061244	0.759980	-2.766924
C	0.129088	0.242687	-0.066274
H	-0.146602	0.358163	0.988167
C	-0.706406	-0.915924	-0.611512
C	-0.216601	-1.809057	-1.546035
H	0.797764	-1.727342	-1.919852
C	-1.023934	-2.864119	-2.028457
H	-0.613646	-3.557654	-2.761732
N	-2.262913	-3.081083	-1.640078
C	-2.787760	-2.212586	-0.730333
C	-4.129930	-2.434083	-0.312232
H	-4.647981	-3.287953	-0.735618
C	-4.740480	-1.596638	0.582101
H	-5.764658	-1.751365	0.904893
C	-4.033612	-0.480619	1.105688
C	-2.724933	-0.239598	0.733921
H	-2.204582	0.626440	1.116483
C	-2.065022	-1.101030	-0.187019
O	-4.754579	0.290398	1.968290
C	-4.132872	1.442507	2.516586
H	-3.256419	1.177022	3.121940
H	-4.881214	1.914165	3.154793
H	-3.826401	2.146293	1.732591
C	1.657991	-0.038307	-0.091141
H	1.952638	-0.116559	-1.144380
C	2.349949	1.218953	1.894550
H	1.306115	1.377902	2.178472
H	2.890278	2.125740	2.186007
C	2.937036	-0.040451	2.602698
H	2.201804	-0.490558	3.279951
H	3.812350	0.218851	3.209823
C	3.328362	-1.050414	1.508005
H	3.702444	-1.976039	1.957513
C	2.072972	-1.344390	0.660475
H	1.266624	-1.685201	1.321615
H	2.256428	-2.163526	-0.040741
C	4.447847	-0.406692	0.638932
H	5.280274	-0.157359	1.309245
C	3.841479	0.923746	0.050475
H	4.408884	1.790151	0.406725
H	3.908102	0.926732	-1.043015
N	2.433444	1.112251	0.426448
C	4.980787	-1.320550	-0.429195
H	4.279760	-1.604358	-1.215743
C	6.229321	-1.785771	-0.488839
H	6.969924	-1.527288	0.264722
H	6.559369	-2.439942	-1.290299

Energies in solvent (SMD)

SCF = -1192.38253744

Sum of electronic and zero-point Energies= -1191.896613

Sum of electronic and thermal Energies= -1191.871975

Sum of electronic and thermal Enthalpies= -1191.871031

Sum of electronic and thermal Free Energies= -1191.955438

Number of imaginary frequencies:

0

2b - conformer 1 [B3LYP/6-31G(d,p)]

C	0.147498	1.632802	-0.574126
C	0.402927	2.688043	0.311329
H	0.408189	2.498337	1.382215
C	0.647830	3.980328	-0.157301
H	0.843324	4.782147	0.549098
C	0.642348	4.239132	-1.527438
H	0.835010	5.242216	-1.896200
C	0.392720	3.196275	-2.421641
H	0.392851	3.385089	-3.491360
C	0.150042	1.907225	-1.948856
H	-0.022092	1.100820	-2.655747
C	-0.150505	0.220631	-0.055574
H	0.082459	0.223111	1.015466
C	0.762130	-0.809265	-0.719683
C	0.334059	-1.623188	-1.750372
H	-0.693502	-1.595511	-2.089178
C	1.221334	-2.540329	-2.355837
H	0.862334	-3.172046	-3.167683
N	2.478520	-2.701496	-1.996652
C	2.936296	-1.916879	-0.981569
C	4.292636	-2.084915	-0.584038
H	4.874930	-2.833888	-1.110199
C	4.837184	-1.328547	0.418587
H	5.870322	-1.445381	0.728601
C	4.044660	-0.351378	1.078964
C	2.722320	-0.163141	0.726820
H	2.135428	0.598182	1.219381
C	2.129938	-0.942578	-0.307439
O	4.702165	0.349026	2.048105
C	3.990253	1.361318	2.741093
H	3.631589	2.141646	2.057796
H	4.696547	1.797044	3.449162
H	3.135109	0.947582	3.291602
C	-1.674218	-0.072037	-0.178171
H	-1.942007	0.036309	-1.235805
C	-1.859118	-1.722875	1.614692
H	-0.801980	-1.618031	1.874430
H	-2.115958	-2.773038	1.788185
C	-2.757654	-0.775580	2.468978
H	-2.151574	-0.154193	3.139158
H	-3.444120	-1.348495	3.103191
C	-3.551254	0.125636	1.501574
H	-4.196493	0.809895	2.062387
C	-2.540014	0.929115	0.658709
H	-1.914523	1.523545	1.333480
H	-3.047193	1.646809	0.006638
C	-4.426538	-0.787854	0.597809
H	-5.042116	-1.411493	1.258582
C	-3.424988	-1.706698	-0.196773
H	-3.651457	-2.762801	-0.015992
H	-3.526437	-1.538611	-1.275182
N	-2.023498	-1.465822	0.173804
C	-5.354635	-0.030882	-0.310420
H	-4.882224	0.608164	-1.058080
C	-6.686249	-0.099113	-0.276974
H	-7.206737	-0.726645	0.443046
H	-7.306158	0.466785	-0.965973

Energies in solvent (SMD)

SCF = -1192.38311966

Sum of electronic and zero-point Energies= -1191.896829

Sum of electronic and thermal Energies= -1191.872367

Sum of electronic and thermal Enthalpies= -1191.871423

Sum of electronic and thermal Free Energies= -1191.953213

Number of imaginary frequencies:

0

2c - conformer 1 [B3LYP/6-31G(d,p)]

C	-0.866526	2.070947	0.755787
C	-0.171573	2.467730	1.907169
H	0.623414	1.833842	2.293045
C	-0.473914	3.662133	2.559251
H	0.079937	3.945726	3.449743
C	-1.485298	4.488991	2.068518
H	-1.726446	5.419857	2.573337
C	-2.187504	4.105084	0.926390
H	-2.982973	4.735774	0.539319
C	-1.882701	2.907086	0.277743
H	-2.459313	2.620725	-0.594707
C	-0.444123	0.772771	0.063516
H	-0.197124	0.070675	0.862809
C	0.820045	1.000253	-0.772594
C	0.792245	1.903408	-1.819293
H	-0.107789	2.474117	-2.026193
C	1.934469	2.112619	-2.618764
H	1.891042	2.824639	-3.441999
N	3.083261	1.491359	-2.436357
C	3.153850	0.606895	-1.404328
C	4.397253	-0.055308	-1.196901
H	5.206068	0.185566	-1.878448
C	4.563153	-0.952965	-0.177676
H	5.506950	-1.460378	-0.008089
C	3.481355	-1.237867	0.699042
C	2.256814	-0.621976	0.527891
H	1.445775	-0.845628	1.206621
C	2.054582	0.315600	-0.527966
O	3.773252	-2.138696	1.680326
C	2.760543	-2.463757	2.619010
H	1.891676	-2.926315	2.132894
H	3.207708	-3.178982	3.310467
H	2.428743	-1.578785	3.176912
C	-1.543672	0.101299	-0.799737
H	-1.809838	0.806369	-1.595231
C	-2.668847	-1.287836	0.852974
H	-1.798971	-1.143616	1.500819
H	-3.542536	-1.314994	1.512530
C	-2.541642	-2.635806	0.055993
H	-3.509030	-3.154441	0.074952
C	-2.253119	-2.234289	-1.419129
H	-2.015027	-3.126040	-2.007795
C	-1.080198	-1.231233	-1.470483
H	-0.202338	-1.646919	-0.964341
H	-0.776873	-1.054586	-2.507574
C	-3.519132	-1.543692	-1.967898
H	-4.343959	-2.261238	-2.044759
C	-3.880866	-0.376539	-0.997334
H	-4.783837	-0.607127	-0.421797
H	-4.081944	0.549029	-1.548287
N	-2.792893	-0.122486	-0.036356
H	-3.327489	-1.169062	-2.980395
C	-1.521451	-3.562307	0.653929
C	-1.784618	-4.763218	1.171658
H	-0.491031	-3.202600	0.667878
H	-1.004664	-5.386949	1.598279
H	-2.793470	-5.169344	1.185793

Energies in solvent (SMD)

SCF = -1192.38192999

Sum of electronic and zero-point Energies= -1191.895873

Sum of electronic and thermal Energies= -1191.872260

Sum of electronic and thermal Enthalpies= -1191.871316

Sum of electronic and thermal Free Energies= -1191.950242

Number of imaginary frequencies:

1 (-20 cm^{-1})

Frequencies in vacuum

Number of imaginary frequencies:

0

2c - conformer 2 [B3LYP/6-31G(d,p)]

C	0.114562	1.854656	-0.017191
C	0.050810	2.365973	1.285069
H	-0.251174	1.713722	2.100771
C	0.356505	3.700600	1.550984
H	0.299488	4.074526	2.569467
C	0.730296	4.552879	0.511358
H	0.970511	5.592391	0.714485
C	0.796336	4.056761	-0.791045
H	1.090920	4.709457	-1.607995
C	0.495140	2.719354	-1.050782
H	0.571736	2.342565	-2.066384
C	-0.244491	0.391801	-0.293413
H	-0.223070	-0.131417	0.669221
C	0.803456	-0.281757	-1.178804
C	0.629963	-0.451229	-2.539617
H	-0.276058	-0.119009	-3.033841
C	1.628035	-1.076138	-3.321673
H	1.466192	-1.202520	-4.391652
N	2.762861	-1.536375	-2.838353
C	2.978971	-1.372671	-1.502686
C	4.208309	-1.856176	-0.973201
H	4.892465	-2.336751	-1.664384
C	4.514042	-1.710997	0.353278
H	5.450005	-2.070143	0.768110
C	3.597968	-1.063895	1.226085
C	2.388562	-0.593149	0.752178
H	1.710260	-0.078233	1.416951
C	2.043513	-0.740603	-0.620579
O	4.024162	-0.959635	2.516531
C	3.180018	-0.302648	3.449905
H	2.222823	-0.827426	3.565463
H	3.714439	-0.315666	4.400676
H	2.985314	0.736131	3.155243
C	-1.695857	0.249566	-0.832769
H	-1.747573	0.810952	-1.773027
C	-2.973137	0.092607	1.242000
H	-2.034079	-0.146218	1.750543
H	-3.559499	0.695914	1.943169
C	-3.740457	-1.227240	0.877995
H	-4.805118	-1.101141	1.114499
C	-3.616252	-1.373820	-0.664163
H	-4.004476	-2.348132	-0.978094
C	-2.139554	-1.225828	-1.090405
H	-1.506277	-1.927313	-0.534620
H	-2.027822	-1.490516	-2.146356
C	-4.430988	-0.232099	-1.307331
H	-5.501334	-0.370961	-1.117007
C	-3.922364	1.113687	-0.700090
H	-4.662575	1.541873	-0.015447
H	-3.739662	1.859637	-1.480819
N	-2.674536	0.915842	0.059026
H	-4.296584	-0.248201	-2.395573
C	-3.241165	-2.411296	1.656266
C	-3.975257	-3.139908	2.498654
H	-2.189927	-2.670692	1.522002
H	-3.556232	-3.981339	3.042402
H	-5.026029	-2.921242	2.675088

Energies in solvent (SMD)

SCF = -1192.38305994

Sum of electronic and zero-point Energies= -1191.896593

Sum of electronic and thermal Energies= -1191.872186

Sum of electronic and thermal Enthalpies= -1191.871242

Sum of electronic and thermal Free Energies= -1191.952513

Number of imaginary frequencies:

0

2d - conformer 1 [B3LYP/6-31G(d,p)]

C	0.146283	1.527018	-0.974424
C	0.172896	2.661073	-0.151957
H	-0.068045	2.561395	0.904021
C	0.500365	3.917560	-0.664731
H	0.514703	4.781808	-0.006676
C	0.808632	4.060960	-2.017209
H	1.066262	5.036038	-2.419851
C	0.789749	2.938912	-2.847835
H	1.035654	3.037937	-3.901252
C	0.464367	1.685459	-2.330385
H	0.474753	0.817207	-2.982826
C	-0.249814	0.156800	-0.412882
H	-0.255546	0.253471	0.679217
C	0.787084	-0.908082	-0.770107
C	0.584339	-1.833107	-1.775484
H	-0.349897	-1.866751	-2.321477
C	1.582753	-2.780280	-2.092834
H	1.400374	-3.499541	-2.890667
N	2.742970	-2.867616	-1.475352
C	2.978724	-1.973093	-0.475351
C	4.228070	-2.060940	0.200726
H	4.912025	-2.839821	-0.119324
C	4.550351	-1.195299	1.210942
H	5.499962	-1.250926	1.732730
C	3.630460	-0.183689	1.597485
C	2.405713	-0.069147	0.969030
H	1.722846	0.717640	1.254278
C	2.043632	-0.961619	-0.079712
O	4.070356	0.624753	2.604744
C	3.220219	1.673394	3.040085
H	2.995937	2.374937	2.226477
H	3.763858	2.195615	3.828640
H	2.277428	1.284910	3.447153
C	-1.704684	-0.188496	-0.840696
H	-1.711754	-0.253293	-1.934872
C	-2.498023	-1.481803	1.066160
H	-1.645624	-1.090800	1.630228
H	-2.650604	-2.509448	1.411978
C	-3.781101	-0.615797	1.335552
H	-4.638835	-1.283884	1.488278
C	-4.038959	0.183628	0.026213
H	-4.836965	0.915260	0.188858
C	-2.743507	0.896760	-0.414998
H	-2.357880	1.515214	0.402414
H	-2.947207	1.582803	-1.243612
C	-4.444563	-0.828302	-1.065304
H	-5.419056	-1.272814	-0.833253
C	-3.332219	-1.919950	-1.133115
H	-3.687333	-2.871539	-0.723184
H	-3.028825	-2.112261	-2.168522
N	-2.141918	-1.519543	-0.361057
H	-4.550320	-0.312981	-2.027413
C	-3.649071	0.236436	2.565623
C	-4.436240	0.160273	3.639818
H	-2.830334	0.957821	2.569532
H	-4.286642	0.797866	4.506055
H	-5.261883	-0.545786	3.692325

Energies in solvent (SMD)

SCF = -1192.38356500

Sum of electronic and zero-point Energies= -1191.897209

Sum of electronic and thermal Energies= -1191.872807

Sum of electronic and thermal Enthalpies= -1191.871863

Sum of electronic and thermal Free Energies= -1191.953199

Number of imaginary frequencies: 0

3a - conformer 1 [mPW1PW91/6-311+G(2d,p)]

C	0.259217	0.344363	-1.313105
C	-0.758041	-0.814178	-1.274837
C	-0.738113	-1.735455	-2.299774
C	-1.667595	-2.791460	-2.339716
N	-2.609055	-2.969002	-1.443975
C	-2.665903	-2.081842	-0.417051
C	-3.686340	-2.278702	0.550669
C	-3.810663	-1.451329	1.626005
C	-2.903962	-0.376032	1.793981
C	-1.905645	-0.148220	0.876620
C	-1.759513	-0.984166	-0.263549
O	-3.113149	0.371266	2.913557
C	-2.265968	1.491028	3.141976
C	1.577009	0.114903	-0.485802
C	1.189390	-1.369066	1.445688
C	2.345106	-2.339569	1.077438
C	3.304172	-1.583279	0.147875
C	2.476032	-1.027969	-1.028129
C	3.949788	-0.441538	0.979536
C	2.761875	0.420881	1.547526
N	1.460855	0.012927	0.988366
C	4.950790	0.375472	0.217100
C	6.242923	0.468215	0.514129
O	0.727527	0.395868	-2.687879
H	-0.023565	-1.636933	-3.102755
H	-1.629157	-3.506306	-3.157463
H	-4.357511	-3.114578	0.399805
H	-4.585624	-1.594465	2.368724
H	-1.202265	0.650683	1.035099
H	-1.222924	1.182362	3.255608
H	-2.615482	1.943176	4.067736
H	-2.341235	2.215806	2.326607
H	2.121177	1.047653	-0.654532
H	0.253134	-1.703921	1.012943
H	1.037134	-1.326129	2.526181
H	1.954319	-3.227511	0.574270
H	2.874763	-2.684169	1.970295
H	4.086026	-2.248221	-0.224632
H	1.878522	-1.838492	-1.451295
H	3.112883	-0.661752	-1.833015
H	4.477975	-0.906762	1.817396
H	2.709170	0.320755	2.633824
H	2.924352	1.479886	1.331057
H	4.576197	0.937962	-0.636342
H	6.671778	-0.067156	1.355439
H	6.918911	1.079979	-0.071368
C	-0.409354	1.706145	-1.053378
C	-1.525615	2.043636	-1.828624
C	0.087099	2.659606	-0.163448
C	-2.126916	3.289454	-1.716559
H	-1.924066	1.319329	-2.528372
C	-0.515779	3.911314	-0.050028
H	0.926386	2.425417	0.474555
C	-1.623181	4.231965	-0.823849
H	-2.992003	3.524323	-2.325422
H	-0.113935	4.634556	0.650198
H	-2.091140	5.205106	-0.733816
H	1.013670	1.299797	-2.861038

Energies in solvent (SMD)

SCF = -1267.60595955

Sum of electronic and zero-point Energies= -1267.115934

Sum of electronic and thermal Energies= -1267.090569

Sum of electronic and thermal Enthalpies= -1267.089625

Sum of electronic and thermal Free Energies= -1267.169544

Number of imaginary frequencies:

0

3a - conformer 2 [mPW1PW91/6-311+G(2d,p)]

C	0.174748	1.231091	-0.829705
C	-1.187951	0.659379	-1.317280
C	-1.659684	1.077455	-2.540862
C	-2.844243	0.545659	-3.082811
N	-3.570371	-0.371949	-2.490442
C	-3.155541	-0.796333	-1.269077
C	-3.958705	-1.771125	-0.619764
C	-3.649265	-2.230500	0.624569
C	-2.508416	-1.727387	1.296260
C	-1.691348	-0.799126	0.691465
C	-1.980550	-0.307918	-0.614440
O	-2.328354	-2.232300	2.548948
C	-1.239627	-1.755441	3.326719
C	1.158586	-0.009793	-0.507120
C	3.407774	0.611923	-1.330873
C	4.143170	0.119339	-0.056152
C	3.198474	-0.851274	0.666399
C	1.856292	-0.115058	0.869834
C	3.043728	-2.107153	-0.237982
C	2.533166	-1.597395	-1.635291
N	2.171644	-0.169082	-1.585520
C	2.164252	-3.173170	0.348290
C	2.582123	-4.372036	0.740751
O	0.763484	1.965829	-1.897845
H	-1.106332	1.817183	-3.100024
H	-3.190684	0.891214	-4.053229
H	-4.833941	-2.124068	-1.150192
H	-4.262251	-2.965690	1.130641
H	-0.846066	-0.403586	1.229578
H	-0.281036	-2.016525	2.868776
H	-1.322834	-2.251429	4.291307
H	-1.295262	-0.672224	3.467470
H	0.530234	-0.891016	-0.607494
H	3.135007	1.661668	-1.250569
H	4.039114	0.513730	-2.216423
H	4.393231	0.960492	0.595340
H	5.082131	-0.381055	-0.308398
H	3.613781	-1.148420	1.631326
H	2.055589	0.861255	1.313227
H	1.221073	-0.649615	1.577701
H	4.042150	-2.537469	-0.361722
H	3.307339	-1.725216	-2.394668
H	1.661418	-2.166989	-1.961712
H	1.105042	-2.939422	0.439649
H	3.624487	-4.664498	0.661294
H	1.898721	-5.105701	1.151327
C	0.013776	2.253205	0.308312
C	-1.166324	2.424056	1.031976
C	1.083681	3.108826	0.598017
C	-1.263587	3.388039	2.034183
H	-2.034910	1.821475	0.811827
C	0.991095	4.070395	1.594771
H	1.990051	3.040117	0.012619
C	-0.184849	4.210419	2.326322
H	-2.196041	3.499916	2.575057
H	1.835958	4.719782	1.792998
H	-0.261822	4.962881	3.102223
H	1.241264	1.295736	-2.424085

Energies in solvent (SMD)

SCF = -1267.60510711

Sum of electronic and zero-point Energies= -1267.114524

Sum of electronic and thermal Energies= -1267.089528

Sum of electronic and thermal Enthalpies= -1267.088584

Sum of electronic and thermal Free Energies= -1267.168329

Number of imaginary frequencies:

0

3b - conformer 1 [mPW1PW91/6-311+G(2d,p)]

C	-0.097119	1.004338	-0.946738
C	0.826762	-0.113359	-1.500663
C	0.864187	-0.338224	-2.856849
C	1.643343	-1.381020	-3.393639
N	2.364640	-2.200349	-2.666861
C	2.364775	-2.004525	-1.322245
C	3.155899	-2.886101	-0.539681
C	3.235257	-2.753268	0.814293
C	2.524083	-1.714315	1.462876
C	1.738663	-0.845418	0.740837
C	1.624612	-0.966928	-0.672015
O	2.700174	-1.664692	2.812192
C	2.041963	-0.637654	3.542011
C	-1.278381	0.291372	-0.208549
C	-3.272188	1.727683	-0.517326
C	-4.072949	0.599936	-1.229799
C	-3.501804	-0.749235	-0.766126
C	-1.999908	-0.763423	-1.101762
C	-3.740697	-0.870152	0.762704
C	-3.009288	0.356294	1.420306
N	-2.286111	1.172316	0.432363
C	-3.301311	-2.181577	1.341796
C	-4.102321	-3.063597	1.930938
O	-0.681662	1.723545	-2.052363
H	0.277019	0.280909	-3.519579
H	1.655994	-1.541365	-4.468558
H	3.697293	-3.663898	-1.063062
H	3.840422	-3.418307	1.417632
H	1.230693	-0.041345	1.246088
H	2.354791	0.353841	3.202959
H	2.333994	-0.777192	4.580505
H	0.954696	-0.725627	3.455404
H	-0.811738	-0.240037	0.621238
H	-2.747239	2.350231	-1.234335
H	-3.936675	2.374731	0.060205
H	-3.978153	0.687273	-2.315163
H	-5.139075	0.661324	-0.992861
H	-4.004001	-1.572721	-1.278343
H	-1.880385	-0.524537	-2.159619
H	-1.565388	-1.753057	-0.951621
H	-4.817245	-0.776449	0.935678
H	-3.733486	1.000317	1.924315
H	-2.299466	0.016648	2.179254
H	-2.238966	-2.410499	1.276951
H	-5.168973	-2.886273	2.027403
H	-3.722863	-3.993986	2.336417
C	0.726439	2.019177	-0.128767
C	0.285926	2.609230	1.058718
C	1.962014	2.428039	-0.648719
C	1.064128	3.565154	1.707254
H	-0.681269	2.335432	1.454431
C	2.731869	3.391177	-0.007567
H	2.339755	1.973484	-1.557273
C	2.286843	3.962356	1.179830
H	0.702172	4.008100	2.628169
H	3.685346	3.684319	-0.430928
H	2.887071	4.708682	1.686756
H	-0.136905	2.499646	-2.225907

Energies in solvent (SMD)

SCF = -1267.60961191

Sum of electronic and zero-point Energies= -1267.119875

Sum of electronic and thermal Energies= -1267.094466

Sum of electronic and thermal Enthalpies= -1267.093522

Sum of electronic and thermal Free Energies= -1267.173866

Number of imaginary frequencies:

0

3b - conformer 2 [mPW1PW91/6-311+G(2d,p)]

C	-0.250472	0.800208	-0.380773
C	0.827292	0.675863	0.728690
C	0.479881	1.192040	1.960887
C	1.338451	1.090386	3.065577
N	2.517551	0.516742	3.014130
C	2.907145	0.001436	1.821354
C	4.185864	-0.616182	1.784258
C	4.672129	-1.173405	0.641139
C	3.891066	-1.139788	-0.539291
C	2.648729	-0.552816	-0.550576
C	2.108575	0.041795	0.627276
O	4.479200	-1.725618	-1.621740
C	3.772149	-1.726350	-2.853625
C	-1.360587	-0.251597	-0.056666
C	-2.237337	-1.107522	-2.207729
C	-1.998735	-2.597883	-1.831937
C	-1.924038	-2.682476	-0.300537
C	-0.836827	-1.695487	0.168735
C	-3.321357	-2.298639	0.257257
C	-3.642195	-0.861256	-0.300354
N	-2.495337	-0.274011	-1.013027
C	-3.420002	-2.378597	1.751804
C	-4.232959	-3.190253	2.420025
O	0.328516	0.530821	-1.671037
H	-0.463552	1.706252	2.091917
H	1.035481	1.505934	4.022859
H	4.755410	-0.625212	2.704752
H	5.646273	-1.645060	0.605544
H	2.070965	-0.520866	-1.454806
H	2.827767	-2.272334	-2.769283
H	4.419959	-2.229322	-3.568482
H	3.570401	-0.707182	-3.196198
H	-1.793108	0.088888	0.886218
H	-1.379345	-0.708402	-2.739799
H	-3.104167	-1.000335	-2.863344
H	-1.067118	-2.962508	-2.272395
H	-2.803341	-3.235628	-2.209185
H	-1.671460	-3.696300	0.016353
H	0.072706	-1.878311	-0.407224
H	-0.579644	-1.851267	1.217374
H	-4.044808	-3.006645	-0.158419
H	-4.485493	-0.904847	-0.993186
H	-3.932751	-0.193258	0.514442
H	-2.777799	-1.708327	2.320349
H	-4.897906	-3.877522	1.906236
H	-4.263455	-3.198916	3.503025
C	-0.755712	2.256262	-0.416943
C	-2.053143	2.648985	-0.091264
C	0.157106	3.245448	-0.805417
C	-2.427067	3.990231	-0.149251
H	-2.792791	1.914601	0.188631
C	-0.215591	4.581137	-0.869568
H	1.173774	2.962960	-1.050920
C	-1.513641	4.960466	-0.539314
H	-3.442131	4.270964	0.106363
H	0.510749	5.327552	-1.168976
H	-1.807649	6.002330	-0.585091
H	-0.186557	1.029993	-2.314820

Energies in solvent (SMD)

SCF = -1267.60699133

Sum of electronic and zero-point Energies= -1267.117158

Sum of electronic and thermal Energies= -1267.091805

Sum of electronic and thermal Enthalpies= -1267.090861

Sum of electronic and thermal Free Energies= -1267.171387

Number of imaginary frequencies: 0

3c - conformer 1 [mPW1PW91/6-311+G(2d,p)]

C	-0.061680	-0.336952	-0.380483
C	1.058457	-1.209694	0.229333
C	0.782370	-2.353636	0.942392
C	1.814997	-3.120142	1.518825
N	3.084897	-2.813773	1.429210
C	3.406225	-1.691111	0.732734
C	4.783170	-1.360207	0.640751
C	5.198129	-0.251162	-0.033660
C	4.245861	0.589117	-0.660279
C	2.902838	0.301995	-0.596393
C	2.440041	-0.843646	0.103943
O	4.779660	1.661535	-1.310305
C	3.893381	2.552804	-1.974412
C	-1.446477	-1.121272	-0.448242
C	-3.079678	-1.797882	-2.057391
C	-3.904968	-2.343556	-0.855044
C	-3.832977	-1.302031	0.277815
C	-2.377441	-1.197572	0.785524
C	-4.270806	0.058290	-0.322184
C	-3.123719	0.476012	-1.295594
N	-2.238423	-0.662602	-1.623864
C	-4.564645	1.120681	0.694093
C	-5.743483	1.713140	0.852975
O	0.284505	-0.092252	-1.740671
H	-0.229884	-2.698610	1.088950
H	1.564882	-4.019484	2.075986
H	5.487686	-2.022832	1.127054
H	6.246337	0.010044	-0.109910
H	2.187148	0.925828	-1.100949
H	3.333240	2.040184	-2.761775
H	4.522423	3.322505	-2.416683
H	3.192790	3.011331	-1.270628
H	-1.145166	-2.134277	-0.717913
H	-2.436098	-2.569140	-2.484129
H	-3.731742	-1.439767	-2.856780
H	-3.505219	-3.301330	-0.510137
H	-4.943632	-2.518983	-1.145638
H	-4.496441	-1.580367	1.098505
H	-2.137209	-2.068077	1.399464
H	-2.271204	-0.326125	1.431277
H	-5.188028	-0.112975	-0.894987
H	-3.533362	0.868618	-2.228511
H	-2.521578	1.265754	-0.852118
H	-3.736722	1.427901	1.330015
H	-6.599143	1.452152	0.238052
H	-5.896422	2.480882	1.601958
C	-0.141434	0.990915	0.404152
C	-0.119073	1.009245	1.801477
C	-0.220944	2.211399	-0.267960
C	-0.198508	2.206731	2.504539
H	-0.015965	0.081982	2.351358
C	-0.300442	3.411490	0.432254
H	-0.205203	2.216907	-1.349680
C	-0.293973	3.414971	1.822456
H	-0.174156	2.194136	3.587947
H	-0.361800	4.346505	-0.112788
H	-0.350921	4.348961	2.368683
H	-0.564578	-0.188951	-2.225965

Energies in solvent (SMD)

SCF = -1267.61548151

Sum of electronic and zero-point Energies= -1267.124704

Sum of electronic and thermal Energies= -1267.099869

Sum of electronic and thermal Enthalpies= -1267.098925

Sum of electronic and thermal Free Energies= -1267.177890

Number of imaginary frequencies:

0

3c - conformer 2 [mPW1PW91/6-311+G(2d,p)]

C	-0.134052	0.688103	-1.015705
C	1.350939	0.374682	-1.364204
C	1.777024	0.638930	-2.646243
C	3.082872	0.316498	-3.058488
N	3.973130	-0.255086	-2.283176
C	3.603702	-0.513307	-1.002328
C	4.578418	-1.110982	-0.159700
C	4.317740	-1.374938	1.150955
C	3.053269	-1.044114	1.696051
C	2.074855	-0.483626	0.907189
C	2.310959	-0.206710	-0.470668
O	2.924636	-1.320082	3.024451
C	1.705316	-0.989007	3.672385
C	-0.811138	-0.641880	-0.405539
C	-1.633046	-2.761116	-1.123925
C	-2.000482	-3.000010	0.370994
C	-2.641023	-1.706507	0.914562
C	-1.591215	-0.571142	0.928309
C	-3.783023	-1.323918	-0.059571
C	-3.070122	-0.859676	-1.367758
N	-1.660596	-1.314248	-1.427098
C	-4.730955	-0.290693	0.468630
C	-6.041811	-0.460516	0.606673
O	-0.824968	0.993281	-2.222404
H	1.094599	1.090194	-3.351027
H	3.390776	0.533364	-4.077950
H	5.541194	-1.336993	-0.600095
H	5.058744	-1.821858	1.801763
H	1.129607	-0.211808	1.346496
H	1.492081	0.081224	3.597113
H	1.839576	-1.259624	4.717411
H	0.868468	-1.558027	3.256011
H	0.013757	-1.332121	-0.251716
H	-0.641840	-3.152263	-1.358774
H	-2.343285	-3.254150	-1.790964
H	-1.110347	-3.254085	0.953401
H	-2.695103	-3.837825	0.470394
H	-3.039456	-1.867023	1.918063
H	-0.909671	-0.694824	1.772979
H	-2.079862	0.391969	1.072547
H	-4.364159	-2.228594	-0.265557
H	-3.589560	-1.249242	-2.245894
H	-3.081522	0.225986	-1.447094
H	-4.299216	0.669012	0.745691
H	-6.525593	-1.395035	0.340397
H	-6.679355	0.327429	0.989393
C	-0.279712	1.948912	-0.145289
C	0.770464	2.515037	0.577808
C	-1.517381	2.603121	-0.114445
C	0.583197	3.670587	1.334269
H	1.756246	2.075664	0.546907
C	-1.707512	3.754840	0.637279
H	-2.331708	2.222440	-0.715167
C	-0.656758	4.292328	1.374873
H	1.420454	4.089059	1.880602
H	-2.676408	4.240759	0.636639
H	-0.800796	5.193371	1.959222
H	-1.085848	0.123398	-2.581918

Energies in solvent (SMD)

SCF = -1267.60645455

Sum of electronic and zero-point Energies= -1267.115897

Sum of electronic and thermal Energies= -1267.090954

Sum of electronic and thermal Enthalpies= -1267.090010

Sum of electronic and thermal Free Energies= -1267.169545

Number of imaginary frequencies:

0

3d - conformer 1 [mPW1PW91/6-311+G(2d,p)]

C	0.321353	0.310684	-0.928089
C	-0.586017	-0.937329	-1.094392
C	-0.275648	-1.864260	-2.061602
C	-1.038997	-3.038773	-2.205211
N	-2.067230	-3.337397	-1.447972
C	-2.413729	-2.441876	-0.486335
C	-3.533087	-2.763042	0.325621
C	-3.966102	-1.916285	1.301875
C	-3.298328	-0.685565	1.513900
C	-2.204039	-0.341908	0.753894
C	-1.719554	-1.211092	-0.262649
O	-3.835572	0.088551	2.497217
C	-3.241327	1.352218	2.761895
C	1.112394	0.123888	0.409272
C	2.263851	1.127000	2.233767
C	2.721870	-0.302327	2.647470
C	3.113888	-1.053608	1.362266
C	1.855857	-1.241947	0.492554
C	4.148368	-0.178103	0.607866
C	3.362684	1.081710	0.111480
N	2.051430	1.213542	0.775664
C	4.850709	-0.881650	-0.513068
C	6.162621	-1.084555	-0.583102
O	1.300388	0.322421	-1.988495
H	0.571841	-1.697490	-2.710462
H	-0.774639	-3.758385	-2.975647
H	-4.031185	-3.705068	0.134587
H	-4.821882	-2.151826	1.922137
H	-1.729204	0.613142	0.903919
H	-2.204739	1.239783	3.093973
H	-3.830577	1.796352	3.561162
H	-3.272328	1.999124	1.880727
H	0.351630	0.134809	1.190264
H	1.338716	1.408293	2.744129
H	3.016591	1.872554	2.499863
H	1.919429	-0.838904	3.162185
H	3.567957	-0.255209	3.338046
H	3.552209	-2.023968	1.603507
H	1.204552	-2.003499	0.926161
H	2.134558	-1.596071	-0.500291
H	4.909879	0.129450	1.332493
H	3.935675	1.989211	0.315855
H	3.196970	1.031068	-0.960387
H	4.225454	-1.230257	-1.332510
H	6.832965	-0.747690	0.201526
H	6.614853	-1.592986	-1.426414
C	-0.511143	1.600398	-1.068003
C	-1.471953	1.652335	-2.086705
C	-0.312929	2.744885	-0.291655
C	-2.210595	2.805700	-2.322943
H	-1.659748	0.773990	-2.693187
C	-1.062593	3.896030	-0.520758
H	0.453364	2.735466	0.469529
C	-2.011395	3.934616	-1.535558
H	-2.949390	2.816356	-3.115617
H	-0.891953	4.772036	0.094660
H	-2.589132	4.834125	-1.712670
H	0.983250	0.911943	-2.681901

Energies in solvent (SMD)

SCF = -1267.61045435

Sum of electronic and zero-point Energies= -1267.120661

Sum of electronic and thermal Energies= -1267.095303

Sum of electronic and thermal Enthalpies= -1267.094359

Sum of electronic and thermal Free Energies= -1267.174507

Number of imaginary frequencies:

0

3d - conformer 2 [mPW1PW91/6-311+G(2d,p)]

C	-0.608379	-0.728490	-0.261253
C	0.615266	-1.186194	0.572033
C	0.344527	-2.072686	1.594906
C	1.353169	-2.521497	2.460353
N	2.608166	-2.151144	2.361450
C	2.924584	-1.285877	1.365806
C	4.287471	-0.896389	1.272976
C	4.713324	-0.025653	0.316951
C	3.782372	0.507246	-0.607334
C	2.454030	0.159229	-0.555411
C	1.973759	-0.752936	0.429501
O	4.322271	1.365320	-1.519931
C	3.465509	1.931283	-2.501179
C	-1.419661	0.276349	0.618856
C	-3.558143	1.238137	1.008930
C	-2.855907	2.130248	2.076489
C	-1.528279	2.624631	1.471052
C	-0.587905	1.419290	1.257647
C	-1.867508	3.265714	0.101319
C	-2.284348	2.075600	-0.823776
N	-2.609736	0.854006	-0.056872
C	-0.759986	4.080560	-0.492465
C	-0.845763	5.367192	-0.814983
O	-0.172889	-0.090118	-1.475390
H	-0.658081	-2.455845	1.735015
H	1.107779	-3.218148	3.257420
H	4.971638	-1.321526	1.996152
H	5.750838	0.274677	0.239389
H	1.758220	0.557795	-1.269591
H	3.002581	1.156324	-3.118978
H	4.098680	2.562083	-3.121503
H	2.682606	2.541276	-2.040738
H	-1.815338	-0.329327	1.436694
H	-3.972343	0.334441	1.463278
H	-4.389604	1.766866	0.537470
H	-2.661962	1.565374	2.992924
H	-3.489701	2.976748	2.352889
H	-1.057744	3.360940	2.125149
H	-0.161152	1.093603	2.208053
H	0.250500	1.703066	0.619488
H	-2.725134	3.930888	0.246183
H	-3.160375	2.344691	-1.418129
H	-1.477278	1.851116	-1.515912
H	0.176460	3.558588	-0.679490
H	-1.757465	5.934168	-0.654700
H	-0.009186	5.901422	-1.249479
C	-1.412154	-1.974910	-0.686609
C	-0.787504	-2.876976	-1.557089
C	-2.712427	-2.255551	-0.270442
C	-1.440341	-4.020646	-1.995721
H	0.225124	-2.679471	-1.887148
C	-3.368130	-3.405692	-0.705943
H	-3.238696	-1.574217	0.380415
C	-2.737724	-4.291473	-1.569227
H	-0.933042	-4.705127	-2.665424
H	-4.379894	-3.601075	-0.370227
H	-3.249823	-5.184445	-1.907423
H	-0.910788	-0.149580	-2.093565

Energies in solvent (SMD)

SCF = -1267.60817842

Sum of electronic and zero-point Energies= -1267.118010

Sum of electronic and thermal Energies= -1267.092762

Sum of electronic and thermal Enthalpies= -1267.091818

Sum of electronic and thermal Free Energies= -1267.171997

Number of imaginary frequencies:

0
